# Patterns of change and continuity in ochre use during the late Middle Stone Age of the Horn of Africa: The Porc-Epic Cave record

**DOI:** 10.1371/journal.pone.0177298

**Published:** 2017-05-24

**Authors:** Daniela Eugenia Rosso, Francesco d’Errico, Alain Queffelec

**Affiliations:** 1 Seminari d'Estudis i Recerques Prehistòriques (SERP), Departament d’Història i Arqueologia, Universitat de Barcelona, Barcelona, Spain; 2 UMR-CNRS 5199 de la Préhistoire à l'Actuel: Culture, Environnement et Anthropologie (PACEA), Université de Bordeaux, CNRS, Pessac, France; 3 Evolutionary Studies Institute and DST/NRF Centre of Excellence in Palaeosciences, and School of Geosciences, University of the Witwatersrand, Johannesburg, South Africa; Universidade do Algarve, PORTUGAL

## Abstract

Ochre is found at numerous Middle Stone Age (MSA) sites and plays a key role in early modern human archaeology. Here we analyse the largest known East African MSA ochre assemblage, comprising 40 kg of ochre, found at Porc-Epic Cave, Ethiopia, spanning a period of at least 4,500 years. Visual characterisation of ochre types, microscopic identification of traces of modification, morphological and morphometric analysis of ochre pieces and modified areas, experimental reproduction of grinding processes, surface texture analysis of archaeological and experimentally ground ochre facets, laser granulometry of ochre powder produced experimentally on different grindstones and by Hamar and Ovahimba women from Ethiopia and Namibia respectively, were, for the first time, combined to explore diachronic shifts in ochre processing technology. Our results identify patterns of continuity in ochre acquisition, treatment and use reflecting both persistent use of the same geological resources and similar uses of iron-rich rocks by late MSA Porc-Epic inhabitants. Considering the large amount of ochre processed at the site, this continuity can be interpreted as the expression of a cohesive cultural adaptation, largely shared by all community members and consistently transmitted through time. A gradual shift in preferred processing techniques and motions is interpreted as reflecting cultural drift within this practice. Evidence for the grinding of ochre to produce small quantities of powder throughout the sequence is consistent with a use in symbolic activities for at least part of the ochre assemblage from Porc-Epic Cave.

## Introduction

Ochre pieces, often modified by grinding and scraping to produce red powder, and ochre-stained objects (grindstones, ochre containers, lithic and bone tools, personal ornaments) represent one of the most controversial features found at Middle Stone Age (MSA) and Middle Palaeolithic sites. It is often argued that such innovation reflects cognitive complexity [1], and many consider ochre as a marker of symbolically mediated behaviour [2–13]. Some authors consider that body painting in the earliest group rituals were primarily indexical and that only once ochre use became ubiquitous, such use was part of “symbolic culture” [5,14–18]. However, some have argued that inferring symbolism from such an equivocal archaeological feature is risky, preferring functional explanations such as hide tanning, adhesive production, insect repellent, antiseptic treatments, or sun protection as viable alternative interpretations [1,19–30]. Still others contend that there has been an unnecessary polarisation around the distinction between symbolic and utilitarian activities that fails to account for the complex interplay between functional and symbolic activities in traditional human cultures [31].

Although numerous African MSA sites yielded ochre pieces [3–6,15,16,32–37], in some cases associated with coloured artefacts [38,39], detailed reconstructions of MSA ochre processing techniques are rare, particularly in East Africa. This is due to the fact that effective methodologies for addressing technological aspects of modified ochre pieces have only been developed recently [3,4,21,33,34,40] and that few multistratified sites from Africa have yielded ochre collections large enough to produce reliable results.

Here we present the first technological analysis of ochre pieces from Porc-Epic Cave, Dire Dawa, Ethiopia. This site has yielded the largest collection of ochre currently known in East Africa, weighting 40 kg (n = 4213 pieces), found during excavations of 49 m^2^ over a depth of approximately 3 m. Previous work has shown that, while found throughout the stratigraphic sequence, ochre pieces are concentrated in different locations and depths [38,41]. Analysis of 23 associated ochre processing tools and ochre-stained artefacts has demonstrated that different types of rocks, sometimes exogenous, were used to process ochre. Additionally, it was shown that a variety of ochre types were processed and that different processing techniques were involved, suggesting that different shades and colours of ochre were possibly intended for variety of activities. This study focuses on reconstructing the sequence of actions that led to the accumulation, transformation and use of iron-rich rocks at this site. Such an approach permits the function of these objects and, ultimately, the significance of ochre use for late MSA groups of the Horn of Africa to be explored. We equally address to what extent these practices changed through time and for what reasons by combining morphometric, technological, and roughness analyses with experimental and ethnographic data.

### Background

The term “ochre” refers to a variety of rocks characterised by a red or yellow colour or streak, from soil lumps to ore minerals, containing a high proportion of iron oxides [21,42,43]. Yellow ochres usually derive their colour from goethite (α -FeOOH), red ochre from hematite (α-Fe_2_O_3_), and often contain other components such as quartz, clays, gypsum, or mica [44]. Ochre is frequently found at Middle Stone Age sites, particularly after 100 ka BP, when it becomes ubiquitous [37,39]. Numerous MSA sites from East Africa have yielded ochre, including the Kapthurin Formation [37,45], Enkapune Ya Muto [46] in Kenya, Mumba and Nasera Rock Shelters [47] in Tanzania, Mochena Borago Rock Shelter [48], Gorgora Rock Shelter [49–51] and Aduma [52] in Ethiopia. However, none of these ochre collections have been analysed, and comprehensive studies of MSA ochre pieces, with the exception of Twin Rivers and Mumbwa in Zambia [32,53], are currently limited to material from South African sites (e.g. Blombos Cave, Pinnacle Point Cave, Diepkloof Rock Shelter and Sibudu Cave).

Blombos Cave is probably one of the richest sites with respect to ochre use. Dated to ca. 100–72 ka, the site’s MSA levels yielded a collection of more than 8000 ochre pieces weighing approximately 5.8 kg [5,33,54–56]. Elemental and mineralogical analysis of pieces from different layers show clear differences in composition, suggesting they come from different geological sources, and that ochre procurement patterns changed through time [5,56]. Colour profiles, identified using the Natural Color System Index, detected a variety of shades, with a clear preference for saturated red ochre [5]. Striations, grooves, scraping, percussion pits, possible traces of handling, as well as abstract engravings, were identified on numerous pieces [5,21,33,54,55]. Watts [5] concluded that the reddest and more saturated pieces were overrepresented among the most intensively ground pieces, and that ochre was ground to produce small quantities of powder, a behaviour consistent with a use for symbolic activities. This is supported by the presence of numerous intensively ground pieces, as well as twelve “definite” and twelve “probable” ochre pieces used as “crayons”, following the definition according to which a crayon is an ochre piece characterised by three or more facets converging to a point [5,20,55]. Differences were observed in the sequence: harder forms of ochre (highly ferruginous types of ochre from distant sources), and intensively ground pieces (“crayons”) were better represented in younger levels. The latter, according to Henshilwood et al. [55], were probably the result of a more protracted processing, consistent with curation. This appears to be in accordance with changes in procurement of raw material, which shift to more distant sources [55]. Two toolkits for the production and storage of ochre-rich compounds were also recovered from layers dated to 100 ka [35]. These toolkits comprise modified ochre pieces, bones, upper and lower grindstones, and two abalone shells still containing an ochre-rich compound.

The analysis of 380 ochre pieces (1.08 kg) from layers dated to ca. 164–91 ka at Pinnacle Point Cave 13B [4] highlighted the use of raw materials of different types and colours (e.g. mudstone, shale, siltstone, sandstone, iron oxide), and modifications produced by grinding, flaking, notching and, to a lesser degree, scraping. An engraved piece and a piece that shows marks indicating that it may have been suspended were also identified. Possible evidence for the heating of ochre, and a preference for dark red shades has been advanced in support of symbolic activities, such as body painting.

Several thousand ochre pieces were recovered from the MSA levels of Diepkloof Rockshelter dated to 110–55 ka. The analysis of 558 pieces (1.9 kg) [34,57] identified different types of rocks (shale, ferricrete, shale/ferricrete, ferruginous sandstone, ferruginous quartzite), with the presence of exogenous raw materials suggesting complex mobility patterns [58]. Modifications were identified on 16% of the analysed assemblage, including striations produced by grinding, and to a lesser extent, smoothed areas. A number of pieces appear to have been intentionally shaped, and one is engraved. Flaking is rare, and scraping apparently absent. Quartzite slabs and silcrete flakes bearing ochre residues may have been used as processing tools. According to Dayet et al. [58], the exogenous nature of the ochre, the selection of particular ochre types, and the absence of ochre in adhesives used for tool hafting [59] are consistent with the symbolic interpretation.

The MSA levels of Sibudu, South Africa, dated between 77.2 ± 2.6 ka– 37.6 ± 2.6 ka [60–63], yielded 5449 pieces of ochre (>8mm, 15,4 kg), as well as 3837 small pieces. Various raw materials [36] were identified by visual inspection (shale, siltstone, snuffbox shale, sandstone, iron oxide, hardened clay, mudstone, weathered dolerite). Microscopic observations [40], supported by experimental data [64], allowed the identification of a variety of modifications produced by grinding, rubbing and scoring on 682 pieces [40]. Most of the modified pieces concern bright red shale. Clayey ochre appears in higher frequencies in the lower levels and silty ochre in the upper levels. This is interpreted by Hodgskiss as a shift in ochre use over time [36]. A few pieces, mostly from layers dated to between 77–58 ka, are interpreted as engraved [65]. Although others bear facets and a pointed morphology, the author questions, on experimental grounds, their interpretation as “crayons” [66]. Cemented hearths with substantial ochre deposits in layers dated to ca. 58 ka have been described as receptacles for ochre powder or work surfaces [67]. Sandstone slabs and other lithic artefacts with yellow or red residues were also recovered from the site [26,27,68,69]. The presence of ochre residue on the striking platform of flakes suggests large ochre lumps to have been used as soft hammers [68]. Ochre mixed with a possible resin identified on stone tools has been advanced as support for the presence of hafting adhesives. Finally, the production of a compound composed of ochre and milk has been identified on residue adhering to a dolerite flake in layers dated to 49 ka BP [70].

### Archaeological context

**Fig 1:**
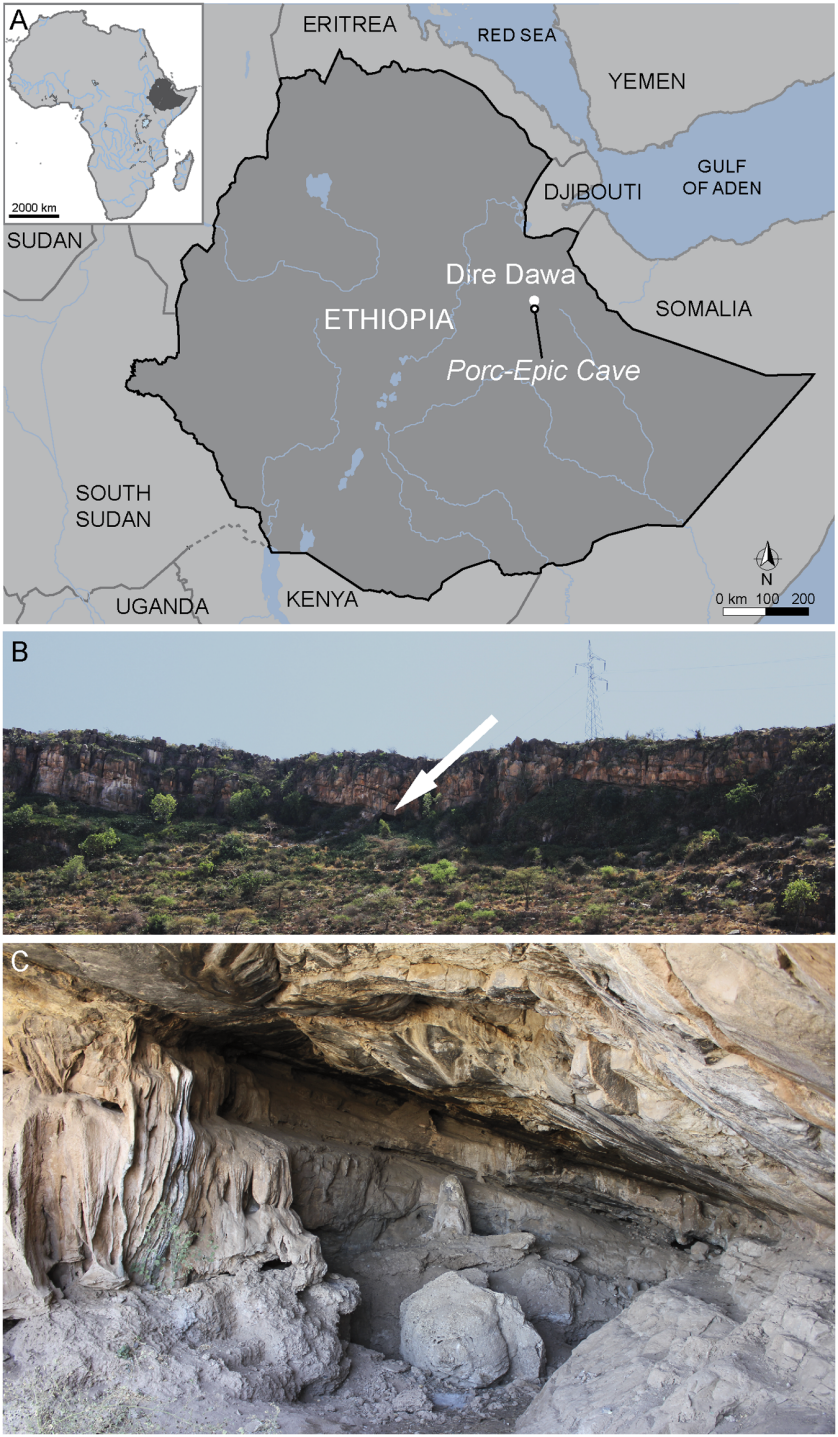
Location of Porc-Epic Cave. (A) Location of the site. (B) View of the cliff where the site is located. The arrow indicates the cave entrance. (C) View of the cave (photo A. Herrero). Modified after [38] under a CC BY license, with permission from PLOS ONE, original copyright 2016.

Porc-Epic Cave is located between the Afar Depression and the Somali Plateau, 3 km south of Dire Dawa in Ethiopia (Fig 1). The cave opens at the base of a Jurassic limestone cliff, 140 m above the wadi Laga Dächatu near the top of the Garad Erer hill.

Pierre Teilhard de Chardin and Henry de Monfreid discovered the cave in 1929, with a test pit conducted the same year to test the archaeological potential of the site [71]. The excavation was enlarged by Henri Breuil and Paul Wernert in 1933 [72]. Rock art of a "later schematic style" identified on the cave’s walls was also described [73,74]. New excavations directed by John Desmond Clark in 1974 [73,75] were followed in 1975–1976 by fieldwork led by Kenneth D. Williamson over an approximately 49 m^2^ surface. More recently, fieldwork conducted by a team from the *Muséum National d’Histoire Naturelle*, Paris, France, and the Authority for Research and Conservation of Cultural Heritage (ARCCH) of Ethiopia helped clarify the Porc-Epic stratigraphy [76].

**Fig 2:**
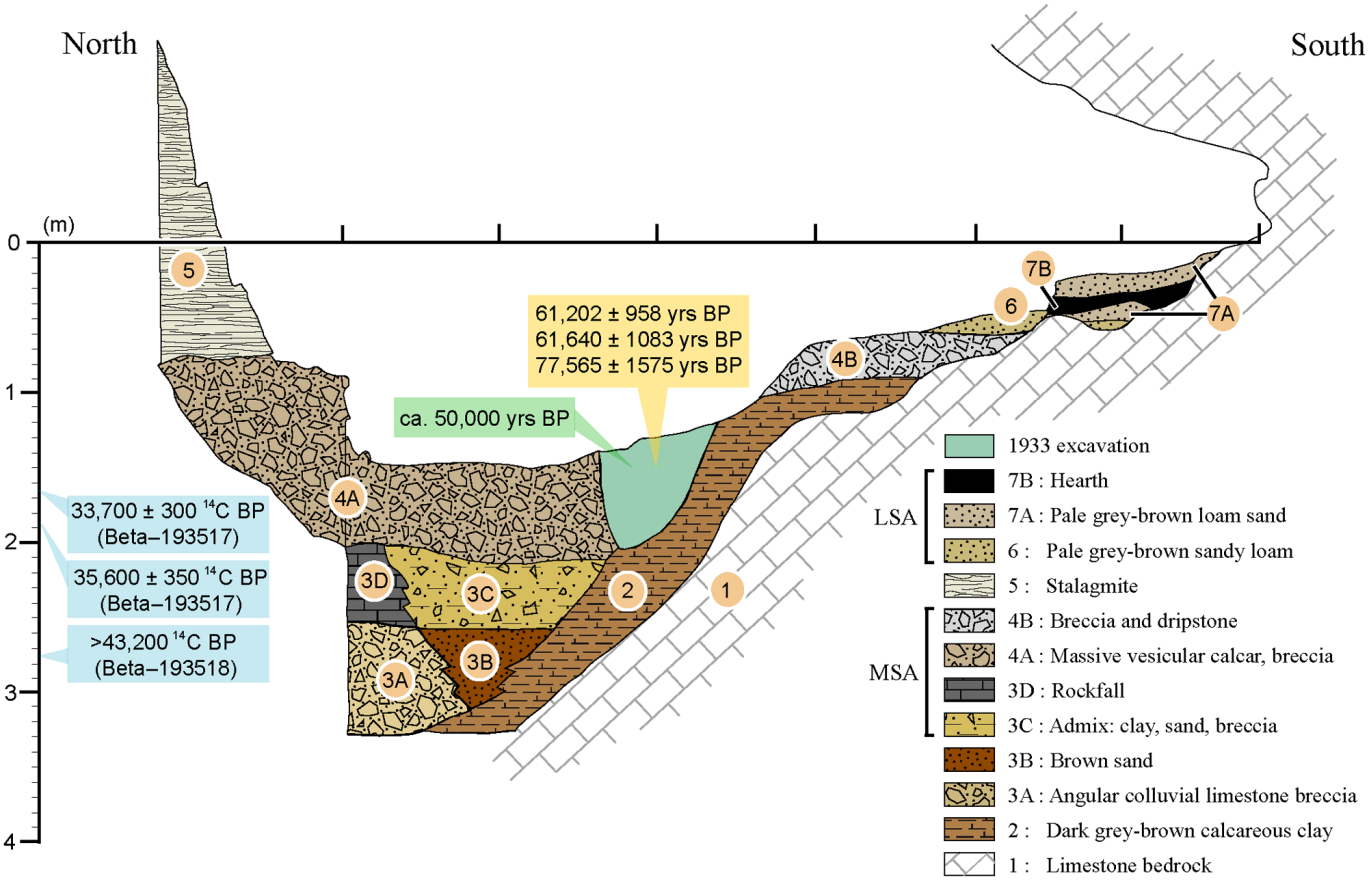
Porc-Epic Cave's stratigraphy. Eastern profile (09W-10W) at the end of the 1974 excavation. The gamma-spectrometry age of a human mandible and the obsidian hydration ages for artefacts recovered during the 1933 excavation are indicated in green and orange, respectively. ^14^C ages obtained from gastropod opercula are indicated in blue. Their stratigraphic position is approximate, as only the depth and square from which these objects were recovered is known, and therefore cannot be correlated with a specific layer. Reprinted from [38] under a CC BY license, with permission from PLOS ONE, original copyright 2016.

Divided into seven units (Fig 2), the stratigraphy comprises a succession of clayish levels, sandy levels and breccia (see [41,73] for details). MSA material was recovered from levels 2, 3C/D and 4A/B [73], approximately 60 to 220–230 cm below datum. Above these layers, layers 6, 7A and 7B are all composed of fine sands and loam with interstratified hearth material containing LSA and Neolithic artefacts [77].

Three artefacts found during the 1933 excavation [78] were dated by obsidian hydration to 61,202 ± 958, 61,640 ± 1083, and 77,565 ± 1575. However, this dating method is now considered unreliable [79,80] and, although the samples come from MSA levels, the exact stratigraphic provenance of these tools is unknown. High-resolution, low-background gamma-ray spectrometry analyses of a human mandible produced a date of ca. 50 ka [81]. Accelerator mass spectrometry (AMS) radiocarbon determinations for three samples of *Revoilia* gastropod opercula from the MSA layers [77] returned uncalibrated ^14^C ages of 33,700 ± 300 (Beta–193517), 35,600 ± 350 (Beta–193516), and >43,200 (Beta–193518). The 95.4% probability range of the two finite ages are 38,800–37,049 cal BP, and 41,084–39,421 cal BP (IntCal13; OxCal 4.2; [82]). The stalagmite that seals the breccia containing the main MSA levels yielded ^14^C and U–Th ages of, respectively, 4,590 ± 60 BP and 6,270 ± 1020 BP [73]. Charcoal fragments recovered in the uppermost breccia have been dated to 5,700 ± 110 BP. Overall, radiocarbon ages obtained from *Revoilia* opercula at Porc-Epic Cave seem to indicate the sequence to have accumulated over at least 4,500 years [41,83]. However, given the uncertainty surrounding ages obtained at the site, we cannot exclude that Porc-Epic’s sequence may be longer than suggested by radiocarbon ages.

At Porc-Epic Cave, the study of lithic artefacts showed that flakes, blades, bladelets and points were produced using Levallois, Discoid and laminar reduction methods by direct hard-hammer percussion [73,76,81,84–88]. Flint, basalt, obsidian [89,90] and sandstone/quartzite were the main raw materials exploited. Various interpretations have been advanced for the microliths from Porc-Epic Cave. Desmond Clark and Williamson suggested that their presence in the upper levels differentiates the LSA from MSA levels [73]. More recently, Pleurdeau [76,86–88] identified a small number of microliths and backed bladelets in the MSA assemblage, which he interpreted as a gradual evolution from the MSA to the LSA. It was later suggested, however, that the presence of microliths may be result either from mixing with the overlying LSA layers or the intense reduction of raw materials such as obsidian [81]. Several human cranial fragments and a partial mandible showing both modern and archaic features [91] were found at the site. The identification of different mammal taxa in the MSA levels suggests a nearby water source and widespread grasslands. The skeletal element profile reveals a selective transport of high-ranking nutritional elements, leading the site to be interpreted as a base camp [77]. Fossil ungulate enamel isotope data from teeth recovered in the MSA levels of Porc-Epic Cave was analysed to identify possible shifts in climatic and environmental conditions [92]. However, most taxa (for example, *Equus quagga/grevyi*, *Aepyceros melampus*, *Damaliscus lunatus*, *Syncerus caffer*) yielded low δ^13^C and high δ^18^C values, with little or no changes throughout the sequence, suggesting dry grass feeding and high aridity. Other taxa, such as *Phacochoerus* sp. yielded a greater variability in δ^13^C and δ^18^C values, but no significant changes were observed throughout the stratigraphy among mixed feeders [92]. The MSA levels yielded more than 419 perforated gastropod opercula belonging to the terrestrial species *Revoilia guillainopsis*. Given that their presence cannot be attributed to natural processes and despite the lack of visible anthropogenic modifications on the perforations, they have nevertheless been interpreted as possible evidence for symbolic behaviour [83].

### Ochre at Porc-Epic Cave

Ochre was reported at Porc-Epic Cave by Breuil, and later by Desmond Clark and Williamson [73,93]. The latter described 214 ochre pieces and one limestone grindstone recovered during the 1974 excavations. The ochre assemblage from the 1975–1976 excavations comprises 4213 pieces (ca. 40 kg) of red, brown and yellow iron-rich minerals [41], as well as 21 ochre processing tools and 2 ochre-stained artefacts [38]. Although the size of the mesh used during sieving is unknown, many ochre pieces are smaller than 1 cm, indicating a fairly exhaustive recovery (see below).

**Fig 3:**
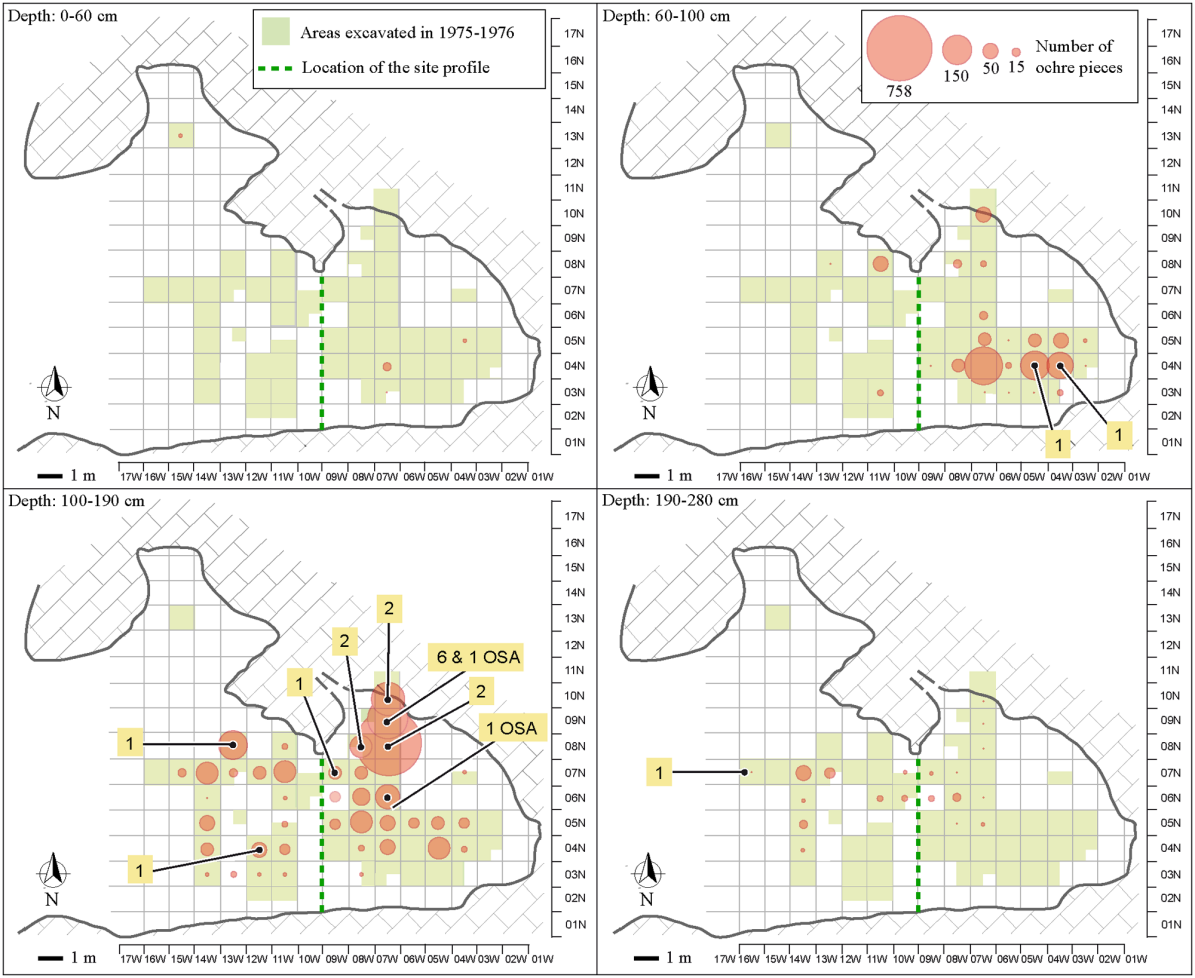
Spatial distribution of ochre pieces, ochre processing tools and ochre-stained artefacts. Bubble sizes reflect the frequency of ochre pieces per grid unit. Numbers indicate ochre processing tools and ochre-stained artefacts (OSA) when indicated. Modified after [38] under a CC BY license, with permission from PLOS ONE, original copyright 2016.

Ochre pieces are present between 30 and 280 cm below datum [41], with the highest frequency (83.15% of the total number of pieces) concentrated between 60–160 cm and peaking at 110–120 cm. The number of ochre pieces decreases gradually towards both the top and the bottom of the sequence. Analysis of the spatial and stratigraphic distribution identified two main ochre concentrations (Fig 3). The first (NEA) is located at 100 to 190 cm below datum in the northeastern squares (squares 08N-07W, 08N-08W, 09N-07W, 10N-07W) and accounts for 50.73% (n = 1373) of the ochre pieces present at this depth. Twelve ochre processing tools and one ochre-stained artefact were equally found in this area. The second concentration (SEA), accounting for 62.27% (n = 558) of the ochre pieces recovered within this depth, lies between 60 and 100 cm below datum in the southeastern area of the site (squares 04N-04W, 04N-05W and 04N-07W). Two processing tools were also recovered in the SEA. Concomitant changes in the location of ochre concentration areas and ochre processing tools suggest areas devoted to ochre processing to have shifted spatially over time. Additionally, by comparing the distribution of ochre pieces and gastropod opercula dated by ^14^C, ochre use at Porc-Epic appears to have begun around or before 45 cal kyr BP, becoming particularly intense at ca. 40 cal kyr BP [41].

The technological analysis of the upper and lower grindstones from Porc-Epic Cave [38] revealed different types of modifications associated with ochre residues. The analysis of ochre residues showed that different types of ferruginous rocks were processed for the production of ochre powders of different coarseness and shades. It has been proposed that these powders probably met a variety of utilitarian and/or symbolic needs. A round pebble with half its surface covered with ochre and no use-wear related to ochre processing seems to have been dipped in a liquid medium. Possibly used as a stamp to create patterns or apply pigment to soft materials, this object supports the hypothesis that ochre was involved at Porc-Epic Cave in symbolic practices.

## Material and methods

The material analysed here includes all ochre pieces recovered at Porc-Epic Cave during Williamson's excavation (1975–1976). Currently housed in a permanent repository at the National Museum of Ethiopia in Addis Ababa, the collection comprises 4213 ochre pieces (39.97 kg, specimen numbers Porc-Epic ochre n. 2–3965) studied by one of us (DR) between 2011 and 2014. A permit to study the archaeological material and to export it temporarily was granted by the Authority for Research and Conservation of Cultural Heritages of Ethiopia (ARCCH). Around 10% of the pieces (n = 421) lack contextual information and were excluded from the analysis.

A number of contextual, technological and morphometric variables were recorded for each of the 3792 analysed pieces, including the square and 10 cm spit in which the object was found, the length, width, thickness and weight of complete objects (n = 3659), raw material and colour. Raw materials were identified based on their colour, texture, inclusions, hardness and density (Table 1). These visual criteria likely coincide with those considered by Middle Stone Age groups when selecting ochre. When possible, we recorded the original morphology of the piece (small slab, pebble, nodule, irregular). Streak or hardness analyses were not conducted in order to avoid damaging the archaeological specimens. Colour was characterised by visual inspection, and hardness determination was based on pulverulence and hand staining while manipulating the pieces. Light grey pieces were characterised as ochre when they showed red microscopic grains, and ambiguous pieces were not taken into account.

**Table 1:** Criteria for the determination of ochre types.

Raw material type	Colour	Texture		Inclusions	Hardness	Density
**Soft fine-grained (SFG)**	G, Y, BR, BL, O, R, DR	VF	Hom	None or few	Soft to hard	Light
**Banded fine-grained (BFG)**	Layers of Y, O, R, DR, G, BR	VF	Hom	None or few	Soft to hard	Light
**Hard fine-grained (HFG)**	G, Y, BL, O, R, DR, BR	VF	Hom	None	Very hard	Heavy
**Coarse-grained (CG)**	G, Y, BR, O, R, DR	C	Het	Subcirc / irreg	Soft to hard	Normal
**Ferruginous sandstone (FS)**	G, Y, BR, O, R, DR	C	Hom	Subcirc / irreg	Soft to hard	Normal
**Platy fine-grained (PFG)**	G, R	F + C	Het	Platelets	Soft	Light

G: grey; Y: yellow; BR: brown; BL: black; O: orange; R: red; DR: dark red; VF: very fine; C: coarse; F: fine; Hom: homogeneous; Het: heterogeneous; subcirc: subcircular; irreg: irregular.

**Fig 4:**
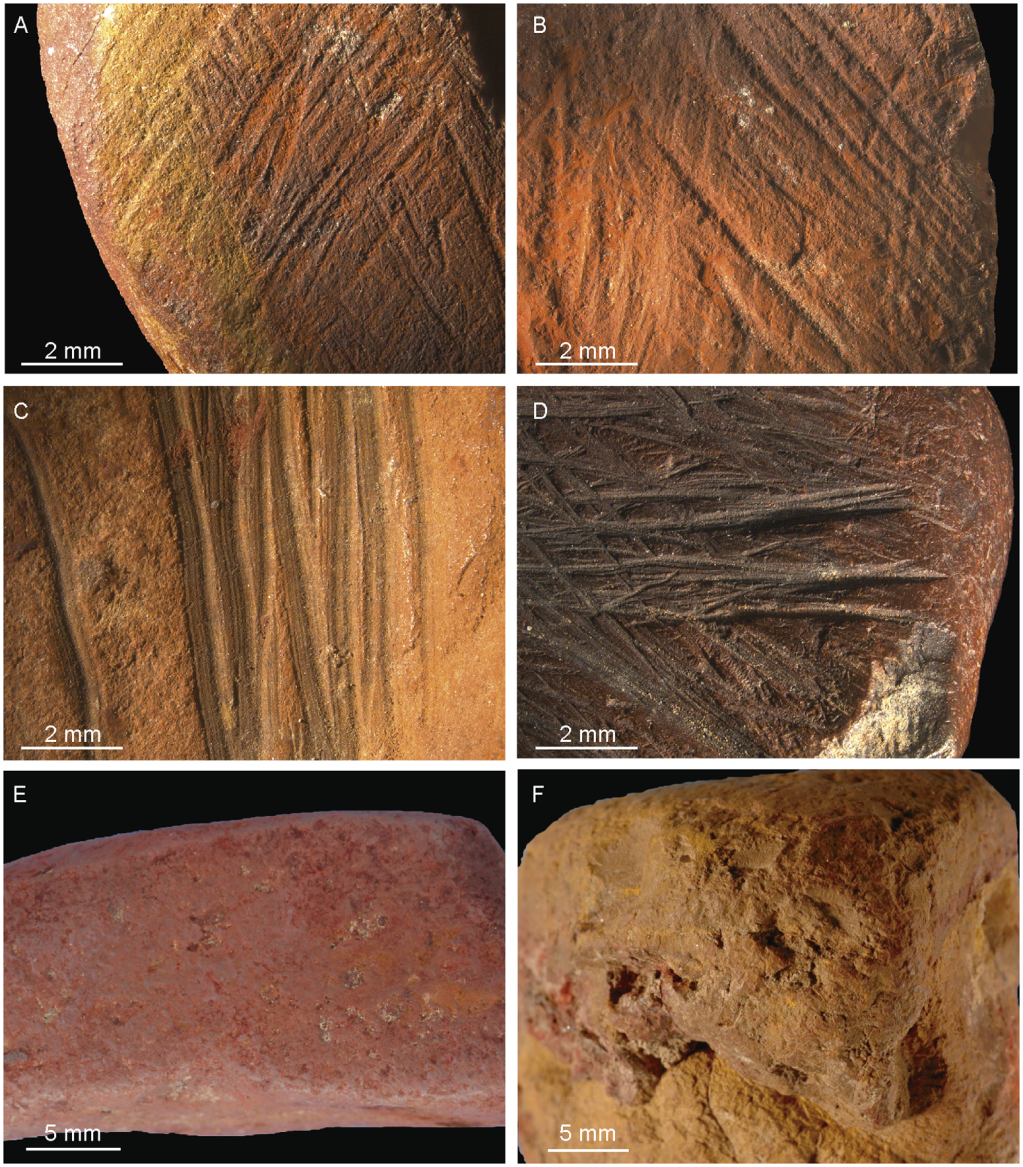
Modifications on ochre pieces. (A, B) Striations produced by grinding PE102 and PE987. (C, D) Incisions produced by scraping/scoring PE306 and PE1419. (E) Smoothed areas on PE3067. (F) Pits produced by a pounding action on PE931 (OPT21).

Anthropogenic modifications were identified macro- and microscopically, and photographed with a Leica Z6 APO macroscope (Fig 4). We recorded traces of flaking, striations, facets, smoothed areas, incisions, and pits. Pieces bearing traces of flaking include objects with simple or multiple flake scars and flakes. Striations (Fig 4A and 4B) produced by grinding the piece against an abrasive surface are present as linear parallel marks arranged in groups [21,64,65]. Facets refer to areas flattened by grinding and covered with striations. Facet size and cross-section (convex, flat, concave) were recorded in addition to the orientation of the striations with respect to the facet lengths. Incisions (Fig 4C and 4D) are present as sub-parallel, slightly curved marks displaying multiple grooves (or micro-striations defined as microscopically visible parallel striations) produced by the asperities of lithic cutting edges or other sharp tools during scraping or scoring [21,40,64]. Smoothed areas (Fig 4E) refer to homogeneous surfaces lacking irregularities and projections in comparison to neighbouring unmodified areas or those on which modification marks have been partially or fully erased [64]. Percussion pits (Fig 4F) take the form of depressions produced by a pounding action [38,94,95].

### Experimental and ethnographic data

**Fig 5:**
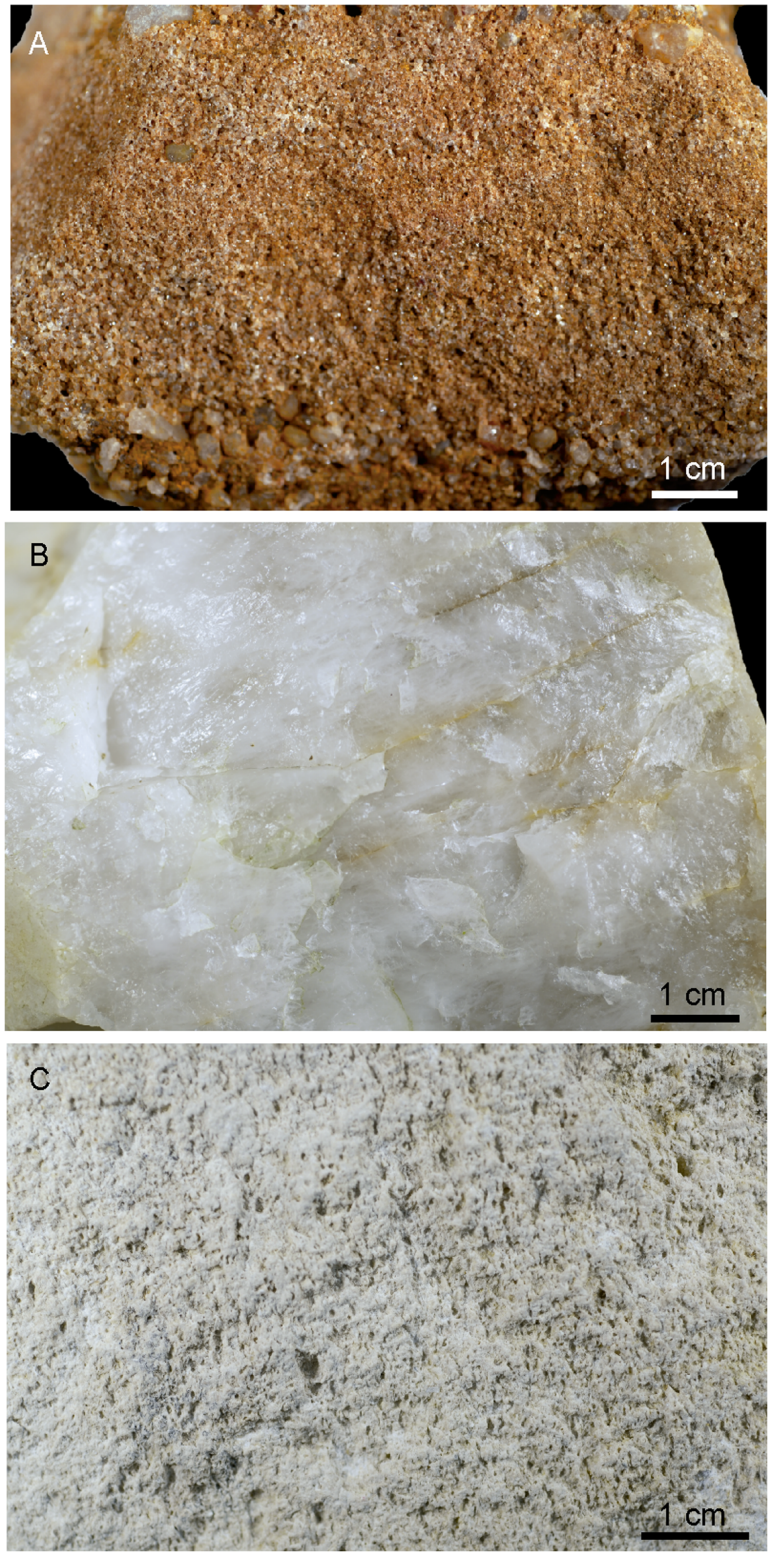
Experimental grindstones. Experimental grindstones G1 (A), G2 (B) and G3 (C).

Three ochre pieces were ground on three different grindstones, applying the same pressure until a distinct facet was produced. Grindstones were made of sandstone (G1, Fig 5A), quartzite (G2, Fig 5B), and limestone (G3, Fig 5C), which are among the rock types used at Porc-Epic Cave for grinding ochre [38]. The sandstone grindstone (G1) and ochre pieces used in the experiments (EXP1, EXP2, EXP3) were collected from the wadi Laga Dächatu and are comparable to ochre types found at the site (SFG, CG—see below). EXP1 is made of a soft, clayish, fine-grained homogeneous ferruginous rock. EXP2 and EXP3 are slightly harder. The first is more heterogeneous and contains a few well cemented coarse grains. The second is highly heterogeneous and rich in well cemented coarse grains.

The nine powder samples produced during the experiments were kept in separate sampling tubes and submitted to granulometric analyses described below. Two samples of ochre powder produced by Hamar women [96–98] to coat their hair and six samples of ochre ground by Ovahimba women to cover their body, hair and attire were also submitted to granulometric analyses. Both Ovahimba and Hamar women produce pigment powder by crushing ochre lumps with upper grindstones and grinding the resulting fragments between upper and lower grindstones. The Hamar samples were collected by one of us (DR) in Turmi and Dombo, Southern Ethiopia. Results of the analysis of Ovahimba samples, collected during fieldwork conducted by one of us (FD), have been presented elsewhere [30].

### Surface texture analysis

Different surface textures result from material loss due to abrasion [99]. This is the case with facets created by grinding ochre pieces on grindstones. Confocal microscopy allowed the surface topography of experimental and archaeological facets to be quantitatively compared in order to explore whether the type of grindstone can be identified.

Rugosimetric analysis was conducted with a Sensofar S-Neox confocal microscope driven by the SensoScan 6 software (Sensofar, Barcelona) on facets present on nineteen SFG (see below) ochre pieces from Porc-Epic Cave and nine facets produced experimentally. The archaeological ochre pieces vary in texture and show features intermediate between EXP1 and EXP2. Depending on the facet surface, one to three 4 x 3 mm^2^ areas were captured per facet. We used a 20x objective (N.A. 0.45) with green light illumination and a measurement step of 1 μm. These parameters allow for a spatial sampling of 0.65 μm and a vertical resolution of 8 nm. Only surfaces with more than 95% of measured points were retained for analysis.

Data was processed with SensoMap 7.3. Form was removed by subtracting a second-degree polynomial, and isolated or around edge outliers were removed and non-measured points filled. A Gaussian filter was applied to these areas to separate roughness and waviness with a 0.25 mm cut-off value, and captured areas were subsequently divided into four 2 x 1.5 mm^2^ sub-areas. ISO 25178 international standards were used to calculate different 3D area surface texture parameters for roughness [100–102]. We selected one height parameter (Sq: Root Mean Square Roughness, i.e. standard deviation of the height distribution) for further analysis and one hybrid parameter (Sdr: developed interfacial area ratio, i.e. the increase in surface when flattening the measured area). Sq quantifies the statistical distribution of height values around the mean plane and Sdr the complexity of the surface [103].

### Particle-size analysis

A Horiba LA950 laser scattering particle sizer was used to analyse experimental and ethnographic ochre powder. *Mie* solution to Maxwell’s equations [104], which provides the basis for measuring the size of particles through the scattering of electromagnetic radiation, was used to calculate the particle size distribution in aqueous solution (refractive index 1.333). Calculations were made with the refractive index of hematite (2.94i–0.01i). The pre-treatment of samples included suspension in sodium hexametaphosphate (5 g/L) for 12 hours and 60 seconds of ultrasonification to achieve optimal dispersion.

## Results

### Raw material types and colour

**Fig 6:**
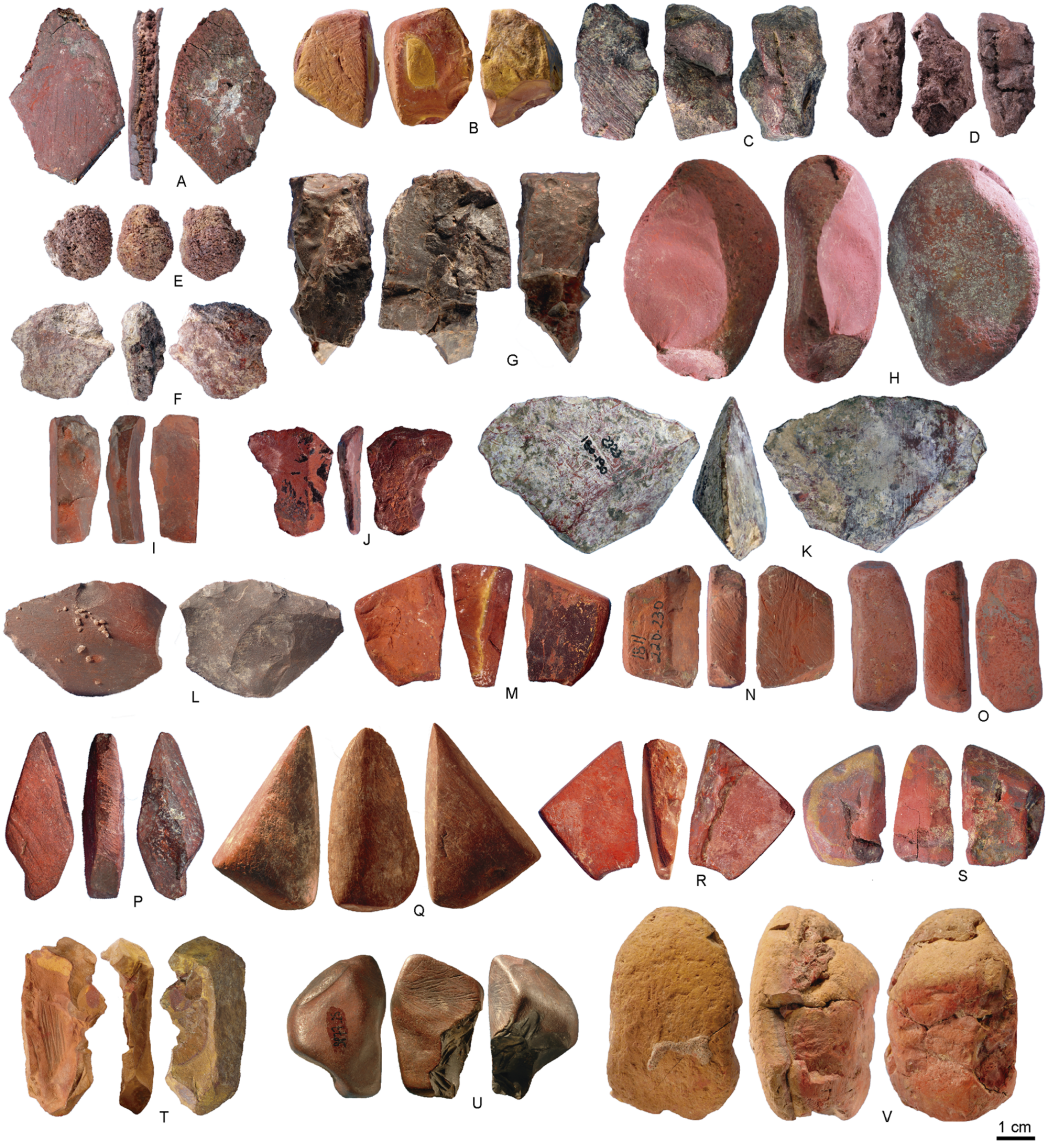
Ochre pieces from Porc-Epic Cave. (A) Ochre piece PE1699, SFG. (B) Ochre piece PE2104, BFG. (C) Ochre piece PE1752, HFG. (D) Ochre piece PE436, PFG. (E) Ochre piece PE1577, FS. (F) Ochre piece PE809, CG. (G) Ochre piece PE962, HFG. (H) Ochre piece PE2563, SFG. (I) Ochre piece PE420, SFG. (J) Ochre piece PE2063, SFG. (K) Ochre piece PE3358, SFG. (L) Ochre piece PE312, SFG. (M) Ochre piece PE1806, BFG. (N) Ochre piece PE987, SFG. (O) Ochre piece PE3067, SFG. (P) Ochre piece PE1862, SFG. (Q) Ochre piece PE1677, SFG. (R) Ochre piece PE1493, SFG. (S) Ochre piece PE102, BFG. (T) Ochre piece PE306, BFG. (U) Ochre piece PE1419, HFG. (V) Ochre piece PE931, OPT21, BFG.

**Fig 7:**
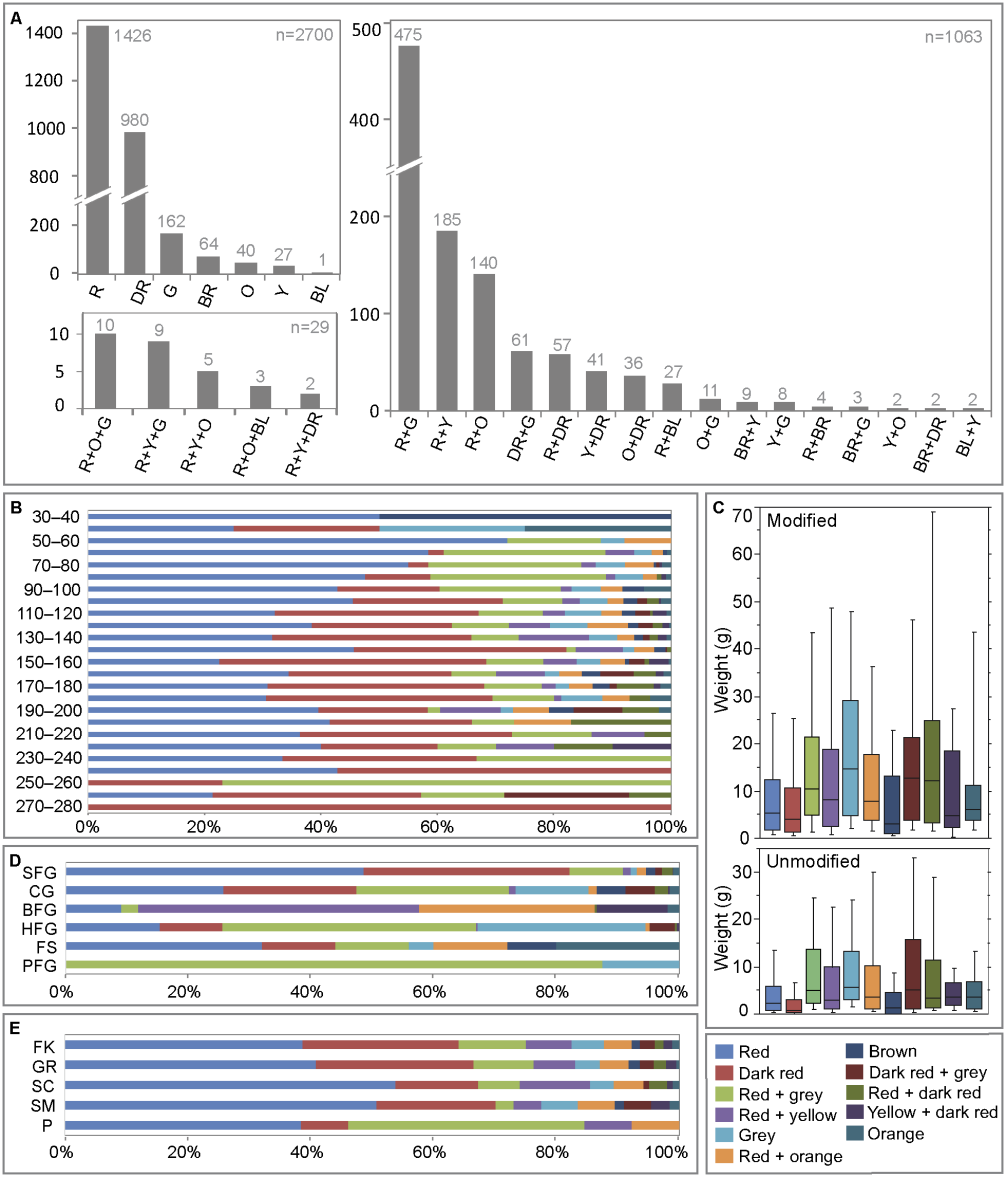
Colours of ochre pieces from Porc-Epic Cave. (A) Grey numbers represent the number of pieces. Colours per ochre piece are represented in separate histograms. R: red. DR: dark red; G: grey; BR: brown; O: orange; Y: yellow; BL: black. (B) Vertical distribution of main colours and colour associations on ochre pieces. Data is presented in percentages for 10 cm spits. (C) Main colours and colour associations on modified and unmodified ochre pieces by weight. (D) Main colours and colour associations on ochre pieces by raw material type. (E) Main colours and colour associations on ochre pieces by modification type.

The ochre pieces display a variety of colours and shades (Fig 6, Figs A–E in S1 Figs). Three quarters (n = 2700; 71%) have a single colour, followed by smaller numbers with either two (n = 1063) or three (n = 29) (Fig 7, Tables A–E in S1 Tables). While yellow, orange, red, dark red, brown, grey and black shades were identified, red and dark red shades are most common, accounting, respectively, for 37.6% and 26% of the total number of pieces. Pieces combining either red and grey (12.5%) or red and yellow (4.88%) are also relatively frequent.

Due to the high degree of fragmentation and reduction intensity, it was possible to determine the original morphology of pieces for only 29% of the pieces (Table 2). Ochre was most frequently imported to the site in the form of slabs (n = 401, Fig 6A), followed by irregular pieces (n = 265, Fig 6C, 6D and 6G), nodules (n = 260, Fig 6B) and pebbles (n = 166, Fig 6H).

**Table 2:** Morphology of unmodified and modified ochre pieces.

Morphology	Unmod	% Unmod	Mod	% Mod	TOTAL	% TOTAL
**Undetermined**	1426	69.46	1274	73.26	2700	71.20
**Slab**	263	12.81	138	7.94	401	10.57
**Pebble**	64	3.12	102	5.87	166	4.38
**Nodule**	115	5.60	145	8.34	260	6.86
**Irregular**	185	9.01	80	4.60	265	6.99
**Total**	2053	100	1739	100	3792	100

Number and percentages of ochre pieces. Unmod: unmodified; Mod: modified.

**Fig 8:**
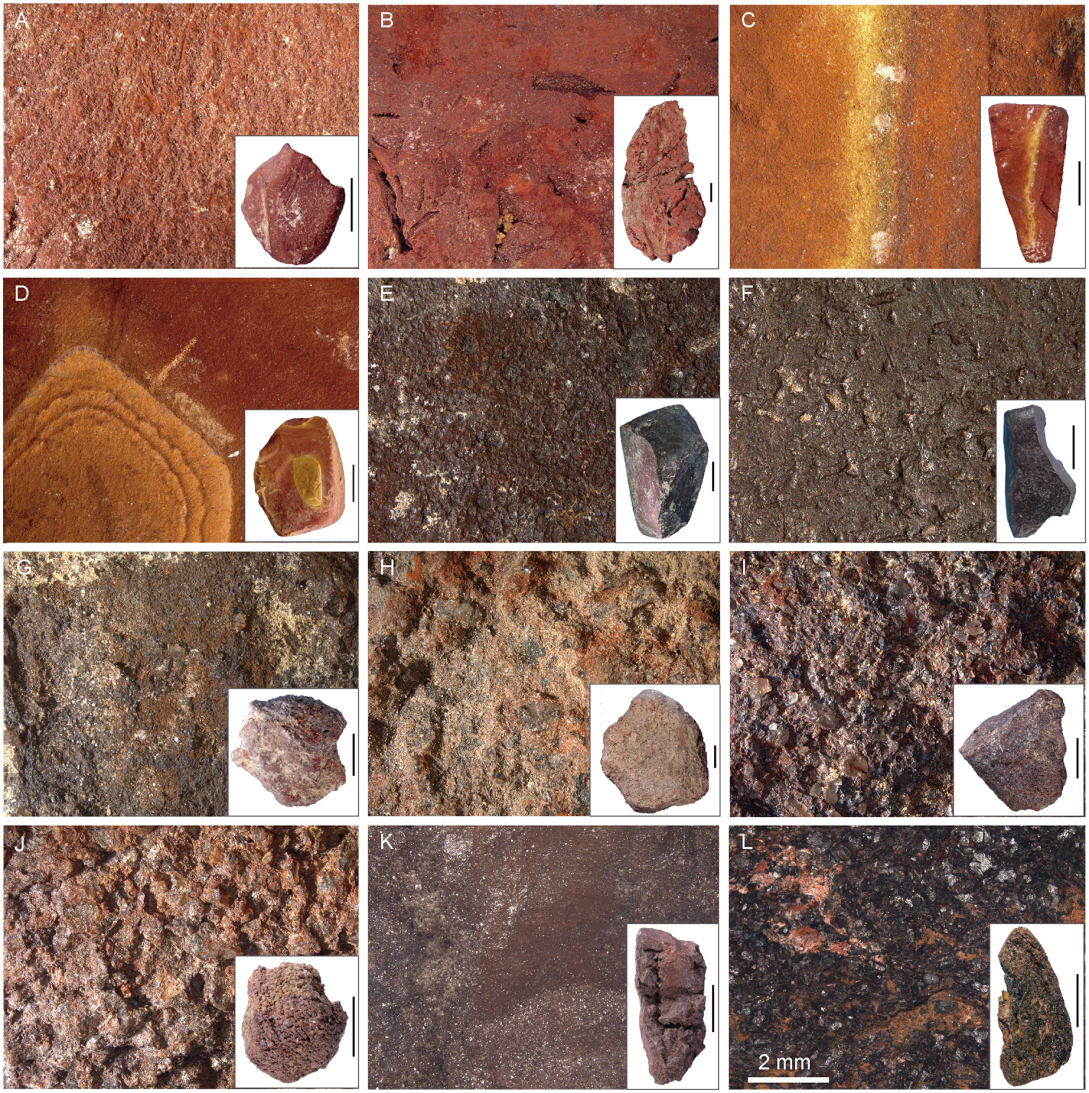
Ochre raw material types. (A, B) Soft fine-grained (SFG): PE725 and PE1942. (C, D) Banded fine-grained (BFG): PE1806 and PE2104. (E, F) Hard fine-grained (HFG): PE1734 and PE2282. (G, H) Coarse-grained (CG): PE809 and PE1666. (I; J) Ferruginous sandstone (FS): PE965 and PE1577. (K, L) Platy fine-grained (PFG): PE436 and PE1812. Scales of overall photos of the pieces = 1 cm.

Six types of raw material were identified (Fig 8, Table 1).

- Soft, fine-grained ferruginous rocks (SFG, Fig 8A and 8B): homogeneous, fine-grained, clayish rocks with very few or no inclusions that are mostly red and dark red, but also grey, brown, orange or yellow in colour and only rarely bear small black spots (Fig 7D). Most are slabs (n = 339) or irregular pieces (n = 218), although nodules (n = 117) and pebbles (n = 107) are also observed. Several examples have a compact structure; others are laminated or show small cavities. These raw materials are generally light.
- Banded, fine-grained ferruginous rocks (BFG, Fig 8C and 8D): rocks with the same texture and appearance as type SFG but with clearly differentiated layers of colours, that are mostly red and yellow, or red and orange but also dark red, grey or brown (Fig 7D). These rocks are mostly found as nodules (n = 90), followed by smaller numbers of slabs (n = 23) and irregular pieces (n = 19) or pebbles (n = 14). Generally light, these materials usually have a compact structure.
- Hard, fine-grained ferruginous rocks (HFG, Fig 8E and 8F): very hard and heavy iron oxides characterised by dark colours, mostly grey and red, but also black. They rarely show brown, yellow, dark red, grey and orange spots (Fig 7D). These materials usually occur as nodules (n = 51) and pebbles (n = 45) and more rarely as slabs (n = 33) and irregular pieces (n = 24).
- Coarse-grained ferruginous rocks (CG, Fig 8G and 8H): heterogeneous agglomerates of mostly red, dark red and grey grains, and more rarely yellow, orange, and brown grains (Fig 7D). They are generally irregular in shape (n = 49) or occur in the form of nodules (n = 42) and, to a lesser degree, pebbles (n = 25) or slabs (n = 28).
- Ferruginous sandstone (FS, Fig 8I and 8J): agglomerates of translucent grains (probably quartz) in a fine iron oxide matrix. They are mostly red and orange, sometimes with dark red, grey, brown and yellow spots (Fig 7D). Although commonly found as slabs (n = 5), nodules (n = 2) or pieces with irregular morphologies (n = 2) were also recorded.
- Platy fine-grained ferruginous rocks (PFG, Fig 8K and 8L): agglomerates of platelets (probably micas) characterised by a shiny or metallic-like appearance. They are usually greyish with red veins (Fig 7D), and can be irregular (n = 2) or flat in shape (n = 1).

SFG is the most frequent type (68.2%, n = 2588), followed by CG (12.8%, n = 486), BFG (9.78%, n = 371) and HFG (8.14%, n = 309). The FS and PFG are rare (n = 30 and 8), accounting, respectively, for only 0.79% and 0.21% of the assemblage (Table F in S1 Tables).

### Raw material and colour changes through time

**Fig 9:**
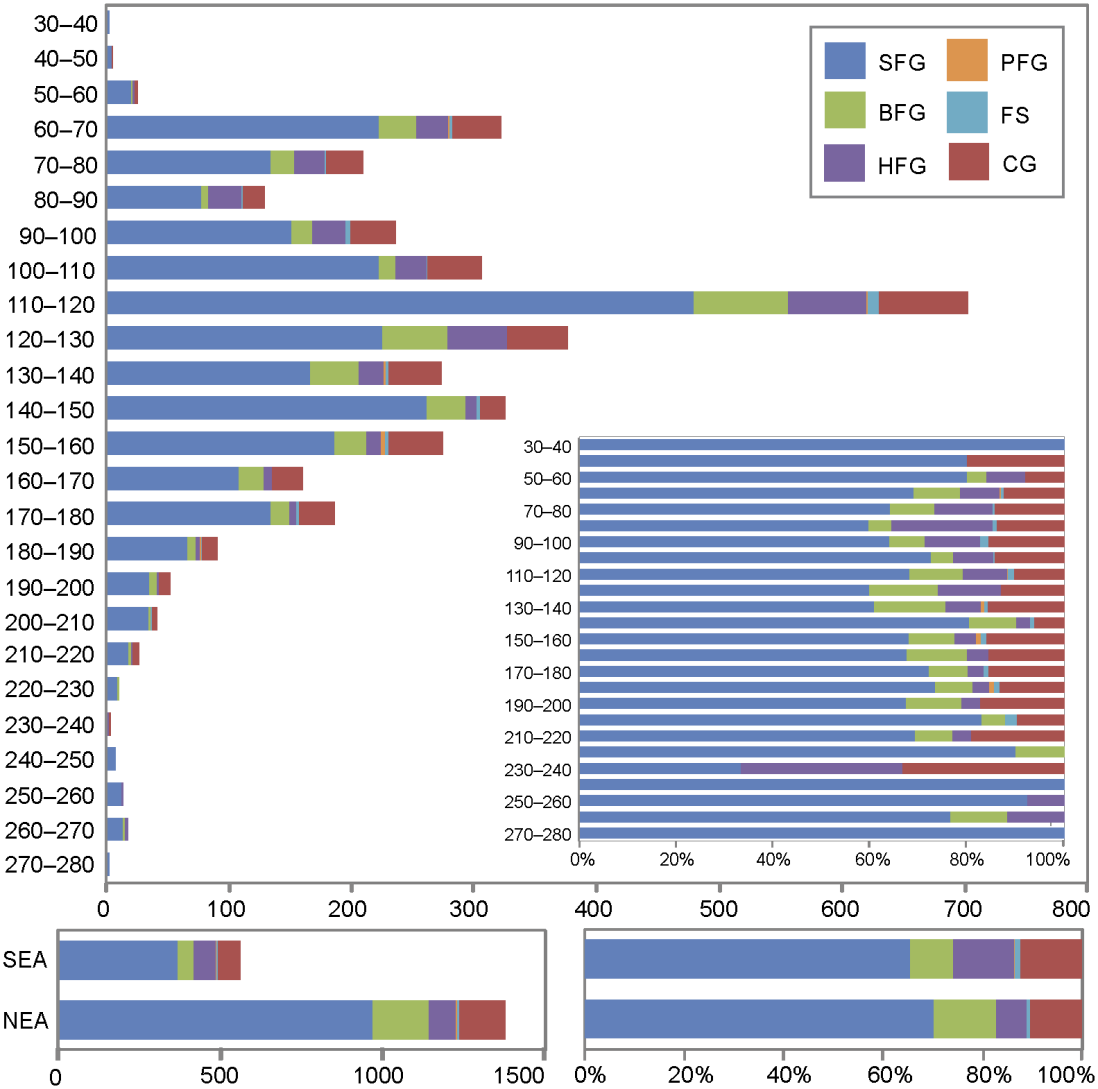
Vertical distribution of ochre pieces per raw material type. Data is presented in number of pieces and percentages. Separate histograms are presented for ochre from the northeastern (NEA) and southeastern (SEA) accumulations. SFG: Soft fine-grained; BFG: banded fine-grained; HFG: hard fine-grained; PFG: platy fine-grained; FS: ferruginous sandstone; CG: coarse-grained.

The proportion of the six raw materials remains relatively stable throughout the sequence (Fig 9, Table F in S1 Tables). Variations observed at the top (-30–60 cm) and bottom (-210–280 cm) of the sequence are not substantial due to small sample size. More than half of the ochre pieces in all levels are of the SFG type; the proportion of type CG oscillates between 10% and 15%, and types BFG and HFG range between 5% and 15%, and 3% and 20%, respectively. Type FS is systematically present, but in very low proportions. Type PFG is only recorded sporadically in levels in which ochre is abundant. The only noticeable change concerns type HFG, which is more abundant at depths between -60–140 cm. HFG type increases consistently from 140 to 80 cm and then declines slightly from 80 to 60 cm. The two areas with concentrations of ochre and ochre processing tools (Fig 3) follow the same pattern of the levels in which they occur. By weight (Tables G and H in S1 Tables), the proportion of each raw material does not differ substantially from what we described above for their numbers. SFG is still the most frequent raw material type, oscillating between 76% and 40% per level, followed by CG (8–42%). BFG and HFG range between 3% and 20% and 5% and 27% respectively. FS and PFG are still present in low proportions.

Interesting differences can be seen in the proportion of ochre colours over time (Fig 7B, Tables A and B in S1 Tables). Although red and dark red shades are dominant in all levels, they become proportionally less well represented in levels in which ochre is more abundant (-60–160). These levels are richer in grey, brown, orange, and yellow pieces and pieces of multiple colours. We also observe a decline in dark red, and red+yellow, and an increase in red, and red+grey between 100 cm and 60 cm.

**Fig 10:**
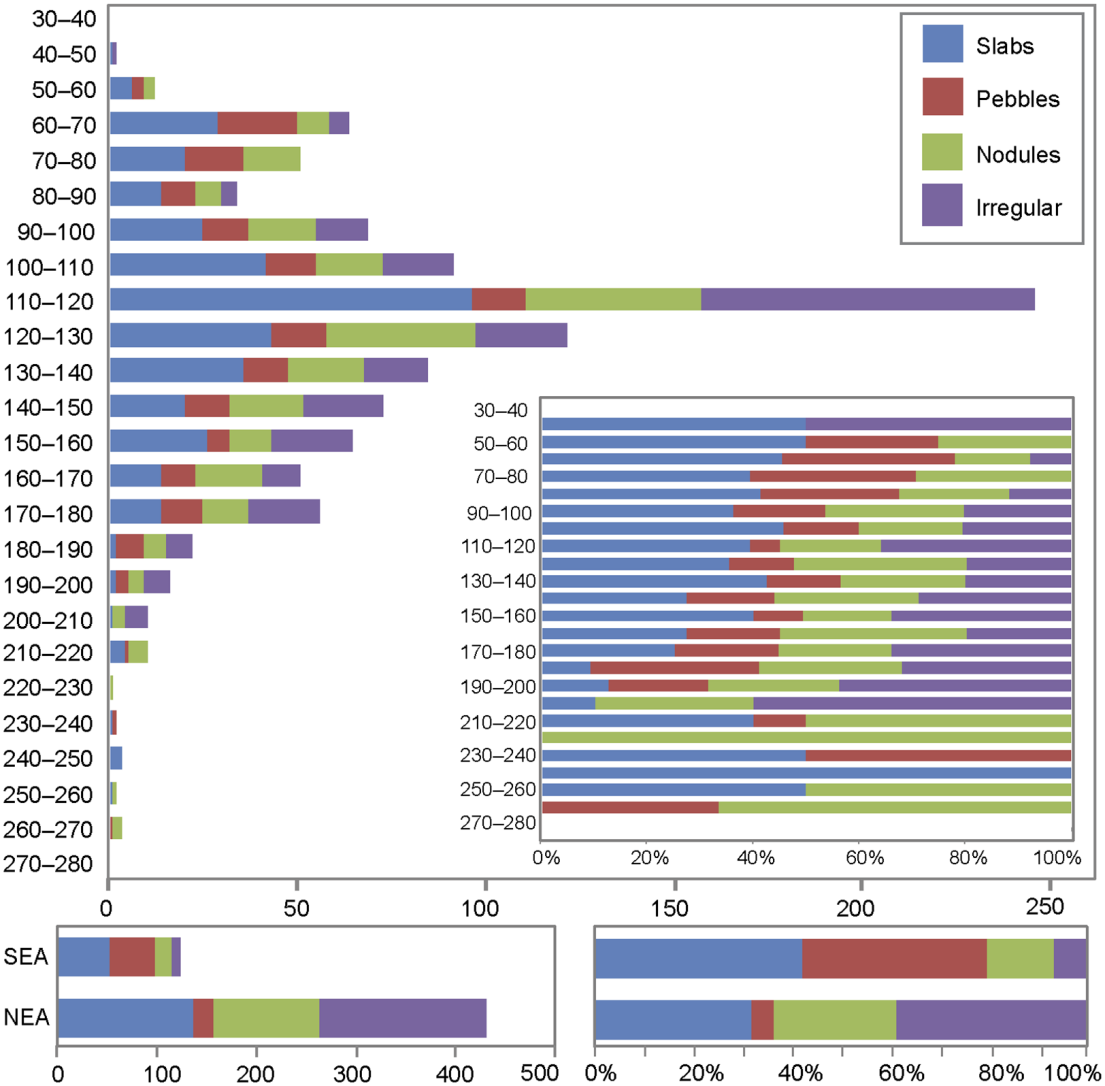
Vertical distribution of ochre piece morphology. Data is presented in number of pieces and percentages. Pieces with undetermined morphologies were not included. Separate histograms are presented for ochre pieces from the northeastern (NEA) and southeastern (SEA) accumulations.

Diachronic changes are also observable in terms of piece morphology. Slabs gradually increase in proportion towards the upper levels while irregular pieces follow the opposite trend (Fig 10). The same pattern can be seen with the two ochre concentrations (Figs 3 and 10).

### Technological analysis

#### Traces of modification

Almost half (n = 1739; 45.8%) of the 3792 analysed ochre pieces bear anthropogenic modifications (Table I in S1 Tables), accounting for 63.2% (25.3 kg) of the total weight of the analysed assemblage (Table J in S1 Tables).

Traces of flaking are most frequent (Table 3, Table K in S1 Tables), recorded on 1242 (71.4%) of the modified pieces or 45.1% (18 kg) of the total ochre analysed, in the form of flake scars (n = 1006, 57.8%, Fig 6H, Fig A in S1 Figs) and flakes (n = 236, 13.5%, Fig 6J, Fig A in S1 Figs). The latter include 17 bladelets (Fig 6I, Fig A in S1 Figs) and a number of retouched pieces, two of which resemble transverse scrapers (Fig 6K and 6L, Fig A in S1 Figs). Fourteen pieces bearing multiple, adjacent flake scars were clearly used as cores to produce ochre flakes. Lumps with traces of grinding (n = 913, 52.5%; 14.3 kg, 35.8% of the total ochre analysed; Fig 6B, 6C and 6L–6S, Figs B and C in S1 Figs) comprise either pieces with clear facets (n = 821, 42.2%) or areas covered by parallel striations that do not flatten the surface (n = 92, 5.2%). A relatively small proportion of pieces (n = 161, 17.6%) bear traces of grinding over more than 50% of their surface, a third (n = 334, 36.6%) half of their surface, and almost half (n = 418, 45.7%) less than 50% of their surface.

**Table 3:** Number of modified ochre pieces and weight per modification type and raw material.

MODIFICATIONS	OCHRE TYPES						Total (n)	OCHRE TYPES (kg)						Total (kg)
	SFG	CG	BFG	HFG	FS	PFG		SFG	CG	BFG	HFG	FS	PFG	
**FLAKING**	**827**	**96**	**163**	**151**	**4**	**1**	**1242**	**8.143475**	**4.18393**	**2.16384**	**3.45431**	**0.04963**	**0.01223**	**18.01**
*Flake scars*	*641*	*84*	*136*	*141*	*3*	*1*	*1006*	*7*.*34798*	*4*.*11129*	*2*.*04161*	*3*.*25587*	*0*.*04886*	*0*.*01223*	*16*.*82*
*Flakes*	*186*	*12*	*27*	*10*	*1*	*0*	*236*	*0*.*795495*	*0*.*07264*	*0*.*12223*	*0*.*19844*	*0*.*00077*	*0*	*1*.*19*
**GRINDING**	**635**	**73**	**109**	**90**	**4**	**2**	**913**	**7.93489**	**2.61248**	**1.54861**	**2.20386**	**0.04481**	**0.02137**	**14.37**
*1 facet*	*293*	*34*	*60*	*37*	*1*	*1*	*426*	*2*.*59426*	*0*.*74396*	*0*.*68325*	*0*.*62087*	*0*.*01535*	*0*.*01264*	*4*.*67*
*2 facets*	*141*	*16*	*17*	*15*	*1*	*0*	*190*	*2*.*19612*	*0*.*65687*	*0*.*31271*	*0*.*34047*	*0*.*00291*	*0*	*3*.*51*
*3 facets*	*78*	*8*	*5*	*9*	*0*	*1*	*101*	*0*.*74727*	*0*.*54402*	*0*.*12732*	*0*.*17557*	*0*	*0*.*00873*	*1*.*60*
*4 facets*	*20*	*5*	*6*	*6*	*1*	*0*	*38*	*0*.*44685*	*0*.*40329*	*0*.*08857*	*0*.*24139*	*0*.*01682*	*0*	*1*.*20*
*5 facets*	*19*	*1*	*6*	*4*	*0*	*0*	*30*	*0*.*43208*	*0*.*0092*	*0*.*09371*	*0*.*11525*	*0*	*0*	*0*.*65*
*6 facets*	*12*	*1*	*1*	*5*	*0*	*0*	*19*	*0*.*42509*	*0*.*00425*	*0*.*01876*	*0*.*45547*	*0*	*0*	*0*.*90*
*7 facets*	*8*	*1*	*1*	*1*	*0*	*0*	*11*	*0*.*11299*	*0*.*07018*	*0*.*02788*	*0*.*01169*	*0*	*0*	*0*.*22*
*8 facets*	*1*	*0*	*1*	*0*	*0*	*0*	*2*	*0*.*00533*	*0*	*0*.*01836*	*0*	*0*	*0*	*0*.*02*
*9 facets*	*1*	*0*	*1*	*0*	*0*	*0*	*2*	*0*.*02168*	*0*	*0*.*02334*	*0*	*0*	*0*	*0*.*05*
*11 facets*	*0*	*0*	*0*	*1*	*0*	*0*	*1*	*0*	*0*	*0*	*0*.*0231*	*0*	*0*	*0*.*02*
*18 facets*	*0*	*0*	*1*	*0*	*0*	*0*	*1*	*0*	*0*	*0*.*01361*	*0*	*0*	*0*	*0*.*01*
**SCRAPING**	**76**	**8**	**21**	**6**	**0**	**0**	**111**	**1.27936**	**0.77734**	**0.31793**	**0.57263**	**0**	**0**	**2.95**
**SMOOTHING**	**41**	**19**	**7**	**4**	**0**	**0**	**71**	**0.91099**	**1.05784**	**0.16851**	**0.10756**	**0**	**0**	**2.24**
**PITTING**	**8**	**4**	**1**	**1**	**0**	**0**	**14**	**0.62155**	**0.71545**	**0.11146**	**0.3645**	**0**	**0**	**1.81**
*1 end*	*6*	*1*	*0*	*0*	*0*	*0*	*7*	*0*.*20615*	*0*.*07316*	*0*	*0*	*0*	*0*	*0*.*28*
*2 ends*	*2*	*1*	*1*	*1*	*0*	*0*	*5*	*0*.*4154*	*0*.*27947*	*0*.*11146*	*0*.*3645*	*0*	*0*	*1*.*17*
*Entire surface*	*0*	*2*	*0*	*0*	*0*	*0*	*2*	*0*	*0*.*36282*	*0*	*0*	*0*	*0*	*0*.*36*

SFG: Soft fine-grained; CG: coarse-grained; BFG: banded fine-grained; HFG: hard fine-grained; FS: ferruginous sandstone; PFG: platy fine-grained. Notice that each modification type is considered independently. The total for pieces with ground facets is less than the total of ground pieces because some show striations due to grinding that was not intensive enough to create a flat surface.

While the number of facets on ground ochre varies between 1 and 18, more than half only have one facet (51.8%, n = 426). Pieces with two of more facets gradually decrease in proportion, with only six examples having 8 or more facets (Table 3). In cases of pieces with more than one facet (n = 395), facets are usually adjacent (n = 343, 86.8%). Facets on distinct areas are rare (n = 34, 8.6%) and very few pieces bear both isolated and adjacent facets (n = 18, 4.5%). Juxtaposed facets producing a geometric or pointed morphology are present on less than a hundred examples (n = 96, Fig 6N and 6P–6S, Fig B in S1 Figs). Of complete identifiable facets (n = 1665, 99% Fig 6B, 6C and 6M–6S, Figs B and C in S1 Figs), most are convex (n = 1184, 71.1%) or flat (n = 429, 25.8), rather than concave (n = 19, 1.1%) or irregular (n = 16, 0.9%) (Table L in S1 Tables). Striations are in most cases oriented obliquely to the main axis of the facet (n = 898; 53.9%, Table L in S1 Tables) although they can also be longitudinal (n = 437, 26.2%) or perpendicular (n = 78, 4.7%). Overlapping striations include combinations of oblique and longitudinal (n = 167, 10%), oblique and perpendicular (n = 12, 0.7%), longitudinal and perpendicular (n = 3, 0.2%) and random (n = 41, 2.4%) orientations. The orientation could not be determined in only 29 cases (1.7%).

Incisions produced by scraping and scoring (Fig 6A, 6N, 6T and 6U, Fig D in S1 Figs) were identified on 111 pieces (6.4%; 2.9 kg or 7.2% by weight). Smoothed areas (Fig 6O, Fig D in S1 Figs) were detected on 71 pieces (4%; 2.2 kg or 5.5% by weight). Percussion pits (Fig 6V, Fig C in S1 Figs) were recorded on 14 (0.8%; 1.8 kg or 4.5% by weight) and are located on one end (n = 7), two ends (n = 5), or the entire surface of the piece (n = 2).

**Fig 11:**
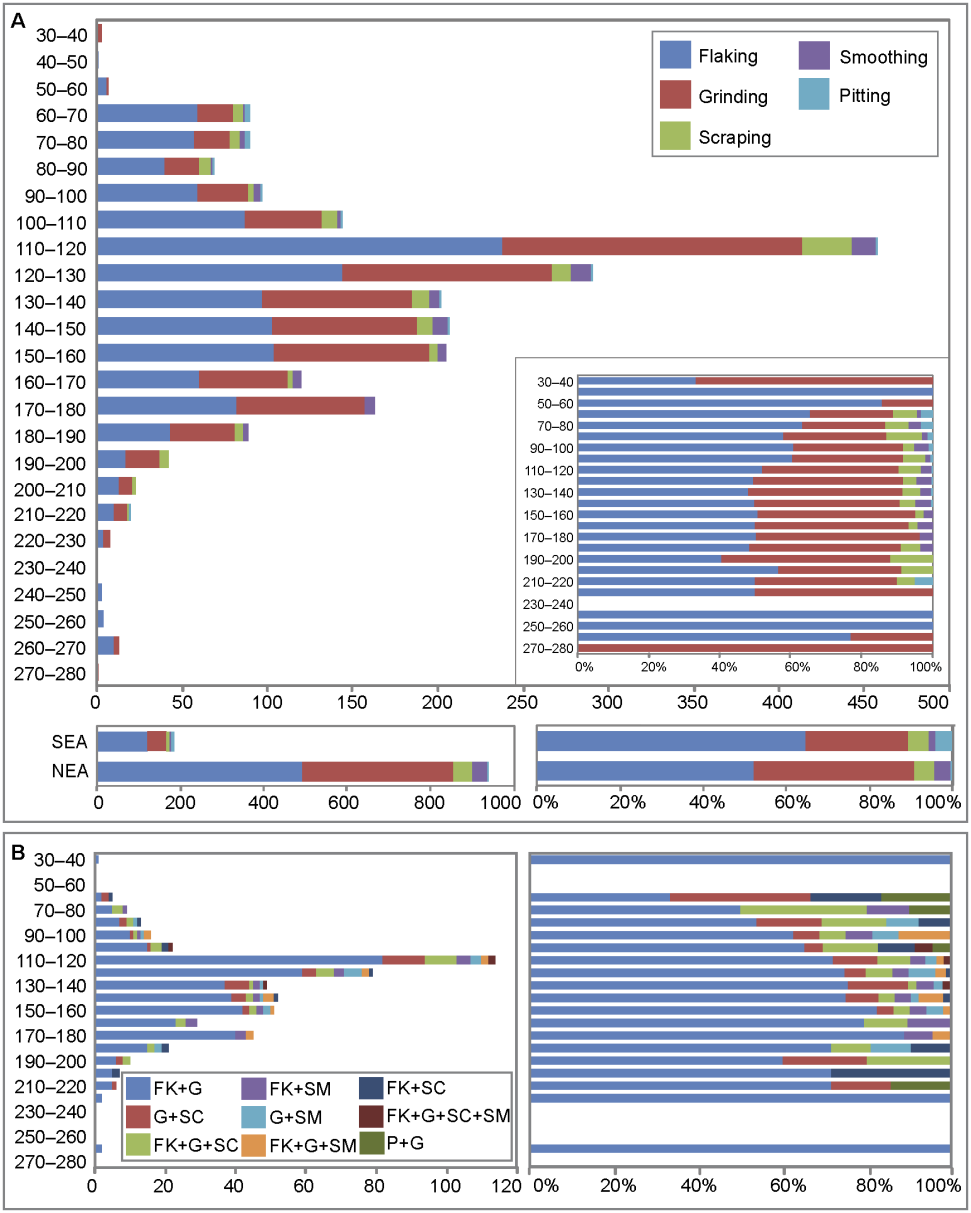
Vertical distribution of modifications identified on ochre lumps. Data is presented in number of pieces and percentages. (A) Occurrence of each modification type throughout the sequence. Separate histograms are presented for ochre pieces from the northeastern (NEA) and southeastern (SEA) accumulations. (B) Occurrence of main combinations of modifications. Ochre pieces with only one modification or combinations that appear on less than 4 pieces were excluded. FK: flaking, GR: grinding; SC: scraping; SM: smoothing; P: pitting.

Most pieces only bear one type of modification (n = 1192, 68.5%), a third associate two (n = 489, 28.2%), and only a few examples produced evidence for three (n = 51, 2.9%) or four (n = 5, 0.3%) types (Table 4, Table M in S1 Tables). Flaking is the only modification present on 756 pieces, (43.5%) grinding the only recorded on 403 pieces (23.2%), with 397 (22.8%) bearing evidence for both. Pieces with traces of scraping and grinding (n = 38, 2.2%), flaking, grinding and scraping (n = 35, 2%), or flaking associated with smoothed areas (n = 21, 1.2%) are less frequent and only a single object shows all types of identified modifications. Pieces bearing multiple modifications are found in all levels with large ochre assemblages (-60–190 cm) and combinations of different modifications remain relatively stable across the sequence (Table M in S1 Tables, Fig 11B).

**Table 4:** Combinations of modification types on single ochre pieces.

Num of pieces	SM	GR	SC	FK	P
14					
16					
403					
1					
2					
12					
4					
38					
21					
17					
35					
397					
1					
10					
1					
4					
2					
1					
756					
2					
2					

Num.: number; SM: smoothing; GR: grinding; SC: scraping; FK: flaking; P: pitting.

#### Modifications by raw materials

**Fig 12:**
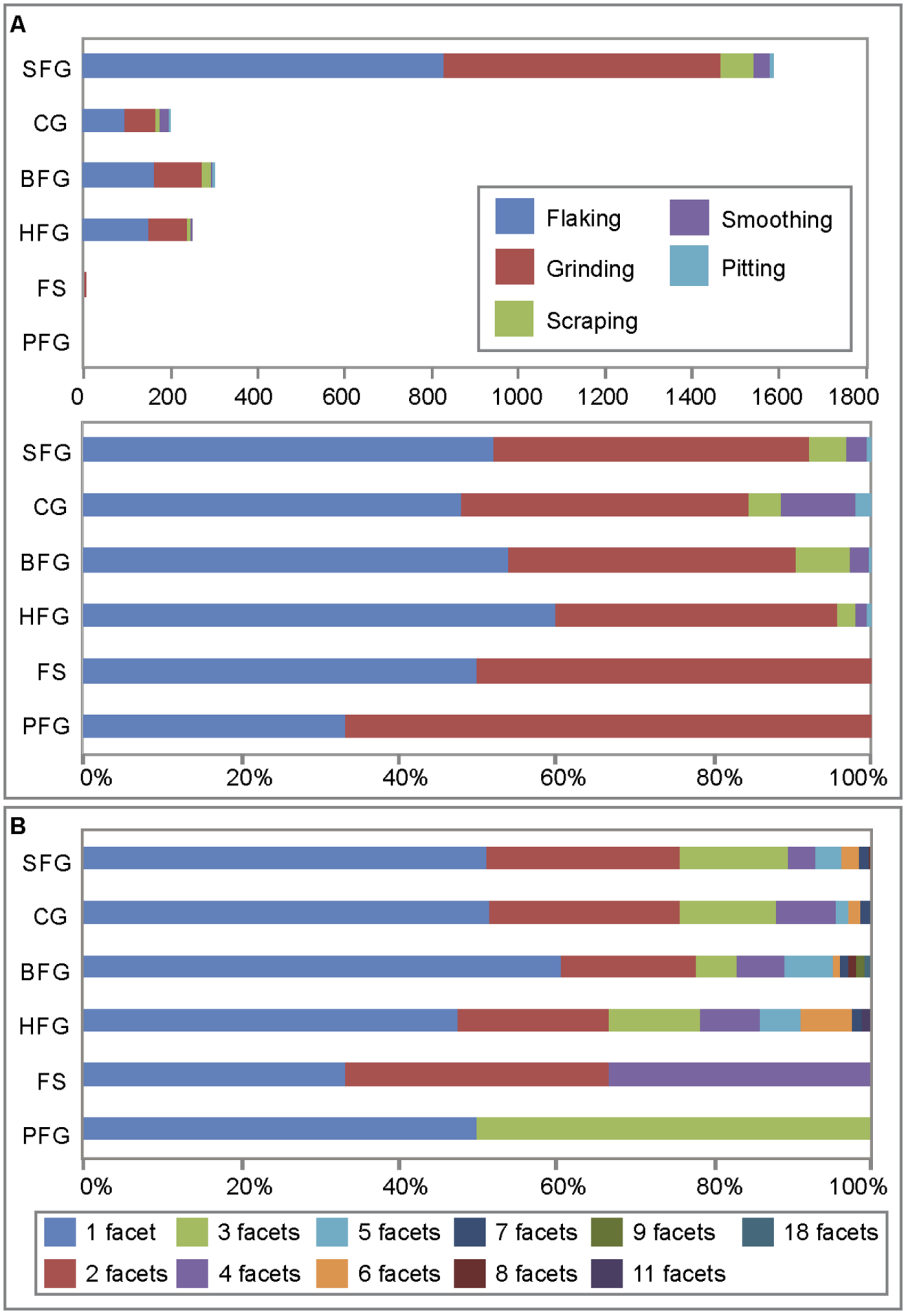
Modifications and number of facets per ochre raw material type. (A) Modifications by raw material. Data is presented in number of pieces and percentages. (B) Number of facets resulting from grinding by raw material. Data is presented in percentages.

Of the four best represented raw materials (SFG, CG, BFG, HFG), almost 60% of HFG and BFG pieces bear traces of intentional modification. This is considerably more than what is observed for SFG (46%) and CG (29%). By weight, modified ochre represent 67% and 68% of HFG and BFG respectively, 65% of SFG, and 58% of CG. The five identified types of modification (flaking, scraping, grinding, smoothing, pitting) were all observed (Fig 12, Table 3) on these four raw materials. Regardless of raw material type, half bear traces of flaking, 35–40% traces of grinding, 2.4–7% evidence of scraping. On the other hand, traces of smoothing (9.5%) and pitting (2%) are clearly more abundant with the CG. Scraping is barely represented in the HFG category, which is consistent with the hardness of this material. Single or multiple facets were observed on all types of raw materials with evidence for grinding. However, pieces with multiple facets are comparatively overrepresented in HFG, particularly in the range of 4–6 facets. More than half of pieces with facets in this raw material present more than one facet and 34% more than two, whereas facet frequencies are lower than 50% and 25%, respectively, for the other types of raw material. By weight, HFG pieces with more than two facets represent 51% of pieces with facets in this material, while this is the case in only 31%, 42% and 29% for the SFG, CG and BFG categories respectively.

All types of modification are found in similar proportions on ochre pieces of different colour. In other words, the type of techniques applied to the ochre was not dependent on their colour (Fig 7E, Table E in S1 Tables). The only possible difference is the overepresentation of pits on red and grey ochre pieces. However, this is probably linked to the fact that pitting is more common in the CG, a raw material that is often red and grey, as indicated above.

#### Changes in ochre treatment

**Fig 13:**
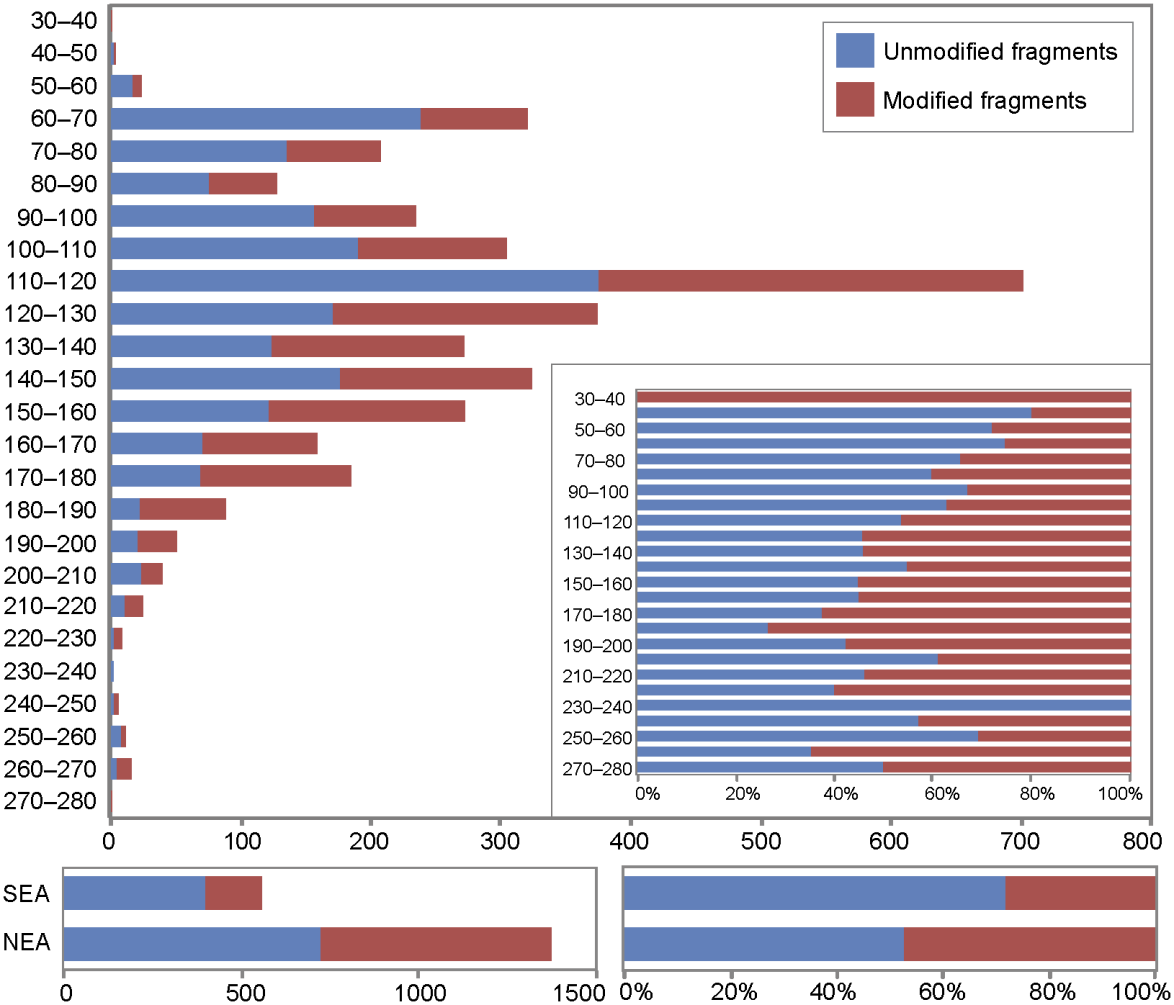
Vertical distribution of modified and unmodified ochre lumps. Data is presented in number of pieces and percentages. Separate histograms are presented for ochre pieces found in the northeastern (NEA) and southeastern (SEA) accumulations.

**Fig 14:**
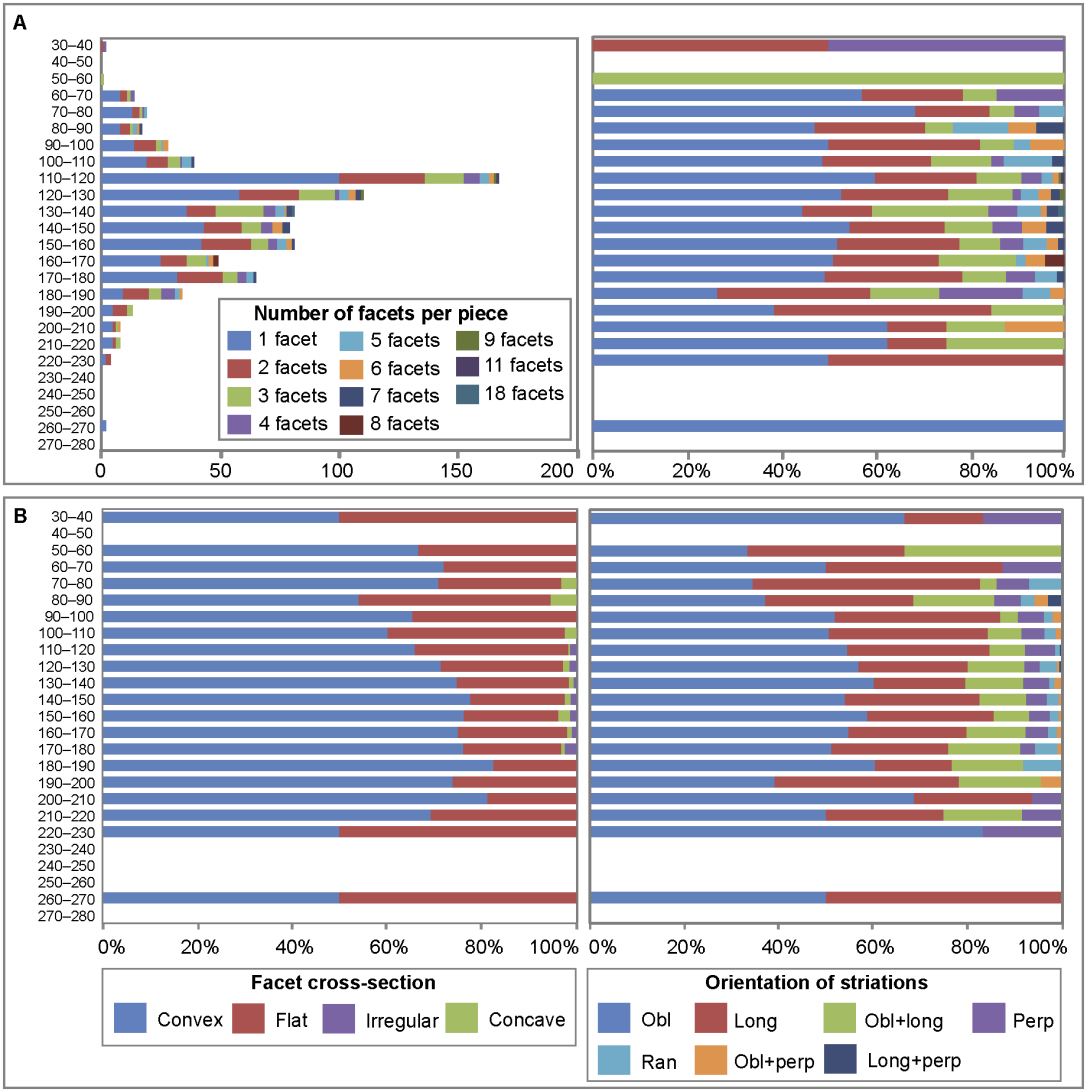
Vertical distribution of ochre lumps with facets, facet cross-sections and orientation of striations. (A) Distribution of ochre pieces with facets produced by grinding, per number of facets. Data is presented in number of pieces and in percentages. (B) Distribution of facets by cross-section type and orientation of striations with respect to the main axis of the facet. Facets with undetermined cross-sections or orientation of striations were excluded. Data is presented in percentages.

The proportion of modified ochre pieces decreases progressively from -200–210 cm to -60–70 cm spits (Fig 13, Table I in S1 Tables) when analysed by number of pieces, and from -140–150 to -40–50 when analysed by weight (Table J in S1 Tables). This trend is worth noticing, considering the large number of ochre pieces recovered in these levels. Ochre recovered from accumulations SEA and NEA reveal the same proportion of modified and unmodified ochre pieces recorded in the other squares of their respective levels. Interesting diachronic changes are also observable in the way ochre was modified (Fig 11, Table K in S1 Tables). There is a general gradual increase of flaking, scraping and pitting from the bottom to the top of the sequence. Flaking is fairly constant at about 50% between -220 and 120, and progressively increases between -120 cm to the top of the sequence (except for a slight decrease at -80–90), from around 60% to more than 80% at -50–60. Evidence for scraping progressively augments from 2.5% in the 160–170 spit to 7% in the -60–70 cm spit. Practically absent in levels between -200 cm and 110 cm, pitting accounts for 3.3% of all modifications in the -60–70 spit. In contrast, grinding becomes increasingly rare; evident on 48% of pieces in the -190–200 spit and only to 23% in the -60–70 spit. The levels in which evidence for grinding is more frequent (-90–190 cm) are also those where this type of modification is most intense, as indicated by the high number of facets per piece at these depths (Fig 14A). In levels between -160–130 cm, there is a consistent increase in the proportion of more intensively ground pieces moving up the sequence. Facet cross-sections (Fig 14B, Table L in S1 Tables) remain stable throughout the stratigraphy, with convex facets always more frequent than flat facets. Concave or irregular facets appear only between -70 and 180 cm and -110–180 cm, respectively. The orientation of striations (Fig 14B, Table L in S1 Tables) on the facets changes slightly and gradually throughout the stratigraphy, with oblique striations decreasing from 83.3% in the -220–230 spit to 33.3% in the -50–60 spit and longitudinal striations increasing from 23.1% in the -210–220 spit to 45.2% in the -70–80 spit. Facets with oblique and longitudinal or uniquely perpendicular striations remain relatively stable. Facets with random striations or oblique and perpendicular striations appear, respectively, between 70 and 190 cm and between 80 and 200 cm below datum. Ochre accumulations SEA and NEA do not differ from what was observed in the other squares of their respective levels in terms of the proportion of techniques observed (Figs 11A and 13).

#### Size and weight of ochre pieces

**Fig 15:**
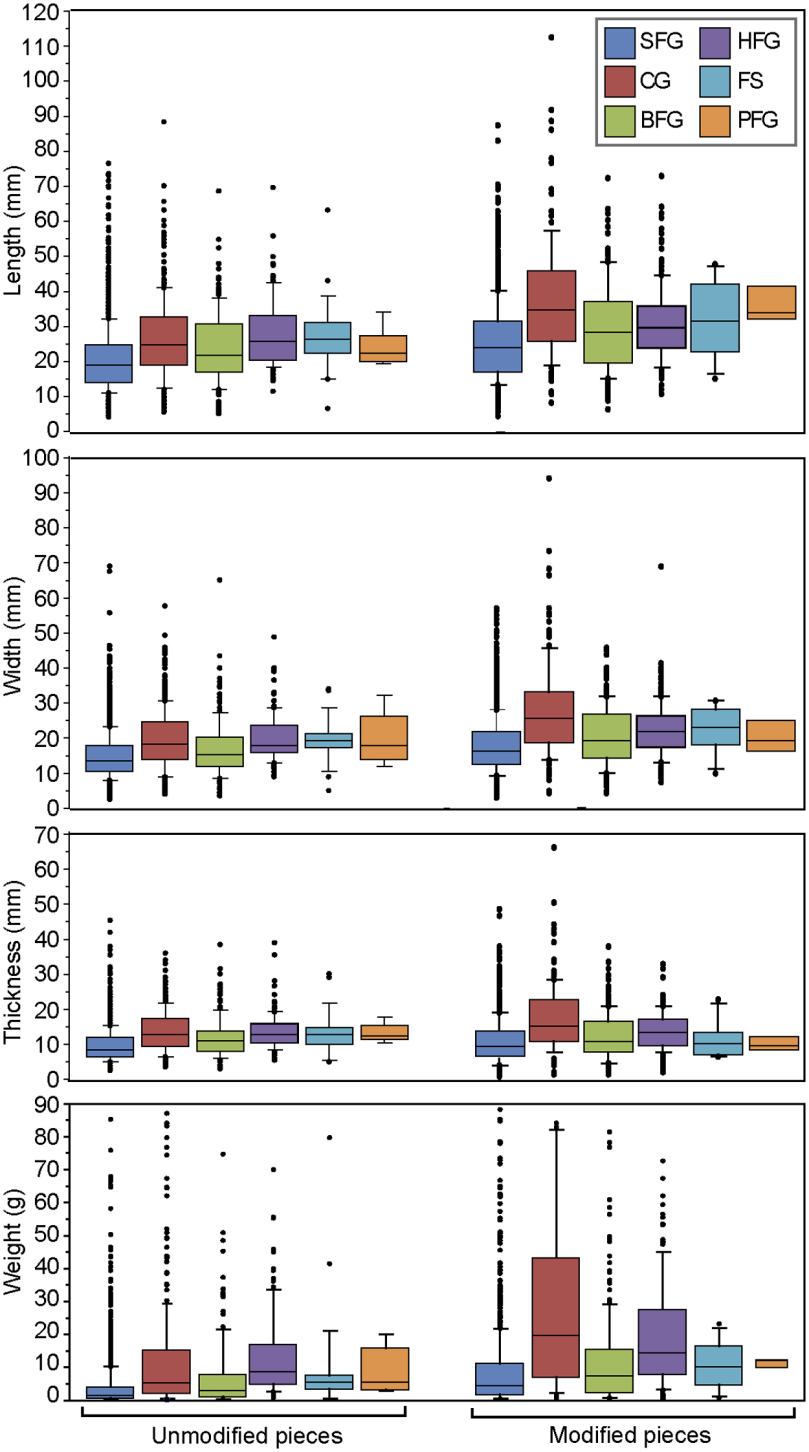
Size and weight of unmodified and modified ochre pieces by raw material type. SFG: soft fine-grained; CG: coarse-grained; BFG: banded fine-grained; HFG: hard fine-grained; FS: ferruginous sandstone; PFG: platy fine-grained.

**Fig 16:**
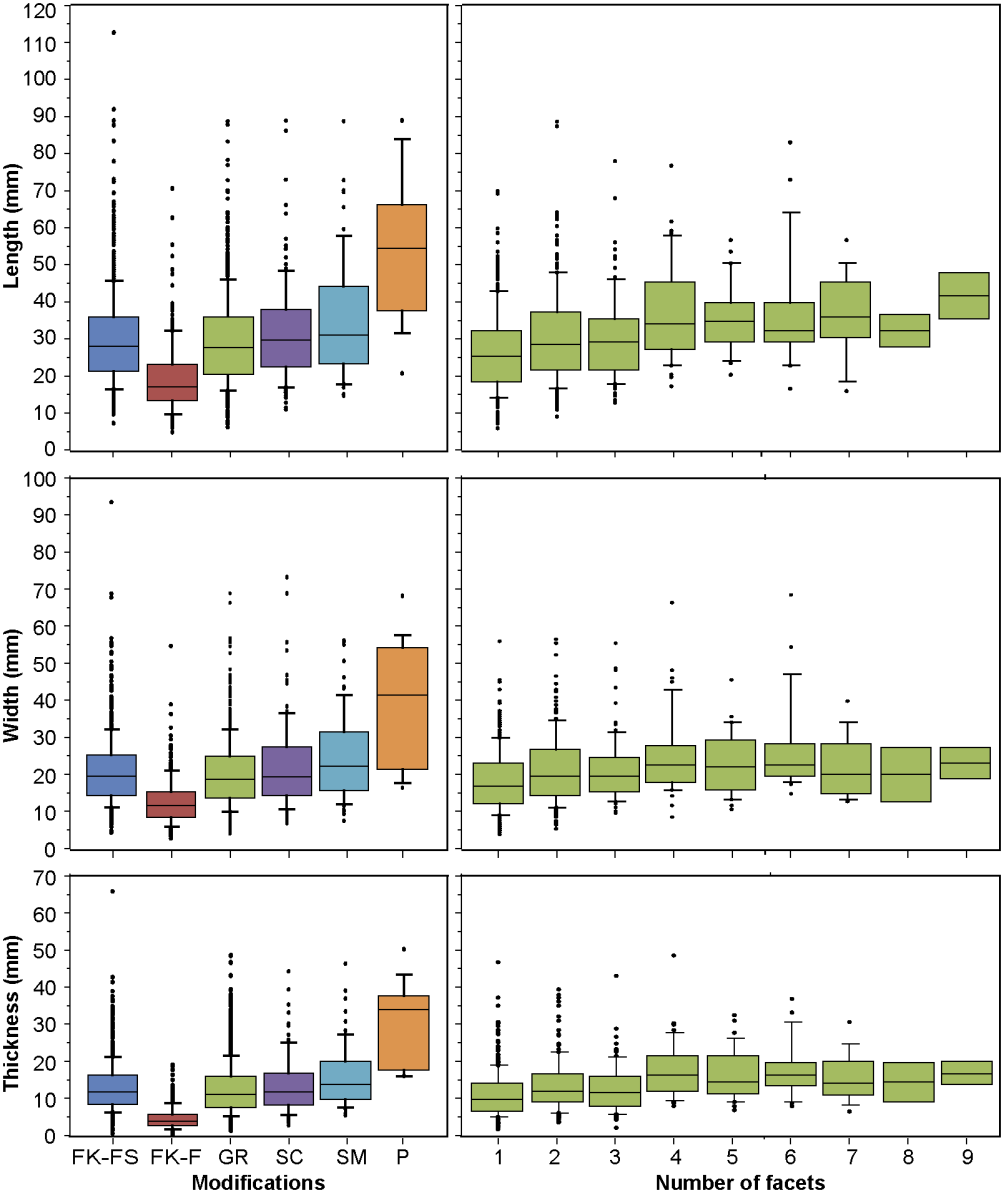
Size of modified pieces by modification type and number of facets. FK-FS: flaking, flake scars; FK-F: flaking, flakes; GR: grinding; SC: scraping; SM: smoothing; P: pitting.

Ochre pieces vary considerably in terms of size and weight (Figs 15 and 16, Tables 5 and 6). They can reach 12 cm in length (mean = 24.6 mm), 9 cm (mean = 17.2 mm) in width, 6,6 cm in thickness (mean = 10.7 mm), and 678 gr in weight (mean = 10.6 gr). No significant changes in size and weight are observed throughout the sequence (Table G in S1 Tables). This suggests that the higher number of ochre pieces in layers -180–60 cm is not due to a higher fragmentation occurring in these layers. When observing mean sizes of pieces of SFG, it appears that they are slightly smaller and lighter than what is observed with the other raw materials (Fig 15, Table 5). The opposite is seen with CG, with modified pieces of this raw material being on average three times heavier than unmodified examples. Moreover, in three categories (SFG, CG, and BFG), modified pieces are substantially larger and heavier than their unmodified counterpart. Red, dark red, orange and brown pieces tend to be lighter than grey pieces. This is consistent with the fact that pieces of SFG are generally lighter than pieces of CG (Table C in S1 Tables). No substantial differences in size and weight are recorded between pieces modified by grinding, scraping and pitting (Fig 16, Table 6). On the other hand, examples with traces of smoothing are larger and heavier than those modified with the other three techniques. Pieces bearing flake scars are similar in size to those modified by the other techniques but considerably larger and heavier than flakes (Fig 16). This implies that the size of most flakes rendered them unsuitable for grinding, scraping, pitting, and smoothing. Contrary to what one may expect, the size of the pieces does not decrease with the number of facets produced by grinding. We observe an increase in length with an increase in the number of facets, particularly in pieces with 1 to 4 facets (Fig 16). These differences are statistically significant (Table N in S1 Tables). However, pairwise comparisons are only significant when pieces with one facet are compared with pieces with 2, 4, 5 and 6 facets (Table O in S1 Tables). A slight increase in facet length on pieces with more than 3 facets is observed, and appears statistically significant (Table P in S1 Tables). Pieces with 1–3 facets show a mean facet length ranging from 19.3 to 20.6 mm, and pieces with 4–6 facets, ranging from 22.2 to 22.8 (Table 7); these two groups differ significantly from one another (Table Q in S1 Tables). All of these general trends remain stable throughout the sequence.

**Table 5:** Size and weight of modified and unmodified ochre lumps per raw material.

Size		Unmod						Mod						All pieces
		SFG	CG	BFG	HFG	FS	PFG	SFG	CG	BFG	HFG	FS	PFG	
**Length (mm)**	**Min**	1.8	3.8	2.9	9.9	4.9	18.4	5.1	8.8	6.9	11	15.8	31.8	1.8
	**Max**	78.6	91.3	70.2	71.2	64.5	33.8	87.7	112.7	72.7	73	48.4	44.1	112.7
	**Mean**	19.6	25.8	23.1	27.5	26.8	36.8	26	37.8	30	31.2	32.5	36.8	24.6
	**St dev**	10.2	12.6	11.4	10.3	11.9	6.5	11.3	17	12.8	10.5	11.8	6.5	12.2
**Width (mm)**	**Min**	0.8	2.2	1.5	7.1	3.1	10.3	3.2	4.3	4.2	7.6	9.7	15.2	0.77
	**Max**	71.0	58.9	66.8	49.5	33.6	32	56.9	93.8	45.4	68.9	30.8	26.4	93.8
	**Mean**	13.5	18.5	16.0	19	18.5	20.4	18.1	27.8	20.6	22.3	22.7	20.4	17.2
	**St dev**	7.3	9.3	9	7.4	7	5.7	8.5	13.8	8.5	7.8	7.4	5.7	9.1
**Thickness (mm)**	**Min**	0.4	1.4	0.8	3.5	3.1	8.8	1	1.6	1.7	2.4	6.7	8.1	0.4
	**Max**	45.8	36.2	38.5	39	29.8	16.8	48.5	66.1	37.9	33.2	23.4	13.2	66.1
	**Mean**	8.1	12.4	10.4	12.5	11.8	10.5	11.1	17.9	12.8	14	11.9	10.5	10.7
	**St dev**	5.1	6.7	6.3	5.6	7	2.6	6.4	9.8	6.5	5.5	5.8	2.6	6.5
**Weight (g)**	**Min**	0.1	0.1	0.1	0.8	0	2.9	0.1	0.4	0.1	0.4	0.8	8.7	0.1
	**Max**	320.4	229.9	172	192.4	79.8	19.9	27.8	678	124.3	364.5	23.3	12.6	678
	**Mean**	4.8	13	8.7	15.8	10.3	11.2	10.5	41.6	12.9	21.8	11.3	11.2	10.6
	**St dev**	13.7	21.8	19.5	25.4	17.3	2.1	21.2	77.2	17.6	31.3	7.9	2.1	25.272
**Total weight (kg)**		6.7	4.4	1.3	2.0	0.2	0.05	12.4	6.0	2.8	4	0.1	0.03	39.97
**% total weight**		16.664273	11.084026	3.356605	4.8964974	0.593395	0.114686	30.94995	15.002502	6.9954966	10.081736	0.1978734	0.084063	100
**Num of pieces**		**1404**	**342**	**155**	**124**	**23**	**5**	**1184**	**144**	**216**	**185**	**7**	**3**	**3792**

Unmod: unmodified; mod: modified; min: minimum; max: maximum; st dev: standard deviation; num: number; SFG: soft fine-grained; CG: coarse-grained; BFG: banded fine-grained; HFG: hard fine-grained; FS: ferruginous sandstone; PFG: platy fine-grained.

**Table 6:** Size and weight of ochre pieces by modification type and ochre accumulation area.

Size		All unmod	All mod	Modifications					SEA		NEA		All frag
				FK	GR	SC	SM	P	Unmod	Mod	Unmod	Mod	
**Length (mm)**	**Min**	1.8	5	5.06	6.3	11.15	14.72	20.75	6.2	10.8	1.8	5.1	1.8
	**Max**	91.2	112.7	112.7	88.8	88.84	88.84	88.84	74.8	88.8	55.9	87.7	112.7
	**Mean**	21.5	28.1	28.025	29.5	32.211	35.269	54.317	22.8	28	17.4	26.7	24.6
	**St dev**	11.1	12.5	12.564	12.3	14.408	15.78	19.753	10.5	11.5	9.6	13.2	12.2
**Width (mm)**	**Min**	0.7	3.2	3.23	4.3	7.2	8.03	16.41	4.2	6.6	0.8	3.2	0.77
	**Max**	71	93.7	93.78	68.9	73.26	56.18	68.93	47.0	56.8	45	56.9	93.8
	**Mean**	14.9	19.7	19.715	20.57	22.736	25.064	40.968	15.8	19.3	11.8	18.5	17.2
	**St dev**	8.1	9.4	9.379	9.3	12.059	11.762	16.701	7.3	8	6.8	9.3	9.1
**Thickness (mm)**	**Min**	0.4	1.04	1.04	1.7	3.03	5.75	16.42	1.8	1.9	0.4	1	0.4
	**Max**	45.8	66.09	66.09	48.4	44.22	46.62	50.48	29.6	50.5	36.2	46.6	66.1
	**Mean**	9.3	12.213	12.144	12.8	13.846	16.167	31.226	9.9	12	7.2	11.2	10.7
	**St dev**	5.8	6.942	6.774	6.9	7.891	8.455	10.764	5.5	7	4.9	7	6.5
**Weight (g)**	**Min**	0.1	0.1	0.1	0.1	0.51	0.93	9.78	0.1	0.2	0.1	0.1	0.1
	**Max**	320.4	678	678	678	402.1	279.47	364.5	90.2	279.5	84.3	277.8	678
	**Mean**	7.2	14.6	14.51	10.571	26.552	31.618	129.497	7.5	14.2	3.4	12.9	10.6
	**St dev**	17.098	31.9	32.061	25.272	61.172	51.518	110.972	12.0	30.7	7.8	25.1	25.272
**Total weight (kg)**		14.6	25.3	18.0	14.3	2.9	2.2	1.8	3.0	2.2	2.4	8.3	39.9
**% total weight**		36.5	63.2	45.1	35.8	7.3	5.5	4.5	7.5	5.5	6	20.8	100
**Num of pieces**		**2053**	**1739**	**1242**	**913**	**111**	**71**	**14**	**650**	**159**	**723**	**399**	**3792**

Unmod: unmodified; mod: modified; FK: flaking; GR: grinding; SC: scraping; SM: smoothing; P: pitting; SEA: southeastern ochre accumulation; NEA: northeastern ochre accumulation; min: minimum; max: maximum; st dev: standard deviation; num: number.

**Table 7:** Size of facets per number of facets present on the piece.

	Size (mm)	Min	Max	Mean	St dev
**Pieces with 1 facet**	**Length**	3.5	46.7	19.3	8.9
	**Width**	0.9	37.4	11.1	6.6
**Pieces with 2 facets**	**Length**	3.7	77.9	20.6	11.2
	**Width**	0.9	39.1	10.7	7.0
**Pieces with 3 facets**	**Length**	2.1	47.8	19.4	8.9
	**Width**	1.0	36.9	9.8	6.4
**Pieces with 4 facets**	**Length**	4.8	97.3	22.7	12.7
	**Width**	1.5	35.3	11.2	6.7
**Pieces with 5 facets**	**Length**	5.0	52.0	22.2	9.6
	**Width**	1.4	29.9	10.4	5.8
**Pieces with 6 facets**	**Length**	7.0	77.9	22.8	11.5
	**Width**	0.8	46.0	11.9	7.7
**Pieces with 7 facets**	**Length**	3.0	44.1	21.7	11.1
	**Width**	1.3	28.3	9.6	6.1
**Pieces with 8 facets**	**Length**	4.6	30.3	14.5	8.2
	**Width**	2.5	18.9	6.8	3.8
**Pieces with 9 facets**	**Length**	3.2	42.9	20.0	12.3
	**Width**	1.2	17.7	9.6	5.5
**Piece with 11 facets**	**Length**	14.4	26.7	21.3	4.2
	**Width**	2.9	16.4	10.0	4.5
**Piece with 18 facets**	**Length**	5.3	27.5	14.4	5.7
	**Width**	4.2	20.7	8.6	4.3
**All facets**	**Length**	2.1	97.9	20.4	10.3
	**Width**	0.8	46.0	10.6	6.6

Min: minimum; max: maximum; st dev: standard deviation.

### Surface texture analysis

**Fig 17:**
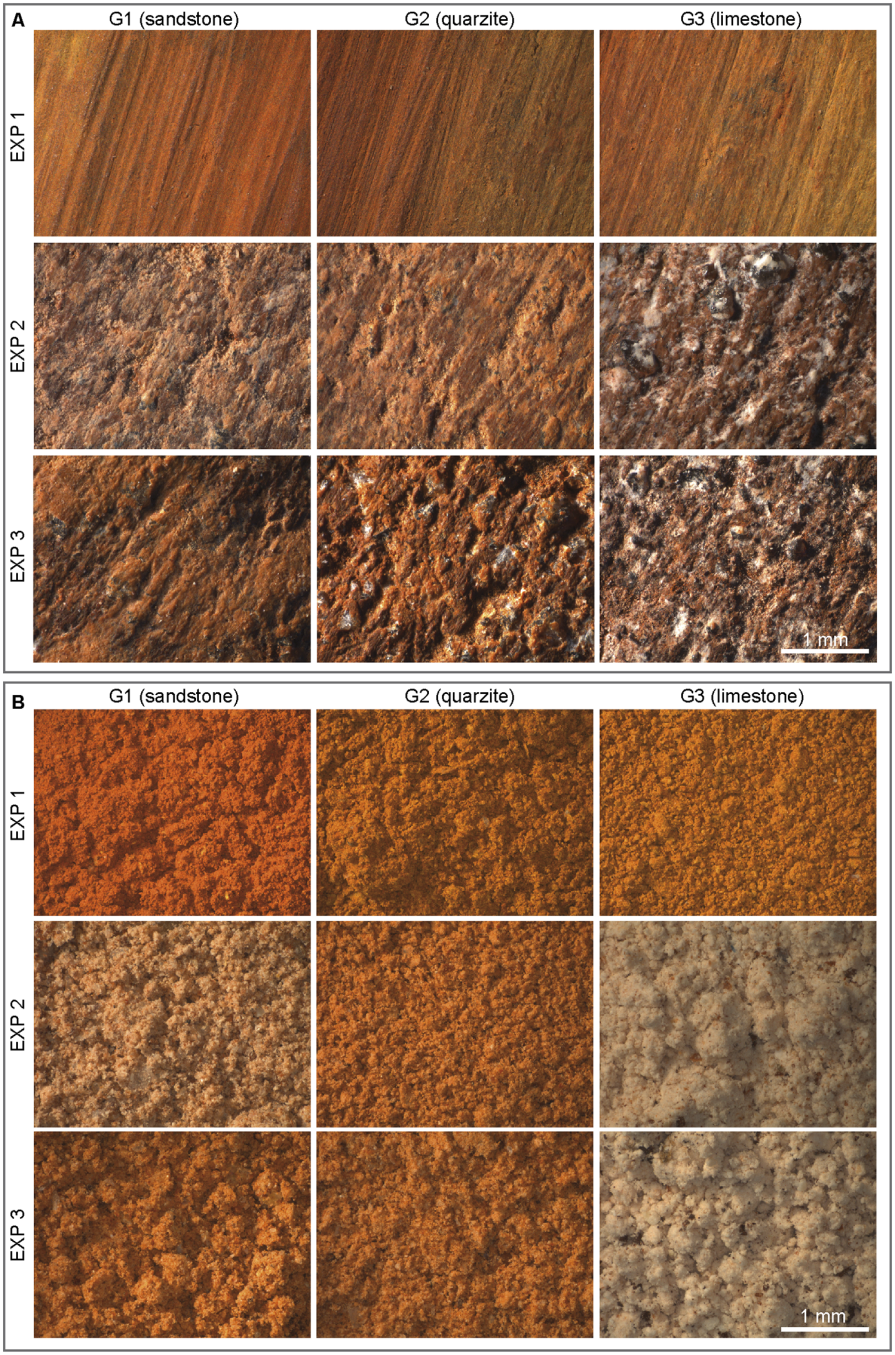
Facets and powder produced experimentally. Photos of facets (A) and experimental powder (B) produced from EXP1, EXP2 and EXP3 with sandstone, quartzite and limestone grindstones.

**Fig 18:**
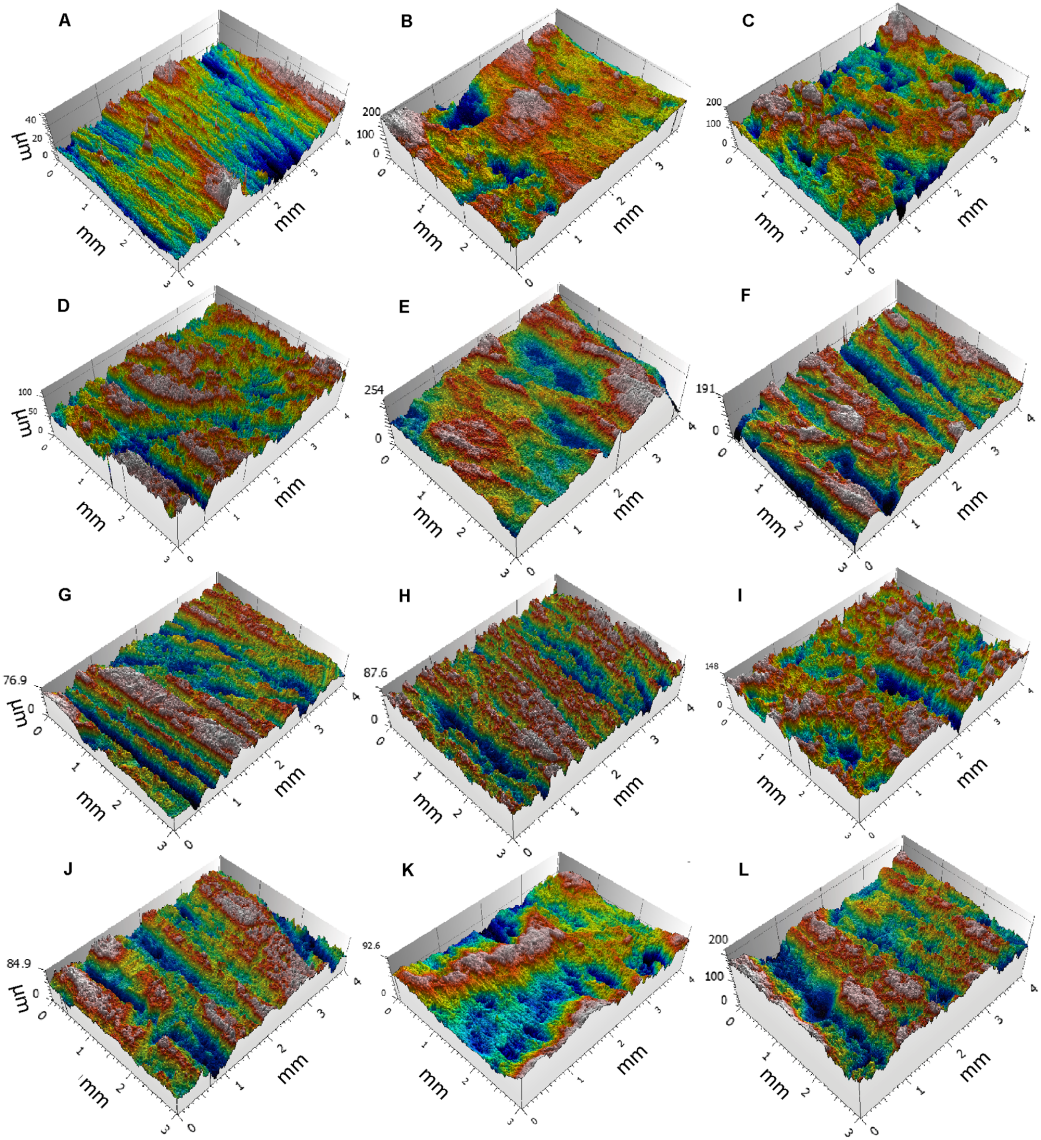
3D images produced by confocal microscopy of experimental and archaeological facets. (A) Facet on EXP1 produced with a limestone grindstone. (B) Facet on EXP2 produced with a quartzite grindstone. (C) Facet on EXP3 produced with a quartzite grindstone. (D, E) Facets on ochre piece PE102. (F) Facet on ochre piece PE987. (G) Facet on ochre piece 1491. (H) Facet on ochre piece 1493. (I) Facet on ochre piece 1499. (J) Facet on ochre piece 1677. (K) Facet on ochre piece 1700. (L) Facet on ochre piece 1806.

**Fig 19:**
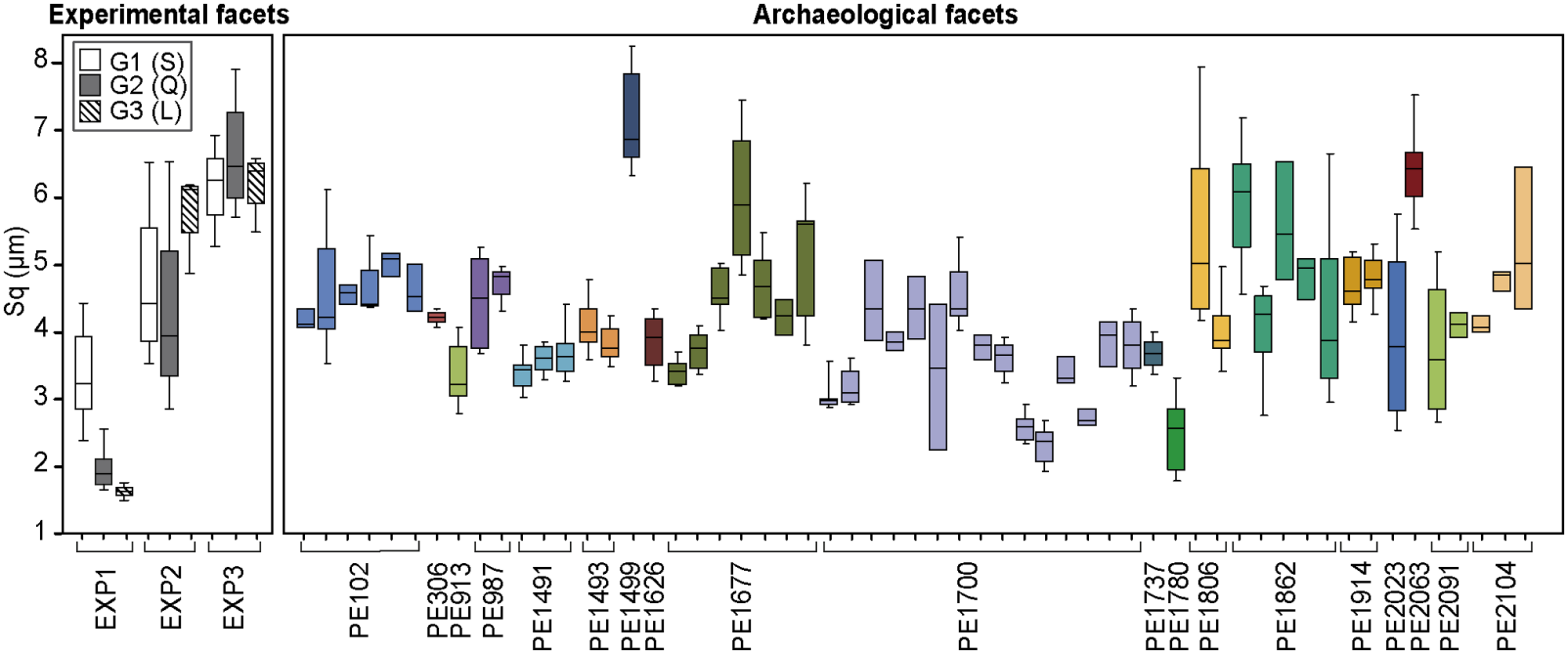
Sq values for experimental and archaeological facets. Sq values of experimental facets are presented by ochre pieces (EXP1, EXP2 and EXP3) and grindstone on which they were processed (S: sandstone; Q: quartzite; L: limestone). Colours of boxplots representing Sq values of archaeological facets represent facets from single ochre pieces.

**Fig 20:**
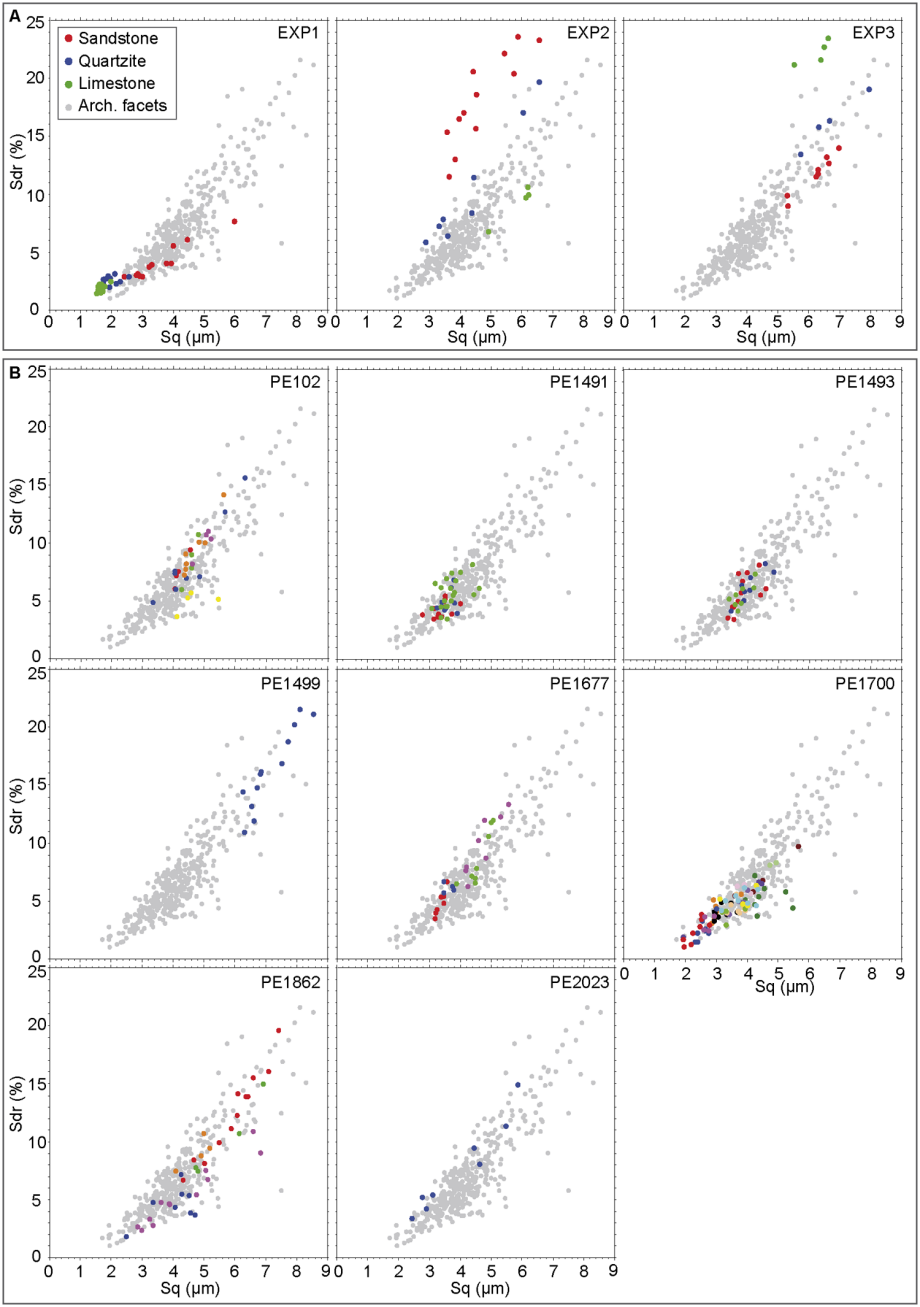
Sq and Sdr values in experimental and archaeological facets. (A) Results of rugosimetric analysis on experimental facets from EXP1, EXP2 and EXP3. (B) Results of rugosimetric analysis on archaeological pieces. Dots of the same colour identify measurements taken on the same facets. Grey dots identify the overall variability of the archaeological sample.

Grinding three ochre pieces of different textures on sandstone, quartzite and limestone grindstones (Figs 17 and 18) produces facets characterised by clearly different roughness values (Figs 19 and 20). The lowest values (Fig 19), reflecting a smoother, less complex surface texture, were obtained with fine-grained ochre (EXP1) irrespective of the grindstone used. Medium and very high values are associated, respectively, with coarse (EXP2) and very coarse (EXP3) ochre pieces. In addition, EXP1 features considerable differences in roughness according to the grindstone used, with low, medium and high values associated with limestone, quartzite and sandstone, respectively (Fig 19). Such a clear trend is not observed with EXP2 and EXP3. In EXP2, the highest Sq values (Fig 19) are observed on the facet produced on limestone with lower values for quartzite and sandstone. The highest values from EXP3 were obtained on quartzite with comparable lower values obtained for sandstone and limestone (Fig 19). The above pattern can be explained by the properties of the grindstones and the ochre pieces used. Roughness on fine-grained ochre (EXP1) is primarily determined by the texture and hardness of the grindstone. On harder and coarser ochre (EXP2), only the hardest grindstone is able to flatten the ochre surface, resulting in lower roughness values than those obtained with softer grindstones such as limestone. The roughness produced by limestone grindstones mostly depends on the ochre’s natural texture and grain hardness, rather than the properties of the grindstone itself. With the coarsest ochre (EXP3), none of the grindstones are able to flatten the natural internal texture of the material, producing high roughness values. This implies that roughness measurements can identify the type of grindstone used only when homogeneous and very fine-grained ochre is ground.

Sq values recorded on archaeological facets roughly overlap with those produced experimentally, with most ranges being comparable to those of EXP1 on sandstone and EXP2 on quartzite and sandstone (Fig 19). Extremely low values produced by grinding EXP1 on limestone were not observed on archaeological facets, suggesting that this ochre type was not or only rarely ground on limestone. Alternatively, taphonomic processes may also be responsible for texture modification, leading to increased Sq values for some archaeological specimens. This hypothesis needs to be tested experimentally in the future. As most of the analysed archaeological ochre pieces are composed of raw materials with textures between EXP1 and EXP2, their roughness values likely reflect both the hardness of the grindstone used and the texture/hardness of the pieces themselves.

Sdr and Sq values show that EXP2 processed on sandstone and EXP3 processed on limestone have facets that are rougher than the archaeological facets (Fig 20). Roughness values recorded on facets belonging to the same ochre piece (Figs 19 and 20) identify cases in which facets show similar values (PE102, PE 987, PE1491, PE1493, PE1914) and others in which some facets show clearly different values (PE1677, PE1700, PE1806, PE1862, PE2104). Our experimental results suggest the first pattern likely reflects cases in which several facets were ground on the same type of grindstone, with the second resulting from facets ground on different grindstones, possibly during different grinding sessions.

### Particle size analysis

**Fig 21:**
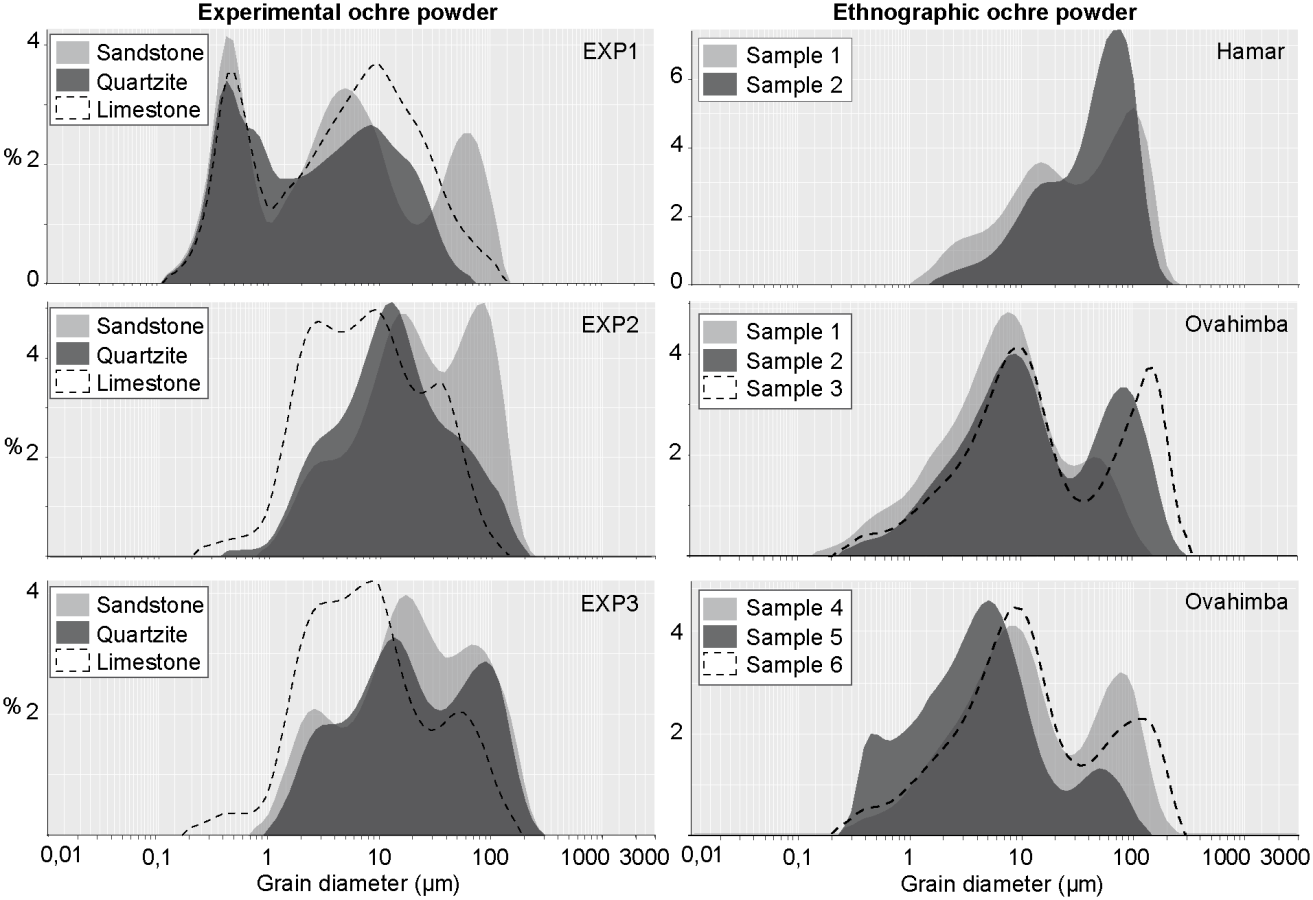
Results of granulometric analysis on experimental and ethnographic ochre powder. Grain size distribution of nine experimental ochre powder samples produced with EXP1, EXP2 and EXP3, using sandstone, quartzite and limestone grindstones, two samples produced by Hamar women, and six samples produced by Ovahimba women.

Between 40 and 390 mg of ochre powder per facet were produced during grinding experiments. Granulometric analysis shows that regardless the grindstone used, powders produced with EXP1 are finer than those produced by EXP2, which is finer than EXP3 (Fig 21). The particle size distributions are very similar in the case of EXP2 and EXP3, while EXP1 is characterised by a very fine mode indicating a clayish composition (Fig 21). Powders produced by grinding EXP2 and EXP3 on quartzite and sandstone are respectively composed of two and three main modes comparable to those observed in powders produced by Ovahimba women. Hamar powder is instead mainly composed of fine sand and small amounts of silt and clay (Fig 21). In addition, it is observed that ochre powder produced with sandstone is coarser than that produced with quartzite.

## Discussion and conclusion

Archaeologists working on the Middle Stone Age have primarily relied on lithic technology to identify evolutionary trends in regional cultural trajectories. Data accumulated over the last two decades have broadened our understanding of the Middle Stone Age by providing insights into a variety of cultural adaptations beyond those related to lithic technology and typology. Newly acquired data on, for example, bone tool and mastic production, personal ornamentation or graphic expressions demonstrate the complexity of Middle Stone Age cultures. However, the majority of information concerning these cultural innovations remains sporadic in nature, suggesting that while new cultural traits likely emerged at some sites during different periods they are insufficient to reliably and accurately track diachronic changes. This shortcoming is mainly due to the sparsity of sites at which these innovations have been documented, as well as the small sample sizes and relatively limited occurrences of pertinent artefacts. Documenting the pace and nature of such changes is however key to producing a comprehensive view of individual MSA cultural trajectories, reconstructing the rate of cultural change, and inferring mechanisms of cultural transmission specific to these societies. Although almost ubiquitous at cave or rockshelter MSA sites (particularly in Southern African sites after ca. 170 ka [4,16]), only few sites with long stratigraphic sequences that have yielded a continuous record of ochre use have been studied in detail [4,35,40,56,58]. Porc-Epic is the only one at which, with the exception of the lower and upper-most layers, behavioural trends are identified based on a very high density of modified pieces per 10 cm spit.

Although uncertainties still surround the exact chronology of the Porc-Epic sequence, radiocarbon ages suggest it covers at least 4,500 years, and indicate that ochre use probably begun around or before 45 cal kyr BP, and was intense at ca. 40 cal kyr BP [41].

In this respect, it is noteworthy that our results identify continuity or gradual changes rather than any abrupt shifts in the way ochre was acquired and treated during the accumulation of the site’s late MSA layers. The robustness of these trends is supported by the parallel analysis of the two areas (SEA and NEA) with the highest ochre concentrations. In all recorded variables, these two areas systematically show results comparable to those obtained for the overall ochre assemblage.

With the exception of an increase of the HFG type approximately from 2% to 20% at -80–150, the proportions of the different raw material types does not change substantially throughout the sequence. This probably reflects the persistent function of different types of ochre through time. Even though proportions of raw material are relatively stable, concomitant gradual changes in the morphology of ochre pieces were identified. The proportion of slabs increases with time, that of irregular pieces decreases, and that of nodules and pebbles does not considerably change throughout the stratigraphy. Slabs and irregular pieces include pieces with fresh edges that show little or no signs of water transport. This suggests that Porc-Epic MSA inhabitants searched in and around the wadi for the same raw materials, regardless of their morphology. In this respect, it is noteworthy that each raw material category used at Porc-Epic is characterised by a range of colours (Table D in S1 Tables). Continuity in raw material choice through time supports the hypothesis that Porc-Epic late MSA inhabitants provisioned themselves over a considerable duration with ochre types featuring different textures, hardness, density, colouring power, and shades. Although most of the colour types fall within the broad category of “red”, a small proportion of pieces show colour features that appear to fall outside this category. This raises the question of whether different shades of “red” and other colours were perceived as distinct categories by Porc-Epic inhabitants.

Continuity in raw material use equally tracks gradual changes in the way ochre pieces were processed. Porc-Epic ochre record features an exceptionally high proportion of modified pieces, with flaking and grinding representing the most common techniques applied. Modified ochre pieces progressively decrease from the bottom to the top of the sequence with flaking, scraping and pitting becoming increasingly frequent, smoothing remaining stable, and grinding progressively dropping. This trend indicates that grinding ochre pieces against lower grindstones gradually becomes less prevalent and is partially replaced by flaking. It has been shown at a number of sites (for example Blombos, see [35]) that ochre was flaked and subsequently crushed to produce ochre powder. Since ochre pieces showing evidence for flaking often bear other types of modifications at Porc-Epic, this interpretation seems likely. In very few cases (17 ochre bladelets and 2 transverse scrapers), iron-rich rocks may not have been part of a reduction sequence leading to the production of ochre powder, but this appears as anecdotal. Noteworthy gradual changes through time are observed in the way the ground facets were produced. While the proportion of convex and flat facets does not change meaningfully through time, the orientation of striations progressively evolve from oblique to longitudinal with respect to the main axis of the facet. The fact that concave and irregular facets, and facets with random or overlapping oblique and perpendicular striations only appear in levels with the highest occurrences of ochre may indicate the work of apprentices. However, pieces with facets showing these characteristics are not significantly different from the others in terms of raw material and size. If the less precise motions that produced the irregular facets present in these layers results from training, the trainees were given the same type of ochre routinely used at the site by “expert” ochre producers.

The gradual shift through time between the two most frequently used techniques at the site, flaking and grinding, does not correspond to a shift in the weight of ochre processed with these techniques nor in the type and amount of raw materials processed. Contrary to expectation that flaking would have been more frequently applied to the coarse ochre type CG to produce coarse powder, and grinding or scraping to the SFG, BFG or HFG types to produce fine powder, the three techniques were applied in a comparable way to all types of raw material. The fact that HFG is comparatively more represented among the intensively utilised pieces is probably meaningful (see below), as it may be linked to the fact that this raw material is the most likely to produce a fine-grained and bright red powder. The higher frequency of smoothing and pitting on the CG type may be due to the fact that grinding does not always produce diagnostic striations on coarse-grained raw materials and is therefore more difficult to identify, and that pitting on these heavy pieces results from attempts of breaking them or use for crushing more friable ochre.

Results of the rugosimetric analysis of the ground facets and the size of ground pieces with more than one facet suggests curation of ground objects. The former shows that some pieces bear facets produced with different grindstones, arguably at different times. The latter indicates that pieces with just one facet are significantly smaller than those with more facets and that a trend towards bigger pieces is observed with the increase in the number of facets. This finding is consistent with the idea that large pieces were ground a number of times to produce small quantities of ochre powder. Alternatively, it may be argued that smaller pieces only bear one facet because they are more difficult to manipulate. The latter is contradicted by differences in the number of facets on pieces of different raw materials. Pieces of the fine-grained HFG type display, comparatively, a higher number of facets than ochre pieces made of the other raw materials. This implies that Porc-Epic inhabitants more intensively ground ochre pieces when they were made of the best available raw material. The small size of some of the HFG ochre pieces did not prevent their intense modification.

It is likely that crushing ochre was also not applied to produce substantial amounts of ochre powder. Evaluating the amount of ochre powder produced with this technique is of course difficult, as it leaves behind little tangible evidence, i.e. small fragments produced by crushing and pits on the grindstones. However, the application of these techniques by Hamar [96] or Ovahimba women [22] shows that in order to produce large quantities of powder with this technique, ochre is crushed on large lower grindstones, and then ground with upper grindstones. Most lower grindstones at Porc-Epic Cave are relatively small in size, only few show traces of pitting [38], and no upper grindstones show facets or striations referable to grinding ochre against a lower grindstone. It therefore appears that unless this action was carried out on grinding tools left outside the excavated area, which is contradicted by the spatial co-occurrence of processing tools and ochre concentrations, this technique was also employed to produce small quantities of powder.

The production of small amounts of ochre powder is usually considered more consistent with symbolic activities, such as body painting, the production of patterns on different media, or for signalling [4,5,21]. However, small quantities of ochre powder can also be used for medicinal purposes [24] or hafting. It has been shown that only 5 g of ochre powder are required to produce an adhesive compound for hafting a tool [25]. Some functional activities involve larger quantities of ochre powder. Tanning hides, for instance, require more than 2 kg of ochre powder for a medium-sized antelope [21]. Use of ochre as a sun-block requires 60 g of red ochre powder every 2 or 3 days [30], and use as an insect repellent [105] would presumably require a similar quantity. Our experimental grinding of ochre suggests, however, that many facets present on the Porc-Epic pieces result from grinding episodes that produced less than 0.4 g of powder and that, as indicated by rugosimetry, pieces with multiple facets were in some cases curated and ground at different times to produce small amounts of powder. Unless powder obtained from grinding different pieces and perhaps powder obtained by crushing pieces was mixed together, some grinding episodes recorded at Porc-Epic better fit the hypothesis that ochre powder was used for symbolic rather than for functional purposes. This observation is valid for the entire Porc-Epic sequence considering that the number of facets shows no meaningful changes throughout the stratigraphy. The discovery of an ochre-stained pebble half covered with ochre residues as if dipped in an ochre-rich liquid medium to paint the object or to use it as a stamp to apply pigment to soft materials further supports the symbolic hypothesis [38]. Of course this conclusion does not imply that all ochre powder produced at the site was used for symbolic activities. Particle size analysis of experimentally ground ochre demonstrates that the use of different grindstones and ochre types produce ochre powders of different coarseness. This implies that ochre pieces presenting facets with clearly different roughness values produced ochre powder of different granulometry. The use of powder of different coarseness for distinct purposes is supported by particle size analysis of ethnographic samples. Powder produced by Ovahimba women for body painting is finer than that produced by Hamar women for their hairdress.

The above trends have clear implications for the interpretation of changes in the amount of ochre processed at the site. Considerable inter-layer variation in ochre quantities, which peaks in the spits between 100 and 130 cm below datum, does not correspond to any marked change in raw material, size and weight of the ochre pieces, or processing technique. Continuity in weight throughout the stratigraphy contradicts the hypothesis according to which the increase in ochre pieces in layers -180–60 would be result of a higher fragmentation occurring in those layers. The observed trend supports the interpretation that the larger amount of ochre found in those layers results from an increased need for ochre powder in order to fulfil the same types of functions. This observation, and the fact that there are no major changes in lithic technology throughout the sequence [41,73,76], supports the hypothesis that the site was either more frequently visited during the accumulation of the richer layers or visited by a larger group. Alternative interpretations seem less likely. Increase of ochre pieces in some archaeological layers of MSA sites has been attributed to better availability of local ochre sources during a relatively short lapse of time that would have motivated people to be less discriminating in the choice of the raw material brought to the site [5]. This hypothesis seems contradicted in our case by continuity in the proportion of the different raw materials brought to the site. Increase in some activities, for example body painting, would have likely resulted in the prominence of one type of raw material over the others, which is not the case.

Refining the dating of the sequence and the acquisition of more precise environmental data may allow us to establish whether an increase in occupation intensity coincided with, and was perhaps in some way triggered by environmental changes that created favourable conditions for demographic expansion.

Patterns of continuity observed at Porc-Epic in ochre acquisition, processing and use reflect persistence through time in the exploitation of available geological resources, and the functions in which iron-rich rocks were used by late MSA groups of the Horn of Africa. A gradual shift was, however, documented in the preferred processing techniques and motions. Understanding the mechanisms behind the transmission of cultural practices related to ochre use is only at its beginning, and a solid record, such as that from Porc-Epic Cave, is needed to draw informed, testable hypotheses. Considering the large amount of ochre used at the site, patterns of continuity and change likely reflect a cohesive behavioural system shared by all community members and consistently transmitted through time.

## Supporting information

S1 Figs Ochre pieces from Porc-Epic Cave. Photos of the pieces and modifications.
(PDF)

S1 Tables Detailed results of the technological analysis of ochre pieces. Colour, raw material and modifications of ochre pieces.
(PDF)
