# Supplementary material for: Patterns of change and continuity in ochre use during the late Middle Stone Age of the Horn of Africa: The Porc-Epic Cave record
Source: PLoS One. 2017 May 24;12(5):e0177298. doi: 10.1371/journal.pone.0177298 (PMC5443497; doi:10.1371/journal.pone.0177298)
Supplement: S1 Figs — Photos of the pieces and modifications. (PDF) [file pone.0177298.s001.pdf]

Patterns of change and continuity in ochre use during the late Middle Stone Age of the Horn of Africa: the Porc-Epic Cave record

Daniela Eugenia Rosso*, Francesco d'Errico, Alain Queffelec

* Corresponding author
E-mail: d.rosso@pacea.u-bordeaux1.fr (DR)

S1 Figures. Ochre pieces from Porc-Epic Cave.

Photos of the pieces and modifications.

Figure A. Flaked ochre pieces. 2
Figure B. Ochre pieces with traces produced by grinding 3
Figure C. Ochre pieces with traces produced by grinding and pitting 4
Figure D. Ochre pieces with traces produced by scraping and smoothed areas 5
Figure E. Unmodified ochre pieces 6

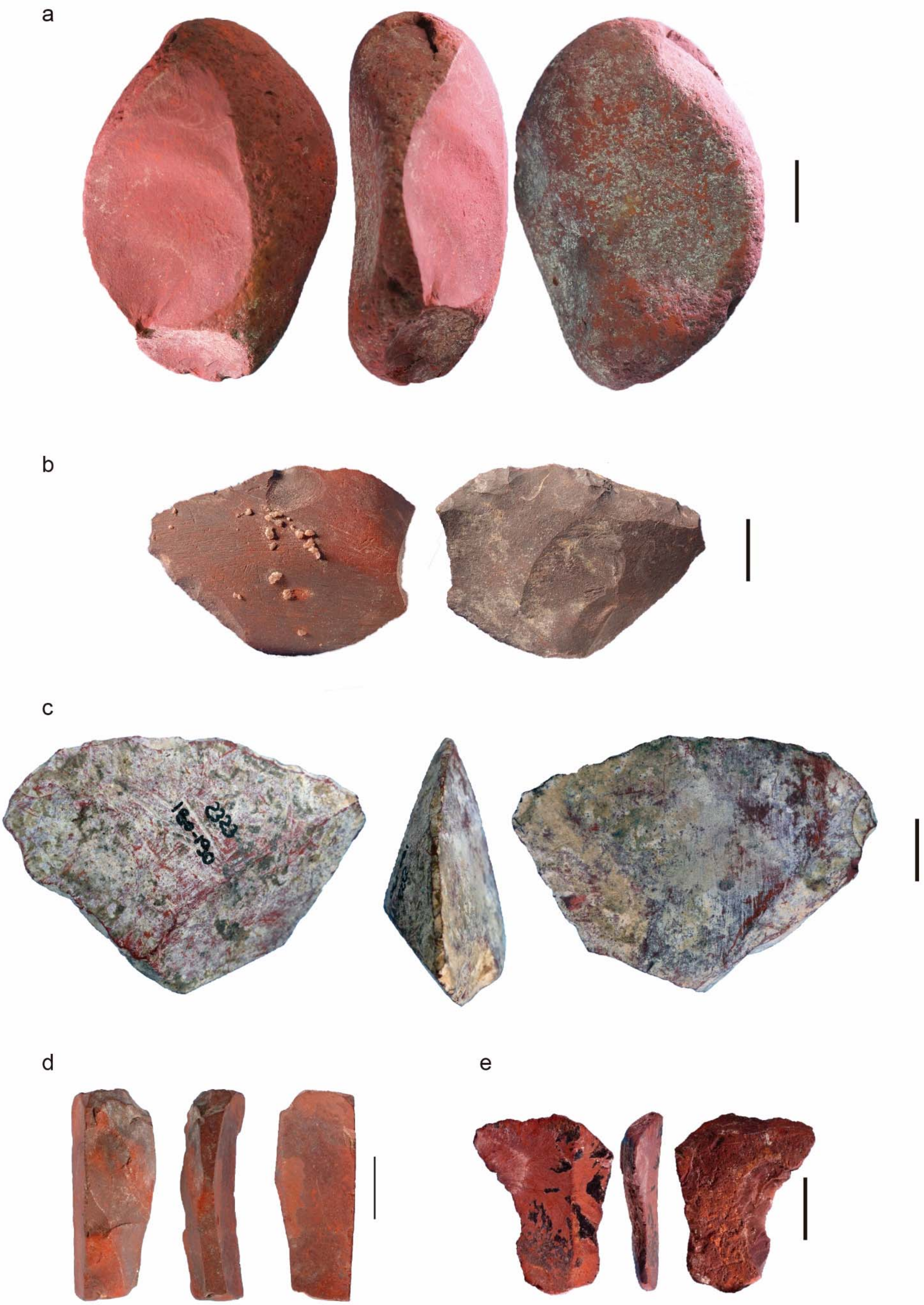

Fig A. Flaked ochre pieces. (a) Flake scar, ochre piece PE2563, SFG. (b) Scraper, ochre piece PE312, SFG. (c) Scraper, ochre piece PE3358, SFG. (d) Bladelet, ochre piece PE420, SFG. (e) Flake, ochre piece PE2063, SFG.

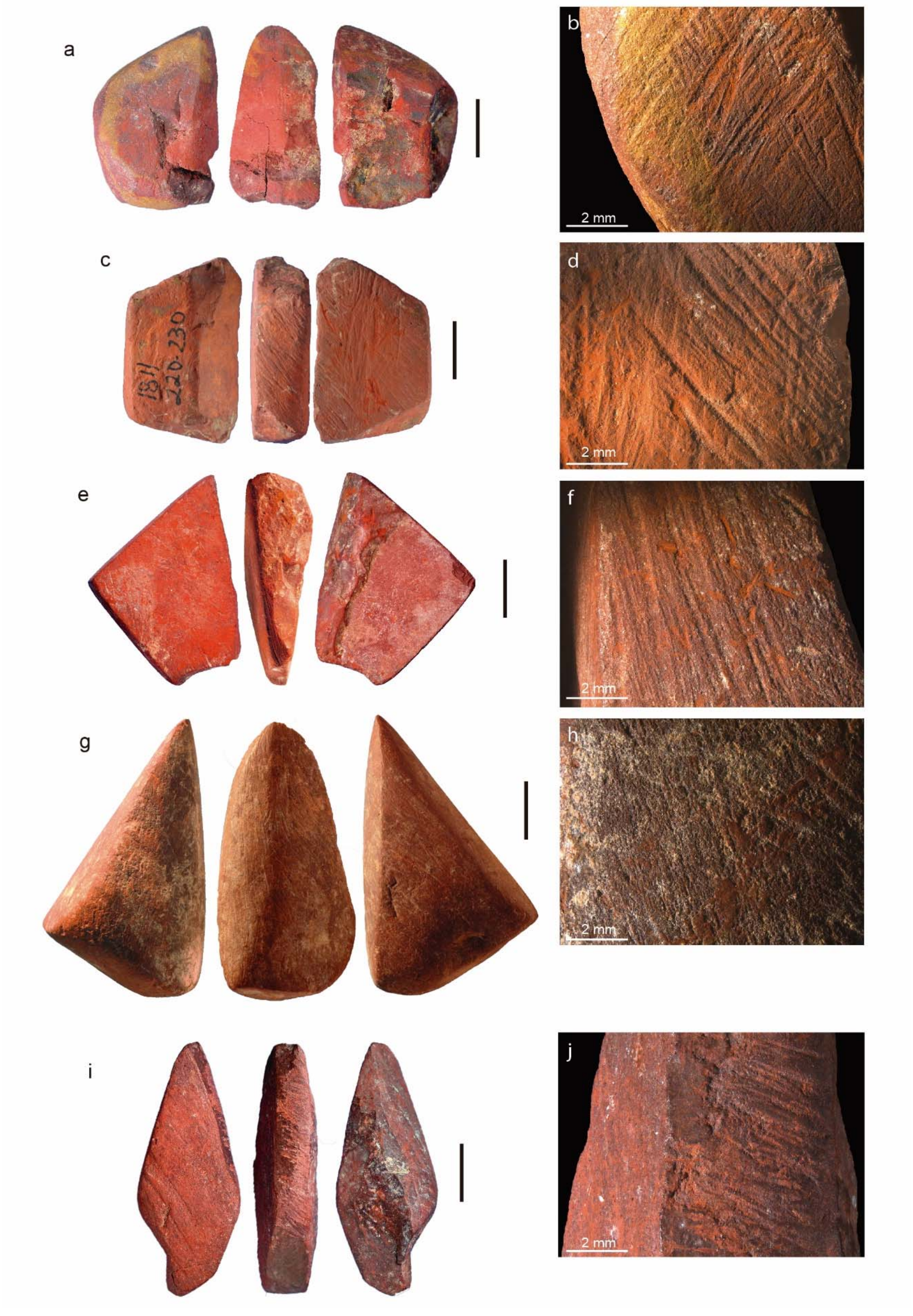

Fig B. Ochre pieces with traces produced by grinding. (a, b) Ochre piece PE102 and photo of striations produced by grinding on the same piece, BFG. (c, d) Ochre piece PE987 and photo of striations produced by grinding on the same piece, SFG. (e, f) Ochre piece PE1493 and photo of striations produced by grinding on the same piece, SFG. (g, h) Ochre piece PE1677 and photo of striations produced by grinding on the same piece, SFG. (i, j) Ochre piece PE1862 and photo of striations produced by grinding on the same piece, SFG.

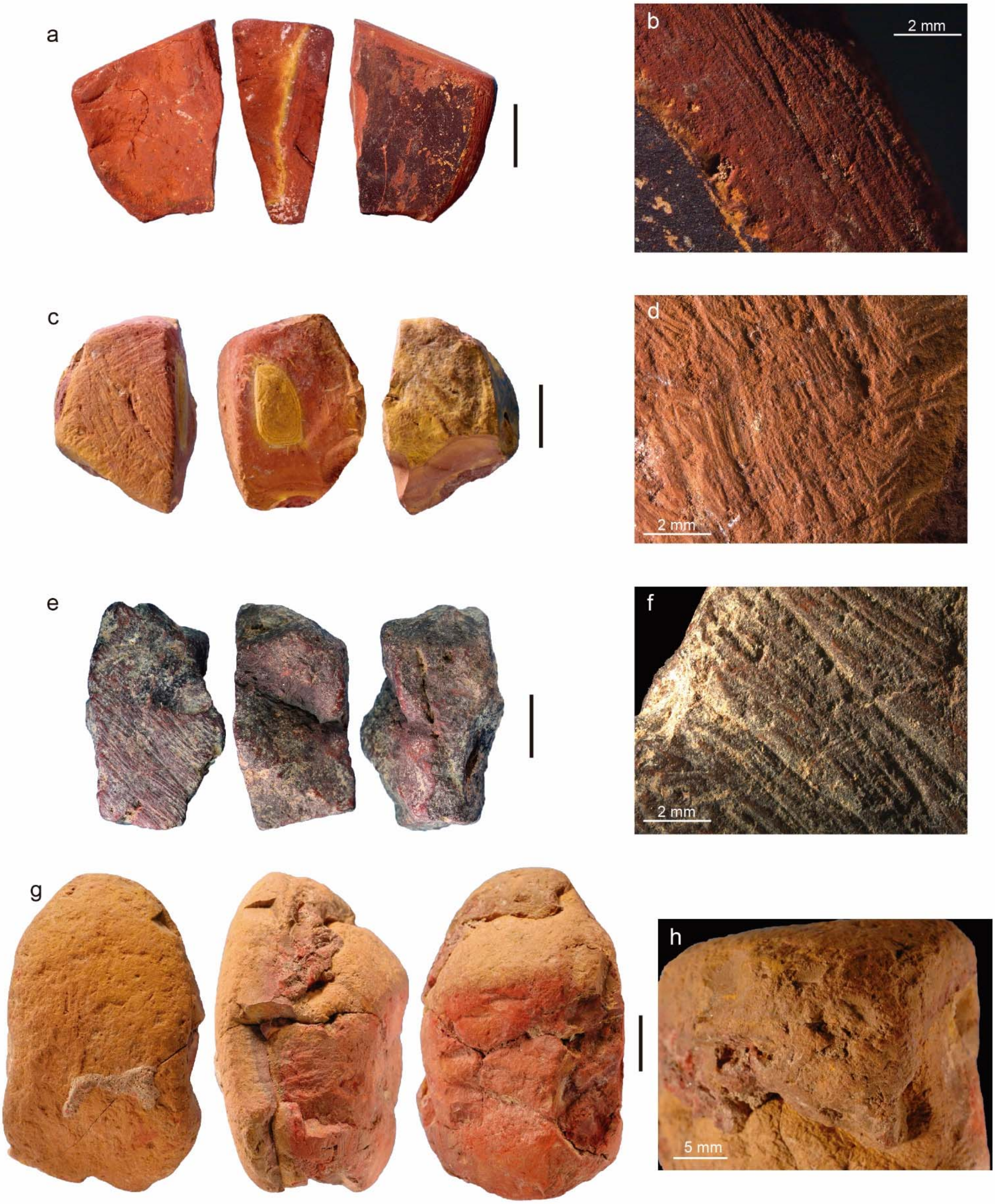

Fig C. Ochre pieces with traces produced by grinding and pitting. (a, b) Ochre piece PE1806 and photo of striations produced by grinding on the same piece, BFG. (c, d) Ochre piece PE2104 and photo of striations produced by grinding on the same piece, BFG. (e, f) Ochre piece PE1752 and photo of striations produced by grinding on the same piece, HFG. (g, h) Ochre piece PE931 (also OPT21) and photo of pits on the same piece, BFG.

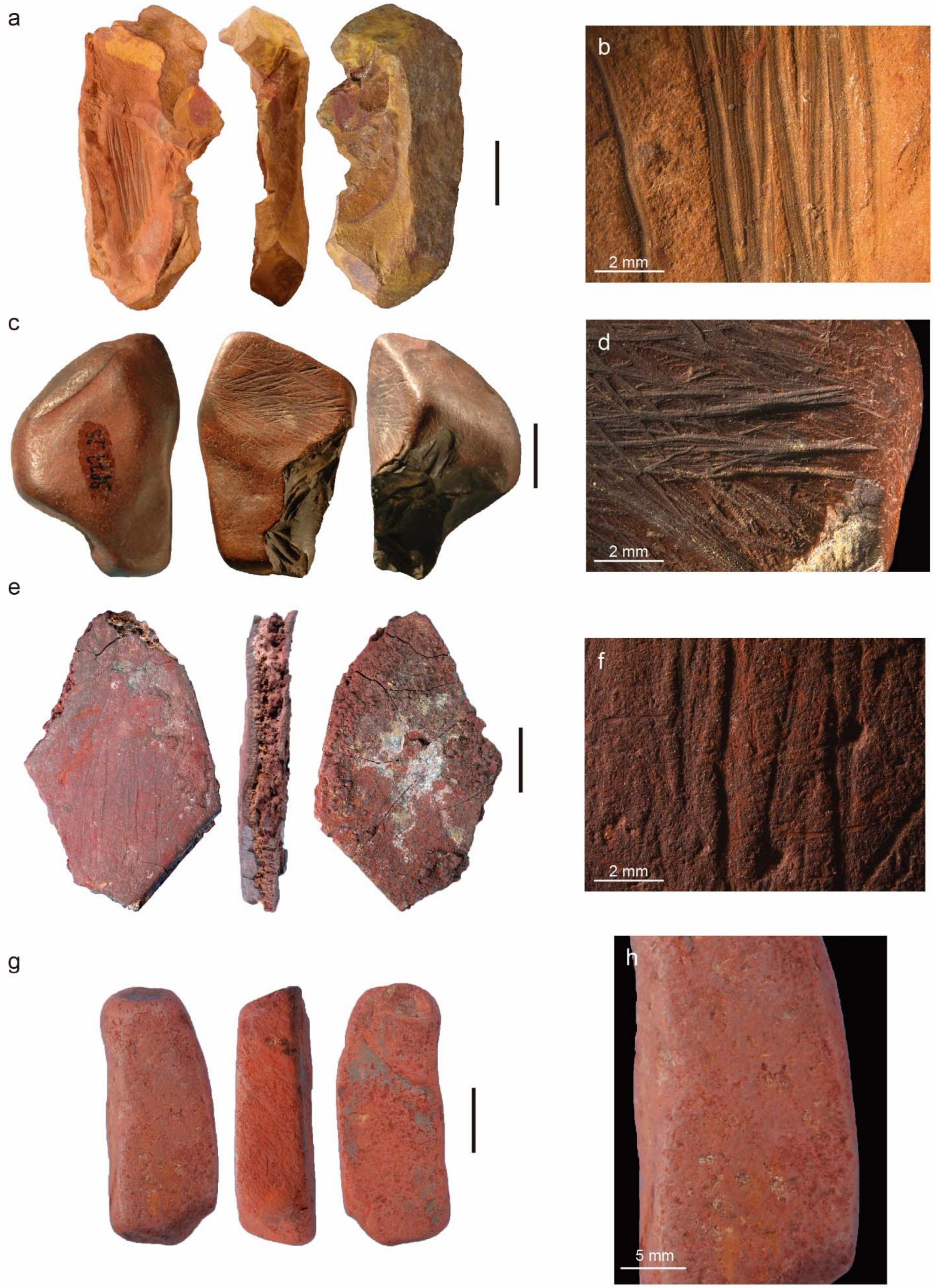

Fig D. Ochre pieces with traces produced by scraping and smoothed areas. (a, b) Ochre piece PE306 and photo of incisions produced by scraping on the same piece, BFG. (c, d) Ochre piece PE1419 and photo of incisions produced by scraping on the same piece, HFG. (e, f) Ochre piece PE1699 and photo of incisions produced by scraping on the same piece, SFG. (g, h) Ochre piece PE3067 and photo of smoothed areas, SFG.

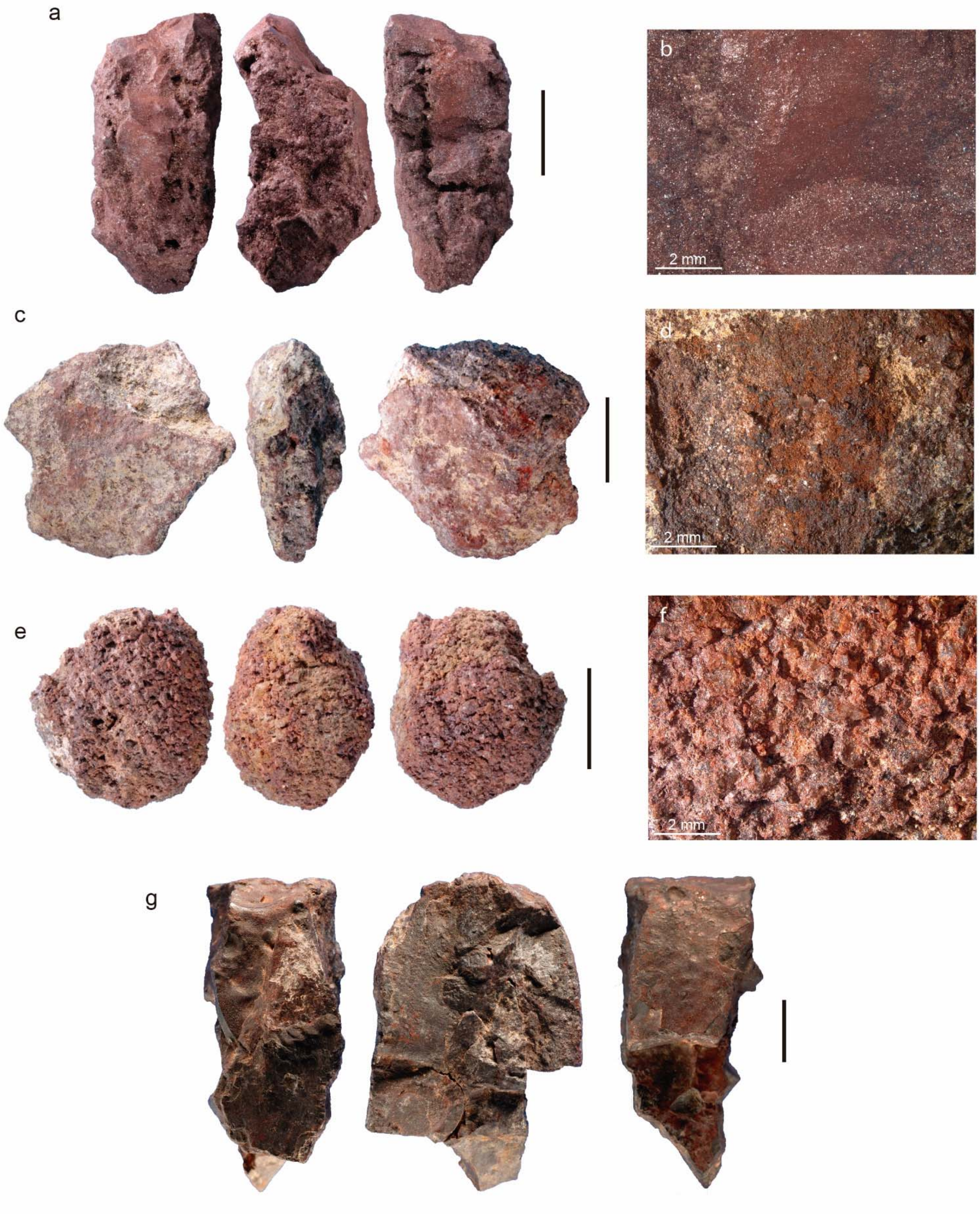

Fig E. Unmodified ochre pieces. (a, b) Ochre piece PE436, PFG. (c, d) Ochre piece PE809, CG. (e, f) Ochre piece PE1577, FS. (g) Ochre piece PE962, HFG.
