# Supplementary material for: Patterns of change and continuity in ochre use during the late Middle Stone Age of the Horn of Africa: The Porc-Epic Cave record
Source: PLoS One. 2017 May 24;12(5):e0177298. doi: 10.1371/journal.pone.0177298 (PMC5443497; doi:10.1371/journal.pone.0177298)
Supplement: S1 Tables — Colour, raw material and modifications of ochre pieces. (PDF) [file pone.0177298.s002.pdf]

Patterns of change and continuity in ochre use during the late Middle Stone Age of the Horn of Africa: the Porc-Epic Cave record

Daniela Eugenia Rosso*, Francesco d'Errico, Alain Queffelec

* Corresponding author
E-mail: d.rosso@pacea.u-bordeaux1.fr (DR)

S1 Tables. Detailed results of the technological analysis of ochre pieces.

Colour, raw material, and modifications of ochre pieces.

Table A. Vertical distribution of colours for ochre pieces. 2
Table B. Proportion of colours for ochre pieces 3
Table C. Colours by weight 4
Table D. Colours by raw material types 5
Table E. Colours by modification types 6
Table F. Vertical distribution of ochre raw material types 7
Table G. Vertical distribution of ochre raw material types by weight 8
Table H. Vertical distribution of ochre raw material types by weight in percentages 9
Table I. Vertical distribution of unmodified and modified ochre 10
Table J. Vertical distribution of unmodified and modified ochre by weight 11
Table K. Occurrence of each modification throughout the stratigraphy 12
Table L. Vertical distribution of cross-section of facets and orientation of striations 13
Table M. Vertical distribution of combinations of modifications 14
Table N. One-way ANOVA test on length of pieces by number of facets per piece 15
Table O. Pairwise comparison for length of pieces by number of facets with Bonferroni correction on length. 16
Table P. One-way ANOVA test on length of facets by number of facets per piece. 17
Table Q. Post-hoc Tamhane T2 test on pairwise comparison of length of facets by number of facets per piece. 18

Table A. Vertical distribution of colours for ochre piece.

Levels (cm)	Colours																											
	R	DR	R+G	R+Y	G	R+O	BR	DR+G	R+DR	Y+DR	O	O+DR	Y	R+BL	O+G	R+O+G	BR+Y	R+Y+G	Y+G	R+Y+[illegible]	R+BR	BR+G	R+O+BL	Y+O	BR+DR	BL+Y	R+Y+DR	BL	TOT
**30-40**	1	0	0	0	0	0	1	0	0	0	0	0	0	0	0	0	0	0	0	0	0	0	0	0	0	0	0	0	2
**40-50**	1	1	0	0	1	0	0	0	0	0	1	1	0	0	0	0	0	0	0	0	0	0	0	0	0	0	0	0	5
**50-60**	18	0	4	0	1	2	0	0	0	0	0	0	0	0	0	0	0	0	0	0	0	0	0	0	0	0	0	0	25
**60-70**	177	8	84	15	9	6	2	0	0	0	2	0	2	6	2	2	0	1	0	5	0	0	0	1	0	0	0	0	322
**70-80**	111	7	53	5	10	10	1	1	0	1	3	0	0	4	0	1	0	0	1	0	0	0	0	0	0	0	1	0	209
**80-90**	60	14	38	2	6	3	0	0	1	1	1	0	1	2	0	0	0	0	0	0	0	0	0	0	0	0	0	0	129
**90-100**	93	38	45	4	11	8	8	0	0	0	10	3	2	3	2	5	0	2	0	0	0	0	1	0	0	0	0	1	236
**100-110**	134	76	30	9	14	8	7	5	6	1	5	0	0	5	0	0	1	1	0	0	1	0	0	0	1	1	1	0	306
**110-120**	215	235	74	25	42	24	15	17	3	16	5	6	8	2	1	0	7	3	2	0	1	0	1	0	0	1	0	0	703
**120-130**	137	86	35	25	23	25	6	9	6	4	1	4	2	4	2	1	0	1	2	0	0	3	0	0	0	0	0	0	376
**130-140**	86	93	22	33	13	8	4	3	4	4	2	0	0	0	0	0	0	0	1	0	0	0	0	0	0	0	0	0	273
**140-150**	145	116	5	26	6	11	6	1	2	0	0	1	1	1	2	0	1	0	0	0	1	0	0	0	0	0	0	0	325
**150-160**	60	122	26	15	11	11	2	7	2	9	1	1	3	0	1	0	0	0	0	0	1	0	0	1	1	0	0	0	274
**160-170**	54	44	12	13	4	6	5	9	6	2	2	0	1	0	0	0	0	1	1	0	0	0	0	0	0	0	0	0	160
**170-180**	53	64	17	4	4	7	5	2	11	2	3	10	3	0	0	1	0	0	0	0	0	0	0	0	0	0	0	0	186
**180-190**	26	33	9	1	6	4	0	0	3	0	3	5	0	0	0	0	0	0	0	0	0	0	0	0	0	0	0	0	90
**190-200**	19	9	1	5	1	3	2	4	3	0	1	2	1	0	1	0	0	0	0	0	0	0	0	0	0	0	0	0	52
**200-210**	17	10	3	0	0	4	0	0	7	0	0	0	0	0	0	0	0	0	0	0	0	0	0	0	0	0	0	0	41
**210-220**	8	8	3	2	0	0	0	0	1	0	0	0	3	0	0	0	0	0	1	0	0	0	0	0	0	0	0	0	26
**220-230**	4	2	1	1	0	0	0	0	1	1	0	0	0	0	0	0	0	0	0	0	0	0	0	0	0	0	0	0	10
**230-240**	1	1	1	0	0	0	0	0	0	0	0	0	0	0	0	0	0	0	0	0	0	0	0	0	0	0	0	0	3
**240-250**	3	4	0	0	0	0	0	0	0	0	0	0	0	0	0	0	0	0	0	0	0	0	0	0	0	0	0	0	7
**250-260**	0	3	10	0	0	0	0	0	0	0	0	0	0	0	0	0	0	0	0	0	0	0	0	0	0	0	0	0	13
**260-270**	3	5	2	0	0	0	0	3	1	0	0	3	0	0	0	0	0	0	0	0	0	0	0	0	0	0	0	0	17
**270-280**	0	1	0	0	0	0	0	0	0	0	0	0	0	0	0	0	0	0	0	0	0	0	1	0	0	0	0	0	2
**TOTAL**	**1426**	**980**	**475**	**185**	**162**	**140**	**64**	**61**	**57**	**41**	**40**	**36**	**27**	**27**	**11**	**10**	**9**	**9**	**8**	**5**	**4**	**3**	**3**	**2**	**2**	**2**	**2**	**1**	**3792**

R: red; DR: dark red; G: grey; Y: yellow; O: orange; BR: brown; BL: black; TOT: total.

Table B. Proportion of colours for ochre pieces.

Levels (cm)	Colours																												
	R	DR	R+G	R+Y	G	R+O	BR	DR+G	R+DR	Y+DR	O	O+DR	Y	R+BL	O+G	R+O+G	BR+Y	R+Y+G	Y+G	R+Y+[illegible]	R+BR	BR+G	R+O+BL	Y+O	BR+DR	BL+Y	R+Y+DR	BL	TOT
**30-40**	50	0	0	0	0	0	50	0	0	0	0	0	0	0	0	0	0	0	0	0	0	0	0	0	0	0	0	0	100
**40-50**	20	20	0	0	20	0	0	0	0	0	20	20	0	0	0	0	0	0	0	0	0	0	0	0	0	0	0	0	100
**50-60**	72	0	16	0	4	8	0	0	0	0	0	0	0	0	0	0	0	0	0	0	0	0	0	0	0	0	0	0	100
**60-70**	55	2.5	26.1	4.7	2.8	1.9	0.6	0	0	0	0.6	0	0.6	1.9	0.6	0.6	0	0.3	0	1.6	0	0	0	0.3	0	0	0	0	100
**70-80**	53.1	3.3	25.4	2.4	4.8	4.8	0.5	0.5	0	0.5	1.4	0	0	1.9	0	0.5	0	0	0.5	0	0	0	0	0	0	0	0.5	0	100
**80-90**	46.5	10.9	29.5	1.6	4.7	2.3	0.0	0	0.8	0.8	0.8	0	0.8	1.6	0	0	0	0	0	0	0	0	0	0	0	0	0	0	100
**90-100**	39.4	16.1	19.1	1.7	4.7	3.4	3.4	0	0	0	4.2	1.3	0.8	1.3	0.8	2.1	0	0.8	0	0	0	0	0.4	0	0	0	0	0.4	100
**100-110**	43.8	24.8	9.8	2.9	4.6	2.6	2.3	1.6	2.0	0.3	1.6	0	0	1.6	0	0	0.3	0.3	0	0	0.3	0	0	0	0.3	0.3	0.3	0	100
**110-120**	30.6	33.4	10.5	3.6	6	3.4	2.1	2.4	0.4	2.3	0.7	0.9	1.1	0.3	0.1	0	1.0	0.4	0.3	0	0.1	0	0.1	0	0	0.1	0	0	100
**120-130**	36.4	22.9	9.3	6.6	6.1	6.6	1.6	2.4	1.6	1.1	0.3	1.1	0.5	1.1	0.5	0.3	0	0.3	0.5	0	0	0.8	0	0	0	0	0	0	100
**130-140**	31.5	34.1	8.1	12.1	4.8	2.9	1.5	1.1	1.5	1.5	0.7	0	0	0	0	0	0	0	0.4	0	0	0	0	0	0	0	0	0	100
**140-150**	44.6	35.7	1.5	8.0	1.8	3.4	1.8	0.3	0.6	0	0	0.3	0.3	0.3	0.6	0	0.3	0	0	0	0.3	0	0	0	0	0	0	0	100
**150-160**	21.9	44.5	9.5	5.5	4	4	0.7	2.6	0.7	3.3	0.4	0.4	1.1	0	0.4	0	0	0	0	0	0.4	0	0	0.4	0.4	0	0	0	100
**160-170**	33.8	27.5	7.5	8.1	2.5	3.8	3.1	5.6	3.8	1.3	1.3	0	0.6	0	0	0	0	0.6	0.6	0	0	0	0	0	0	0	0	0	100
**170-180**	28.5	34.4	9.1	2.2	2.2	3.8	2.7	1.1	5.9	1.1	1.6	5.4	1.6	0	0	0.5	0	0	0	0	0	0	0	0	0	0	0	0	100
**180-190**	28.9	36.7	10	1.1	6.7	4.4	0	0	3.3	0	3.3	5.6	0	0	0	0	0	0	0	0	0	0	0	0	0	0	0	0	100
**190-200**	36.5	17.3	1.9	9.6	1.9	5.8	3.8	7.7	5.8	0	1.9	3.8	1.9	0	1.9	0	0	0	0	0	0	0	0	0	0	0	0	0	100
**200-210**	41.5	24.4	7.3	0.0	0	9.8	0	0	17.1	0	0	0	0	0	0	0	0	0	0	0	0	0	0	0	0	0	0	0	100
**210-220**	30.8	30.8	11.5	7.7	0	0	0	0	3.8	0	0	0	11.5	0	0	0	0	0	3.8	0	0	0	0	0	0	0	0	0	100
**220-230**	40	20	10	10	0	0	0	0	10	10	0	0	0	0	0	0	0	0	0	0	0	0	0	0	0	0	0	0	100
**230-240**	33.3	33.3	33.3	0	0	0	0	0	0	0	0	0	0	0	0	0	0	0	0	0	0	0	0	0	0	0	0	0	100
**240-250**	42.9	57.1	0	0	0	0	0	0	0	0	0	0	0	0	0	0	0	0	0	0	0	0	0	0	0	0	0	0	100
**250-260**	0	23.1	76.9	0	0	0	0	0	0	0	0	0	0	0	0	0	0	0	0	0	0	0	0	0	0	0	0	0	100
**260-270**	17.6	29.4	11.8	0	0	0	0	17.6	5.9	0	0	17.6	0	0	0	0	0	0	0	0	0	0	0	0	0	0	0	0	100
**270-280**	0	50	0	0	0	0	0	0	0	0	0	0	0	0	0	0	0	0	0	0	0	0	50	0	0	0	0	0	100
**TOTAL**	**37.6**	**25.8**	**12.5**	**4.9**	**4.3**	**3.7**	**1.7**	**1.6**	**1.5**	**1.1**	**1.1**	**0.9**	**0.7**	**0.7**	**0.3**	**0.3**	**0.2**	**0.2**	**0.2**	**0.1**	**0.1**	**0.1**	**0.1**	**0.1**	**0.1**	**0.1**	**0.1**	**0**	**100**

Numbers are presented in percentages. R: red; DR: dark red; G: grey; Y: yellow; O: orange; BR: brown; BL: black; TOT: total.

Table C. Colours by weight.

Colour	Minimum	Maximum	Mean	St. dev.	Total weight	Number of pieces
**R**	0.1	678	10.049	29.398	14249.21	1426
**DR**	0.1	258.39	6.449	16.281	6313.155	980
**R+G**	0.13	364.5	14.914	26.438	7084.09	475
**R+Y**	0.1	172.2	14.492	24.656	2680.945	185
**G**	0.1	402.1	17.729	35.988	2872.13	162
**R+O**	0.18	87.03	12.698	16.745	1777.75	140
**BR**	0.1	110.88	6.191	14.716	396.205	64
**DR+G**	0.1	229.89	19.237	35.79	1173.48	61
**R+DR**	0.41	175.76	16.457	28.328	938.03	57
**Y+DR**	0.1	30.45	8.156	8.733	334.41	41
**O**	0.1	76.89	9.288	15.831	362.225	40
**O+DR**	0.12	89.3	9.117	15.187	328.2	36
**Y**	0.1	33.85	4.624	6.521	124.86	27
**R+BL**	0.7	33.05	9.305	7.8	251.24	27
**O+G**	0.5	36.88	7.61	10.216	83.71	11
**R+O+G**	1.71	79.57	23.812	28.857	238.12	10
**BR+Y**	0.1	12.07	2.691	5.161	24.215	9
**R+Y+G**	1.93	28.38	9.411	8.4	84.7	9
**Y+G**	1.09	123.44	28.365	40.518	226.92	8
**R+Y+O**	6.48	24.92	17.106	7.284	85.53	5
**R+BR**	5.9	15.35	10.453	4.561	41.81	4
**BR+G**	1.63	45.19	16.4	24.936	49.2	3
**R+O+BL**	3.16	48.62	18.883	25.767	56.65	3
**Y+O**	0.48	19.66	10.07	13.562	20.14	2
**BR+DR**	4.5	10.53	7.515	4.264	15.03	2
**BL+Y**	18.24	34.47	26.355	11.476	52.71	2
**R+Y+DR**	11.26	78.05	44.655	47.228	89.31	2
**BL**	24.46	24.46	24.46	-	24.46	1
**All pieces**	0.1	678	10.571	25.272	39978.4	3792

Data is in grams. R: red; DR: dark red; G: grey; Y: yellow; O: orange; BR: brown; BL: black; St. dev: standard deviation.

Table D. Colours by raw material types.

Colours	Raw material (n)						Num of pieces
	SFG	CG	BFG	HFG	FS	PFG	
**R**	1225	119	29	45	8	0	1426
**DR**	847	100	0	30	3	0	980
**R+G**	220	115	9	121	3	7	475
**R+Y**	32	5	147	1	0	0	185
**G**	25	55	0	80	1	1	162
**R+O**	37	6	92	2	3	0	140
**BR**	40	22	0	0	2	0	64
**DR+G**	27	22	0	12	0	0	61
**R+DR**	45	10	1	1	0	0	57
**Y+DR**	2	1	37	1	0	0	41
**O**	22	7	6	0	5	0	40
**O+DR**	13	2	21	0	0	0	36
**Y**	10	5	11	0	1	0	27
**R+BL**	14	3	0	10	0	0	27
**O+G**	4	5	0	0	2	0	11
**R+O+G**	5	3	1	1	0	0	10
**BR+Y**	0	1	7	1	0	0	9
**R+Y+G**	5	1	2	1	0	0	9
**Y+G**	2	3	2	1	0	0	8
**R+Y+O**	1	0	4	0	0	0	5
**R+BR**	2	0	0	0	2	0	4
**BR+G**	2	1	0	0	0	0	3
**R+O+BL**	2	0	1	0	0	0	3
**Y+O**	1	0	1	0	0	0	2
**BR+DR**	2	0	0	0	0	0	2
**BL+Y**	1	0	0	1	0	0	2
**R+Y+DR**	2	0	0	0	0	0	2
**BL**	0	0	0	1	0	0	1
**Total**	**2588**	**486**	**371**	**309**	**30**	**8**	**3792**

R: red; DR: dark red; G: grey; Y: yellow; O: orange; BR: brown; BL: black; SFG: Soft fine-grained; CG: coarse-grained; BFG: banded fine-grained; HFG: hard fine-grained; FS: ferruginous sandstone; PFG: platy fine-grained.

Table E. Colours by modification types.

Colours	FK	GR	SC	SM	P	Number of facets											Unmod	Num of pieces
						1	2	3	4	5	6	7	8	9	11	18		
R	465	361	56	34	5	157	78	45	16	15	11	6	1	0	1	0	744	1426
DR	306	227	14	13	1	120	44	26	7	3	3	3	0	1	0	0	563	980
R+G	132	86	7	2	5	31	21	8	5	4	3	1	0	0	0	0	293	475
R+Y	89	60	12	3	1	30	11	4	4	2	1	1	1	1	0	0	69	185
G	63	35	4	4	0	20	8	0	1	0	0	0	0	0	0	0	84	162
R+O	55	42	5	4	1	17	8	2	2	3	1	0	0	0	0	1	59	140
BR	16	16	0	1	0	6	4	4	0	1	0	0	0	0	0	0	41	64
DR+G	29	20	1	3	0	7	4	9	0	0	0	0	0	0	0	0	21	61
R+DR	17	18	3	0	0	10	2	2	0	1	0	0	0	0	0	0	27	57
Y+DR	17	14	1	2	0	9	3	0	1	0	0	0	0	0	0	0	16	41
O	13	4	1	1	0	2	0	1	0	0	0	0	0	0	0	0	26	40
O+DR	16	12	2	0	0	6	3	0	0	1	0	0	0	0	0	0	13	36
Y	6	4	3	2	0	4	0	0	0	0	0	0	0	0	0	0	18	27
R+BL	3	1	0	0	0	1	0	0	0	0	0	0	0	0	0	0	24	27
O+G	1	1	0	1	0	1	0	0	0	0	0	0	0	0	0	0	8	11
R+O+G	2	2	0	0	0	1	0	0	1	0	0	0	0	0	0	0	7	10
BR+Y	1	1	0	0	0	1	0	0	0	0	0	0	0	0	0	0	8	9
R+Y+G	2	2	2	0	0	1	1	0	0	0	0	0	0	0	0	0	6	9
Y+G	2	1	0	1	0	0	1	0	0	0	0	0	0	0	0	0	5	8
R+Y+O	2	0	0	0	0	0	0	0	0	0	0	0	0	0	0	0	3	5
R+BR	1	1	0	0	0	1	0	0	0	0	0	0	0	0	0	0	3	4
BR+G	1	0	0	0	0	0	0	0	0	0	0	0	0	0	0	0	2	3
R+O+BL	0	2	0	0	0	0	1	0	0	0	0	0	0	0	0	0	1	3
Y+O	0	1	0	0	0	0	1	0	0	0	0	0	0	0	0	0	1	2
BR+DR	1	1	0	0	0	0	0	0	1	0	0	0	0	0	0	0	0	2
BL+Y	1	0	0	0	0	0	0	0	0	0	0	0	0	0	0	0	1	2
R+Y+DR	0	1	0	0	1	1	0	0	0	0	0	0	0	0	0	0	1	2
BL	1	0	0	0	0	0	0	0	0	0	0	0	0	0	0	0	0	1
**Total**	**1242**	**913**	**111**	**71**	**14**	**426**	**190**	**101**	**38**	**30**	**19**	**11**	**2**	**2**	**1**	**1**	**2044**	**3792**

R: red; DR: dark red; G: grey; Y: yellow; O: orange; BR: brown; BL: black; FK: flaking; GR: grinding; SC: scraping; SM: smoothing; P: pitting; unmod.: unmodified; Num.: number.

Table F. Vertical distribution of ochre raw material types.

Levels (cm)	Raw material types					
	SFG	CG	BFG	HFG	FS	PFG
**30–40**	2	0	0	0	0	0
**40–50**	4	1	0	0	0	0
**50–60**	20	2	1	2	0	0
**60–70**	222	40	31	26	2	1
**70–80**	134	30	19	25	1	0
**80–90**	77	18	6	27	1	0
**90–100**	151	37	17	27	4	0
**100–110**	222	44	14	25	1	0
**110–120**	479	73	77	64	9	1
**120–130**	225	49	53	49	0	0
**130–140**	166	43	40	20	2	2
**140–150**	261	20	32	9	3	0
**150–160**	186	44	26	12	3	3
**160–170**	108	25	20	7	0	0
**170–180**	134	29	15	6	2	0
**180–190**	66	12	7	3	1	1
**190–200**	35	9	6	2	0	0
**200–210**	34	4	2	0	1	0
**210–220**	18	5	2	1	0	0
**220–230**	9	0	1	0	0	0
**230–240**	1	1	0	1	0	0
**240–250**	7	0	0	0	0	0
**250–260**	12	0	0	1	0	0
**260–270**	13	0	2	2	0	0
**270–280**	2	0	0	0	0	0
**Total**	**2588**	**486**	**371**	**309**	**30**	**8**

SFG: soft fine-grained; CG: coarse-grained; BFG: banded fine-grained; HFG: hard fine-grained; FS: ferruginous sandstone; PFG: platy fine-grained.

Table G. Vertical distribution of ochre raw material types by weight.

Levels (cm)	SFG (g)					CG (g)					BFG (g)					HFG (g)					FS (g)					PFG (g)				
	Min	Max	Mean	St. dev.	Tot	Min	Max	Mean	St. dev	Tot	Min	Max	Mean	St. dev.	Tot	Min	Max	Mean	St. dev.	Tot	Min	Max	Mean	St. dev.	Tot	Min	Max	Mean	St. dev.	Tot
**30–40**	9.8	13	11.3	2.086	22.59	-	-	-	-	-	-	-	-	-	-	-	-	-	-	-	-	-	-	-	-	-	-	-	-	-
**40–50**	0.9	11	5.7	4.784	22.8	43.2	43.2	43.22	-	43.22	-	-	-	-	-	-	-	-	-	-	-	-	-	-	-	-	-	-	-	-
**50–60**	0.5	67	7.101	14.67	142	2.97	3.42	3.195	0.318	6.39	4	4	4.02	-	4.02	14	17	15.46	1.683	30.92	-	-	-	-	-	-	-	-	-	-
**60–70**	0.2	168	6.161	14.439	1355	1.03	402	21.21	63.63	848.2	0.5	25	6.235	6.933	193.3	0.8	62	15.47	17.867	402.3	9.7	80	44.77	49.547	89.5	20	20	19.89	-	19.9
**70–80**	0.1	106	8.831	16.986	1183	1.17	279	31.05	61.05	931.5	0.3	48	13.59	13.436	258.3	0.6	101	18.75	20.827	468.7	16	16	16.09	.	16.1	-	-	-	-	-
**80–90**	0.2	188	10	21.986	770.3	2.33	36.5	12.94	11.03	233	3.2	10	6.905	2.897	41.43	1.3	56	14.46	13.653	390.4	4.8	4.8	4.82	.	4.82	-	-	-	-	-
**90–100**	0.1	320	9.276	28.825	1401	1.77	90.2	17.6	20.77	651.3	0.2	45	8.449	13.529	143.6	3.1	40	15.79	9.904	426.4	3.3	8.1	5.23	2.038	20.9	-	-	-	-	-
**100–110**	0.1	126	6.487	14.934	1434	0.2	230	24.23	41.16	1066	0.1	78	15.05	21.565	210.7	1.4	46	13.4	11.739	334.9	5.7	5.7	5.68	.	5.68	-	-	-	-	-
**110–120**	0.1	151	4.733	9.797	2267	0.04	87	16.33	19.9	1192	0.1	75	8.658	13.623	666.7	0.8	365	22.71	50.139	1453	0.4	17	6.708	4.736	60.4	14	14	14.4	-	14.4
**120–130**	0.1	109	8.464	14.064	1896	0.03	84.1	17.26	22.05	845.7	0	172	17.56	32.32	930.5	2	111	16.19	17.639	793.5	-	-	-	-	-	-	-	-	-	-
**130–140**	0.1	275	7.24	22.633	1202	0.07	188	14.89	37.4	640.5	0.1	27	6.427	6.91	257.1	0.9	53	18.1	16.978	362	0.8	13	6.83	8.57	13.7	2.9	8.7	5.81	4.13	11.6
**140–150**	0.1	278	7.618	24.989	1958	0.1	258	32.21	63.9	644.1	0.1	61	13.98	14.536	447.5	0.4	56	23.76	20.072	213.9	0	23	10.38	11.865	31.2	-	-	-	-	-
**150–160**	0	153	7.165	15.252	1318	0.1	678	31.89	102.6	1403	0.1	32	6.774	9.392	176.1	3.6	95	33.54	25.321	402.5	0.6	15	5.657	8.398	17	3.2	12	6.963	4.709	20.9
**160–170**	0.1	117	8.841	15.78	954.8	0.55	103	17.15	26.06	428.7	0.6	124	25.78	32.446	515.5	7.4	28	17.08	8.494	119.5	-	-	-	-	-	-	-	-	-	-
**170–180**	0.2	100	7.692	13.419	1031	0.1	91.4	26.91	26.93	780.4	1	26	5.231	6.192	78.47	4	73	29.22	25.081	175.3	4.5	7	5.73	1.782	11.5	-	-	-	-	-
**180–190**	0.4	187	12.32	26.121	813	2.49	132	25.19	34.5	302.2	1.1	23	7.134	8.846	49.94	9.5	36	20.52	14.119	61.57	4	4	4.03	-	4.03	13	13	12.64	-	12.6
**190–200**	0.2	111	13.77	21.408	481.9	3	36.7	12.2	11.12	109.8	2.5	11	7.268	3.69	43.61	19	23	21.11	2.758	42.22	-	-	-	-	-	-	-	-	-	-
**200–210**	0.3	42	7.255	9.766	246.7	1.58	15	6.453	5.877	25.81	1.7	7.9	4.815	4.391	9.63	-	-	-	-	-	42	42	41.59	-	41.6	-	-	-	-	-
**210–220**	0.2	15	5.582	4.937	100.5	0.37	176	54.25	74.04	271.2	36	49	42.49	8.768	84.98	70	70	70.13	-	70.13	-	-	-	-	-	-	-	-	-	-
**220–230**	1.3	90	16.38	28.894	147.4	-	-	-	-	-	20	20	20.03	-	20.03	-	-	-	-	-	-	-	-	-	-	-	-	-	-	-
**230–240**	8.5	8.5	8.5	-	8.5	3.58	3.58	3.58	-	3.58	-	-	-	-	-	192	192	192.4	-	192.4	-	-	-	-	-	-	-	-	-	-
**240–250**	1.2	13	6.399	4.527	44.79	-	-	-	-	-	-	-	-	-	-	-	-	-	-	-	-	-	-	-	-	-	-	-	-	-
**250–260**	0.3	13	3.682	3.363	44.18	-	-	-	-	-	-	-	-	-	-	7.7	7.7	7.68	-	7.68	-	-	-	-	-	-	-	-	-	-
**260–270**	0.4	94	13.59	25.504	176.7	-	-	-	-	-	0.7	5.7	3.19	3.564	6.38	19	20	19.6	0.375	39.19	-	-	-	-	-	-	-	-	-	-
**270–280**	3.2	7.3	5.23	2.927	10.46	-	-	-	-	-	-	-	-	-	-	-	-	-	-	-	-	-	-	-	-	-	-	-	-	-

SFG: Soft fine-grained; CG: coarse-grained; BFG: banded fine-grained; HFG: hard fine-grained; FS: ferruginous sandstone; PFG: platy fine-grained. Min: minimum; Max: maximum; st. dev: standard deviation.

Table H. Vertical distribution of ochre raw material types by weight in percentages.

Levels (cm)	SFG	CG	BFG	HFG	FS	PFG	Total
**30–40**	100	-	-	-	-	-	100
**40–50**	34.53	65.47	-	-	-	-	100
**50–60**	77.46	3.485	2.193	16.86	-	-	100
**60–70**	46.6	29.16	6.645	13.83	3.078	0.684	100
**70–80**	41.4	32.59	9.037	16.4	0.563	-	100
**80–90**	53.49	16.18	2.877	27.11	0.335	-	100
**90–100**	53	24.64	5.435	16.13	0.792	-	100
**100–110**	46.99	34.94	6.906	10.98	0.186	-	100
**110–120**	40.1	21.08	11.79	25.7	1.068	0.255	100
**120–130**	42.46	18.94	20.84	17.77	-	-	100
**130–140**	48.33	25.76	10.34	14.56	0.549	0.467	100
**140–150**	59.43	19.55	13.58	6.492	0.946	-	100
**150–160**	39.49	42.04	5.276	12.06	0.508	0.626	100
**160–170**	47.3	21.24	25.54	5.922	-	-	100
**170–180**	49.64	37.58	3.779	8.444	0.552	-	100
**180–190**	65.38	24.31	4.016	4.952	0.324	1.017	100
**190–200**	71.12	16.21	6.437	6.231	-	-	100
**200–210**	76.2	7.974	2.975	-	12.85	-	100
**210–220**	19.07	51.49	16.13	13.31	-	-	100
**220–230**	88.04	-	11.96	-	-	-	100
**230–240**	4.156	1.751	-	94.09	-	-	100
**240–250**	100	-	-	-	-	-	100
**250–260**	85.19	-	-	14.81	-	-	100
**260–270**	79.5	-	2.871	17.63	-	-	100
**270–280**	100	-	-	-	-	-	100

Data is presented in percentages. SFG: Soft fine-grained; CG: coarse-grained; BFG: banded fine-grained; HFG: hard fine-grained; FS: ferruginous sandstone; PFG: platy fine-grained.

Table I. Vertical distribution of unmodified and modified ochre.

Levels (cm)	Unmodified	Modified	Total
**30–40**	0	2	2
**40–50**	4	1	5
**50–60**	18	7	25
**60–70**	240	82	322
**70–80**	137	72	209
**80–90**	77	52	129
**90–100**	158	78	236
**100–110**	192	114	306
**110–120**	377	326	703
**120–130**	172	204	376
**130–140**	125	148	273
**140–150**	178	147	325
**150–160**	123	151	274
**160–170**	72	88	160
**170–180**	70	116	186
**180–190**	24	66	90
**190–200**	22	30	52
**200–210**	25	16	41
**210–220**	12	14	26
**220–230**	4	6	10
**230–240**	3	0	3
**240–250**	4	3	7
**250–260**	9	4	13
**260–270**	6	11	17
**270–280**	1	1	2
**Total**	**2053**	**1739**	**3792**

Table J. Vertical distribution of unmodified and modified ochre by weight.

Levels (cm)	Unmodified ochre (g)					Modified ochre (g)					All pieces (kg)
	Min	Max	Mean	St. dev.	Total	Min	Max	Mean	St. dev.	Total	
**30–40**	-	-	-	-	0	9.82	12.77	11.3	2.086	22.59	0.02
**40–50**	0.86	43.22	15.81	18.79	63.23	2.79	2.79	2.79	-	2.79	0.07
**50–60**	0.49	67.32	7.297	15.49	131.34	2.26	16.65	7.43	5.68	52.01	0.18
**60–70**	0.21	168.5	7.185	14.92	1710.12	0.24	402.1	14.62	45.96	1198.42	2.91
**70–80**	0.05	105.6	9.947	16.92	1362.72	0.29	279.5	20.77	42.54	1495.19	2.86
**80–90**	0.24	55.63	8.909	10.88	686.01	0.4	188	14.5	25.95	753.87	1.44
**90–100**	0.07	320.4	10.03	27.93	1584.65	0.18	89.3	13.57	18.17	1058.21	2.64
**100–110**	0.1	229.9	7.341	20.48	1402.165	0.12	128.3	14.46	23.09	1648.85	3.05
**110–120**	0.1	181.9	6.707	16.21	2528.56	0.02	364.5	9.586	22.93	3125.18	5.65
**120–130**	0.1	172.2	7.611	18.37	1309.09	0.13	111.5	15.55	19.88	3156.56	4.47
**130–140**	0.1	27.2	3.356	4.726	419.475	0.04	275.1	13.97	31.33	2067.2	2.49
**140–150**	0.1	58.1	3.032	7.082	530.57	0.005	277.8	18.93	40.01	2763.855	3.29
**150–160**	0.1	76.89	5.334	11.48	650.78	0.06	678	17.92	58.29	2687.29	3.34
**160–170**	0.1	90.8	6.867	12.64	494.42	0.18	124.3	17.32	24.77	1524.12	2.02
**170–180**	0.1	61.95	5.712	9.913	399.82	0.64	100.3	14.45	20.68	1676.59	2.08
**180–190**	0.41	132.2	13.55	28.75	325.21	0.66	187.2	13.91	25.28	918.19	1.24
**190–200**	0.82	31.76	10.6	8.962	233.1	0.16	110.9	14.81	22.81	444.43	0.68
**200–210**	0.28	41.71	7.738	11.58	193.46	0.34	28.15	8.139	9.036	130.23	0.32
**210–220**	0.24	70.13	9.895	19.74	118.74	0.81	175.8	29.15	47.13	408.08	0.53
**220–230**	3.83	89.69	32.49	39.77	129.96	1.27	20.03	6.25	7.652	37.5	0.17
**230–240**	3.58	192.4	68.17	107.6	204.51	-	-	-	-	0	0.20
**240–250**	1.51	13.09	7.153	5.177	28.61	1.23	9.85	5.393	4.317	16.18	0.04
**250–260**	0.32	4.84	2.182	1.353	19.64	5.86	12.56	8.055	3.109	32.22	0.05
**260–270**	0.88	94.35	23.22	35.61	139.3	0.35	23.88	7.54	8.986	82.94	0.22
**270–280**	7.3	7.3	7.3	-	7.3	3.16	3.16	3.16	.-	3.16	0.01
**Total**	**0.005**	**320.4**	**7.171**	**17.1**	**14672.78**	**0.005**	**678**	**14.58**	**31.9**	**25305.66**	**39.9**

Min: minimum; max: maximum; st. dev: standard deviation.

Table K. Occurrence of each modification throughout the stratigraphy.

Levels (cm)	Modifications (n)						Modifications (%)					
	FK	G	SC	SM	P	Tot	FK	G	SC	SM	P	Tot
**30–40**	1	2	0	0	0	3	33	67	0	0	0	100
**40–50**	1	0	0	0	0	1	100	0	0	0	0	100
**50–60**	6	1	0	0	0	7	86	14	0	0	0	100
**60–70**	59	21	6	1	3	90	66	23	6.7	1.1	3.3	100
**70–80**	57	21	6	3	3	90	63	23	6.7	3.3	3.3	100
**80–90**	40	20	7	1	1	69	58	29	10	1.4	1.4	100
**90–100**	59	30	3	4	1	97	61	31	3.1	4.1	1	100
**100–110**	87	45	9	2	1	144	60	31	6.3	1.4	0.7	100
**110–120**	238	176	29	14	1	458	52	38	6.3	3.1	0.2	100
**120–130**	144	123	11	12	1	291	49	42	3.8	4.1	0.3	100
**130–140**	97	88	10	6	1	202	48	44	5	3	0.5	100
**140–150**	103	85	9	9	1	207	50	41	4.3	4.3	0.5	100
**150–160**	104	91	5	5	0	205	51	44	2.4	2.4	0	100
**160–170**	60	52	3	5	0	120	50	43	2.5	4.2	0	100
**170–180**	82	75	0	6	0	163	50	46	0	3.7	0	100
**180–190**	43	38	5	3	0	89	48	43	5.6	3.4	0	100
**190–200**	17	20	5	0	0	42	40	48	12	0	0	100
**200–210**	13	8	2	0	0	23	57	35	8.7	0	0	100
**210–220**	10	9	1	0	1	21	48	43	4.8	0	4.8	100
**220–230**	4	4	0	0	0	8	50	50	0	0	0	100
**230–240**	0	0	0	0	0	0	0	0	0	0	0	0
**240–250**	3	0	0	0	0	3	100	0	0	0	0	100
**250–260**	4	0	0	0	0	4	100	0	0	0	0	100
**260–270**	10	3	0	0	0	13	77	23	0	0	0	100
**270–280**	0	1	0	0	0	1	0	100	0	0	0	100
**Total**	**1242**	**913**	**111**	**71**	**14**	**2351**	**53**	**39**	**4.7**	**3**	**0.6**	**100**

FK: flaking; G: grinding; SC: scraping; SM: smoothing; P: pitting, tot: total.

Table L. Vertical distribution of cross-section of facets and orientation of striations.

Levels (cm)	Facet cross-section					Orientation of striations								Total (n)
	Conv	Flat	Conc	Irreg	Undet	O	L	O+L	P	R	O+P	L+P	Undet	
**30–40**	3	3	0	0	0	4	1	0	1	0	0	0	0	6
**40–50**	0	0	0	0	0	0	0	0	0	0	0	0	0	0
**50–60**	2	1	0	0	0	1	1	1	0	0	0	0	0	3
**60–70**	18	7	0	0	0	12	9	0	3	0	0	0	1	25
**70–80**	22	8	1	0	0	10	14	1	2	2	0	0	2	31
**80–90**	20	15	2	0	5	13	11	6	2	1	1	1	7	42
**90–100**	36	19	0	0	0	28	19	2	3	1	1	0	1	55
**100–110**	50	31	2	0	0	42	28	6	4	2	1	0	0	83
**110–120**	195	96	1	4	4	161	89	22	19	3	0	1	5	300
**120–130**	155	56	3	3	5	123	50	26	7	8	1	1	6	222
**130–140**	148	47	2	1	0	118	38	24	11	2	3	0	2	198
**140–150**	125	32	2	2	3	87	46	16	7	4	1	0	3	164
**150–160**	122	32	4	2	0	94	43	12	7	3	1	0	0	160
**160–170**	78	24	1	1	0	57	26	13	5	2	1	0	0	104
**170–180**	96	26	1	3	0	64	31	19	4	6	1	0	1	126
**180–190**	71	15	0	0	0	52	14	13	0	7	0	0	0	86
**190–200**	17	6	0	0	0	9	9	4	0	0	1	0	0	23
**200–210**	13	3	0	0	0	11	4	0	1	0	0	0	0	16
**210–220**	9	4	0	0	0	6	3	2	1	0	0	0	1	13
**220–230**	3	3	0	0	0	5	0	0	1	0	0	0	0	6
**230–240**	0	0	0	0	0	0	0	0	0	0	0	0	0	0
**240–250**	0	0	0	0	0	0	0	0	0	0	0	0	0	0
**250–260**	0	0	0	0	0	0	0	0	0	0	0	0	0	0
**260–270**	1	1	0	0	0	1	1	0	0	0	0	0	0	2
**270–280**	0	0	0	0	0	0	0	0	0	0	0	0	0	0
**Total**	**1184**	**429**	**19**	**16**	**17**	**898**	**437**	**167**	**78**	**41**	**12**	**3**	**29**	**1665**

Conv: convex; Conc: concave; irreg: irregular; undet: undetermined; O: oblique; L: longitudinal; P: perpendicular; R: random.

Table M. Vertical distribution of combinations of modifications.

Levels (cm)	Modifications																					
	FK	G	FK+G	G+SC	FK+G+SC	FK+SM	SC	G+SM	SM	FK+G+SM	FK+SC	FK+G+SC+SM	P+G	P	SC+SM	FK+G+P	FK+P	G+SC+SM	FK+G+P+SC	FK+G+P+SC+SM	FK+P+SC	TOT
**30–40**	0	1	1	0	0	0	0	0	0	0	0	0	0	0	0	0	0	0	0	0	0	**2**
**40–50**	1	0	0	0	0	0	0	0	0	0	0	0	0	0	0	0	0	0	0	0	0	**5**
**50–60**	6	1	0	0	0	0	0	0	0	0	0	0	0	0	0	0	0	0	0	0	0	**25**
**60–70**	54	16	2	2	0	0	3	0	1	0	1	0	1	0	0	0	2	0	0	0	0	**322**
**70–80**	47	11	5	0	3	1	1	0	1	0	0	0	1	1	0	0	0	0	0	1	0	**209**
**80–90**	29	8	7	2	2	0	1	1	0	0	1	0	0	0	0	0	0	0	0	0	1	**129**
**90–100**	45	15	10	1	1	1	1	1	0	2	0	0	0	1	0	0	0	0	0	0	0	**236**
**100–110**	66	23	15	1	3	0	1	0	0	0	2	1	1	0	0	0	0	1	0	0	0	**306**
**110–120**	138	66	82	12	9	4	4	3	2	2	0	2	0	0	1	0	0	0	1	0	0	**703**
**120–130**	74	48	59	4	5	3	1	5	2	2	1	0	0	0	0	0	0	0	0	0	0	**376**
**130–140**	55	40	37	7	1	2	1	1	2	0	0	1	0	0	0	1	0	0	0	0	0	**273**
**140–150**	55	35	39	4	2	2	1	1	2	3	1	0	0	0	1	1	0	0	0	0	0	**325**
**150–160**	57	42	42	2	2	2	1	2	0	1	0	0	0	0	0	0	0	0	0	0	0	**274**
**160–170**	31	26	23	0	3	3	0	0	2	0	0	0	0	0	0	0	0	0	0	0	0	**160**
**170–180**	37	33	40	0	0	3	0	0	1	2	0	0	0	0	0	0	0	0	0	0	0	**186**
**180–190**	24	19	15	0	2	0	1	2	1	0	2	0	0	0	0	0	0	0	0	0	0	**90**
**190–200**	9	10	6	2	2	0	1	0	0	0	0	0	0	0	0	0	0	0	0	0	0	**52**
**200–210**	6	3	5	0	0	0	0	0	0	0	2	0	0	0	0	0	0	0	0	0	0	**41**
**210–220**	5	2	5	1	0	0	0	0	0	0	0	0	1	0	0	0	0	0	0	0	0	**26**
**220–230**	2	2	2	0	0	0	0	0	0	0	0	0	0	0	0	0	0	0	0	0	0	**10**
**230–240**	0	0	0	0	0	0	0	0	0	0	0	0	0	0	0	0	0	0	0	0	0	**3**
**240–250**	3	0	0	0	0	0	0	0	0	0	0	0	0	0	0	0	0	0	0	0	0	**7**
**250–260**	4	0	0	0	0	0	0	0	0	0	0	0	0	0	0	0	0	0	0	0	0	**13**
**260–270**	8	1	2	0	0	0	0	0	0	0	0	0	0	0	0	0	0	0	0	0	0	**17**
**270–280**	0	1	0	0	0	0	0	0	0	0	0	0	0	0	0	0	0	0	0	0	0	**2**
**Total**	**756**	**403**	**397**	**38**	**35**	**21**	**17**	**16**	**14**	**12**	**10**	**4**	**4**	**2**	**2**	**2**	**2**	**1**	**1**	**1**	**1**	**3792**

FK: flaking; G: grinding; SC: scraping; SM: smoothing; P: pitting; TOT: total.

Table N. One-way ANOVA test on length of pieces by number of facets per piece.

	Sum of squares	DF	Mean squares	F	Significance
**Between groups**	8283.757	6	1380.626	9.622	**0.000**
**Within groups**	110769.952	772	142.484		
**Total**	119053.709	778			

We took into account pieces with one to 7 facets, as pieces with 8, 9, 11 and 18 facets have less than three specimens. The analysis was conducted with IBM SPSS Statistics for Macintosh, Version 21.0.

Table O. Pairwise comparison for length of pieces by number of facets with Bonferroni correction on length.

		1 Facet	2 facets	3 facets	4 facets	5 facets	6 facets	7 facets
**Mean squares**	**1 facet**		**-4.00439**	-3.79588	**-10.37742**	**-8.79108**	**-10.25618**	-9.67493
**Standard error**			**1.07023**	1.38745	**2.05578**	**2.30130**	**2.81071**	3.65955
**P-value**			**0.004**	0.134	**0.000**	**0.003**	**0.006**	0.176
**Mean squares**	**2 facets**	**4.00439**		0.20851	-6.37303	-4.78669	-6.25179	-5.67055
**Standard error**		**1.07023**		1.54074	2.16220	2.39685	2.88945	3.72037
**P-value**		**0.004**		1.000	0.069	0.970	0.647	1.000
**Mean squares**	**3 facets**	3.79588	-0.20851		-6.58153	-4.99520	-6.46029	-5.87905
**Standard error**		1.38745	1.54074		2.33553	2.55431	3.02135	3.82371
**P-value**		0.134	1.000		0,104	1.000	0.689	1.000
**Mean squares**	**4 facets**	**10.37742**	6.37303	6.58153		1.58634	0.12124	0.70248
**Standard error**		**2.05578**	2.16220	2.33553		2.97081	3.38079	4.11363
**P-value**		**0.000**	0.069	0,104		1.000	1.000	1.000
**Mean squares**	**5 facets**	**8.79108**	4.78669	4.99520	-1.58634		-1.46510	-0.88386
**Standard error**		**2.30130**	2.39685	2.55431	2.97081		3.53547	4.24167
**P-value**		**0.003**	0.970	1.000	1.000		1.000	1.000
**Mean squares**	**6 facets**	**10.25618**	6.25179	6.46029	-0.12124	1.46510		0.58124
**Standard error**		**2.81071**	2.88945	3.02135	3.38079	3.53547		4.53826
**P-value**		**0.006**	0.647	0.689	1.000	1.000		1.000
**Mean squares**	**7 facets**	9.67493	5.67055	5.87905	-0.70248	0.88386	-0.58124	
**Standard error**		3.65955	3.72037	3.82371	4.11363	4.24167	4.53826	
**P-value**		0.176	1.000	1.000	1.000	1.000	1.000	

We took into account pieces with one to 7 facets, as pieces with 8, 9, 11 and 18 facets have less than three specimens. In bold, statistically significant differences between pairs. The analysis was conducted with IBM SPSS Statistics for Macintosh, Version 21.0.

Table P. One-way ANOVA test on length of facets by number of facets per piece.

	Sum of squares	DF	Mean squares	F	Significance
**Between groups**	4116.710	10	411.671	3.942	**0.000**
**Within groups**	164887.406	1579	104.425		
**Total**	169004.117	1589			

The analysis was conducted with IBM SPSS Statistics for Macintosh, Version 21.0.

Table Q. Post-hoc Tamhane T2 test on pairwise comparison of length of facets by number of facets per piece.

		1 Facet	2 facets	3 facets	4 facets	5 facets	6 facets	7 facets	8 facets	9 facets	11 facets	18 facets
**Mean squares**			-1.28484	-0.08871	**-3.46324**	**-2.97576**	**-3.51013**	-2.41593	4.76353	-0.70633	-2.00204	**4.86700**
**Standard error**	**1 facet**		0.73960	0.79276	**0.96946**	**1.00205**	**1.08488**	1.26847	2.60373	2.46054	3.12187	**2.46054**
**P-value**			0.083	0.911	**0.000**	**0.003**	**0.001**	0.057	0.068	0.774	0.521	**0.048**
**Mean squares**		1.28484		1.19613	**-2.17841**	-1.69092	**-2.22529**	-1.13109	**6.04837**	0.57851	-0.71720	**6.15184**
**Standard error**	**2 facets**	0.73960		0.81840	**0.99054**	1.02246	**1.10375**	1.28465	**2.61165**	2.46892	3.12848	**2.46892**
**P-value**		0.083		0.144	**0.028**	0.098	**0.044**	0.379	**0.021**	0.815	0.819	**0.013**
**Mean squares**		0.08871	-1.19613		**-3.37454**	**-2.88705**	**-3.42142**	-2.32723	4.85224	-0.61762	-1.91333	**4.95571**
**Standard error**	**3 facets**	0.79276	0.81840		**1.03084**	**1.06155**	**1.14006**	1.31598	2.62720	2.48536	3.14147	**2.48536**
**P-value**		0.911	0.144		**0.001**	**0.007**	**0.003**	0.077	0.065	0.804	0.543	**0.046**
**Mean squares**		**3.46324**	**2.17841**	**3.37454**		0.48749	-0.04689	1.04731	**8.22678**	2.75692	1.46121	**8.33025**
**Standard error**	**4 facets**	**0.96946**	**0.99054**	**1.03084**		1.19928	1.26930	1.42940	**2.68581**	2.54724	3.19064	**2.54724**
**P-value**		**0.000**	**0.028**	**0.001**		0.684	0.971	0.464	**0.002**	0.279	0.647	**0.001**
**Mean squares**		**2.97576**	1.69092	**2.88705**	-0.48749		-0.53437	0.55983	**7.73929**	2.26943	0.97372	**7.84276**
**Standard error**	**5 facets**	**1.00205**	1.02246	**1.06155**	1.19928		1.29436	1.45170	**2.69775**	2.55982	3.20070	**2.55982**
**P-value**		**0.003**	0.098	**0.007**	0.684		0.680	0.700	**0.004**	0.375	0.761	**0.002**
**Mean squares**		**3.51013**	**2.22529**	**3.42142**	0.04689	0.53437		1.09420	**8.27366**	2.80380	1.50809	**8.37713**
**Standard error**	**6 facets**	**1.08488**	**1.10375**	**1.14006**	1.26930	1.29436		1.51006	**2.72960**	2.59336	3.22759	**2.59336**
**P-value**		**0.001**	**0.044**	**0.003**	0.971	0.680		0.469	**0.002**	0.280	0.640	**0.001**
**Mean squares**		2.41593	1.13109	2.32723	-1.04731	-0.55983	-1.09420		**7.17946**	1.70960	0.41390	**7.28294**
**Standard error**	**7 facets**	1.26847	1.28465	1.31598	1.42940	1.45170	1.51006		**2.80762**	2.67536	3.29384	**2.67536**
**P-value**		0.057	0.379	0.077	0.464	0.700	0.469		**0.011**	0.523	0.900	**0.007**
**Mean squares**		-4.76353	**-6.04837**	-4.85224	**-8.22678**	**-7.73929**	**-8.27366**	**-7.17946**		-5.46986	-6.76557	0.10347
**Standard error**	**8 facets**	2.60373	**2.61165**	2.62720	**2.68581**	**2.69775**	**2.72960**	**2.80762**		3.51112	4.00247	3.51112
**P-value**		0.068	**0.021**	0.065	**0.002**	**0.004**	**0.002**	**0.011**		0.119	0.091	0.976
**Mean squares**		0.70633	-0.57851	0.61762	-2.75692	-2.26943	-2.80380	-1.70960	5.46986		-1.29571	5.57333
**Standard error**	**9 facets**	2.46054	2.46892	2.48536	2.54724	2.55982	2.59336	2.67536	3.51112		3.91083	3.40629
**P-value**		0.774	0.815	0.804	0.279	0.375	0.280	0.523	0.119		0.740	0.102
**Mean squares**		2.00204	0.71720	1.91333	-1.46121	-0.97372	-1.50809	-0.41390	6.76557	1.29571		6.86904
**Standard error**	**11 facets**	3.12187	3.12848	3.14147	3.19064	3.20070	3.22759	3.29384	4.00247	3.91083		3.91083
**P-value**		0.521	0.819	0.543	0.647	0.761	0.640	0.900	0.091	0.740		0.079
**Mean squares**		**4.86700**	**-6.15184**	**-4.95571**	**-8.33025**	**-7.84276**	**-8.37713**	**-7.28294**	-0.10347	5.57333	-6.86904	
**Standard error**	**18 facets**	**2.46054**	**2.46892**	**2.48536**	**2.54724**	**2.55982**	**2.59336**	**2.67536**	3.51112	3.40629	3.91083	
**P-value**		**0.048**	**0.013**	**0.046**	**0.001**	**0.002**	**0.001**	**0.007**	0.976	0.102	0.079	

In bold, statistically significant differences between pairs. The analysis was conducted with IBM SPSS Statistics for Macintosh, Version 21.0.
